# Evolution and dispersal of snakes across the Cretaceous-Paleogene mass extinction

**DOI:** 10.1038/s41467-021-25136-y

**Published:** 2021-09-14

**Authors:** Catherine G. Klein, Davide Pisani, Daniel J. Field, Rebecca Lakin, Matthew A. Wills, Nicholas R. Longrich

**Affiliations:** 1 The Milner Centre for Evolution, Department of Biology and Biochemistry, University of Bath, Bath, UK; 2 School of Earth Sciences, School of Biological Sciences, University of Bristol, Bristol, UK; 3 Department of Earth Sciences, University of Cambridge, Cambridge, Cambridgeshire UK; 4 Present Address: GeoZentrum Nordbayern, Friedrich-Alexander University Erlangen-Nürnberg, Loewenichstr. 28, Erlangen, Germany

**Keywords:** Palaeontology, Phylogenetics

## Abstract

Mass extinctions have repeatedly shaped global biodiversity. The Cretaceous-Paleogene (K-Pg) mass extinction caused the demise of numerous vertebrate groups, and its aftermath saw the rapid diversification of surviving mammals, birds, frogs, and teleost fishes. However, the effects of the K-Pg extinction on the evolution of snakes—a major clade of predators comprising over 3,700 living species—remains poorly understood. Here, we combine an extensive molecular dataset with phylogenetically and stratigraphically constrained fossil calibrations to infer an evolutionary timescale for Serpentes. We reveal a potential diversification among crown snakes associated with the K-Pg mass extinction, led by the successful colonisation of Asia by the major extant clade Afrophidia. Vertebral morphometrics suggest increasing morphological specialisation among marine snakes through the Paleogene. The dispersal patterns of snakes following the K-Pg underscore the importance of this mass extinction event in shaping Earth’s extant vertebrate faunas.

## Abstract (web summary)

Snakes are one of the most successful groups of living vertebrates, but the timing of their diversification is unclear. Combining molecular clocks, fossils, and biogeography, Klein et al. show that snakes experienced a diversification, and underwent dispersal, around the time of the end-Cretaceous mass extinction.

## Introduction

The Cretaceous-Paleogene (K-Pg) transition resulted in the loss of an estimated 76% of all species^1,2^. High-resolution records of fossil pollen and marine microfossils show that the K-Pg extinction coincided with the Chicxulub bolide impact in Mexico^3,4^, which generated shockwaves, tsunamis, and a brief thermal pulse caused by the re-entry of heated ejecta in its immediate aftermath^4,5^. Critically, sulfates, dust, and hydrocarbon soot ejected into the atmosphere are thought to have greatly reduced insolation, resulting in global cooling over a period of years^4^. This would have caused a global collapse of photosynthesis^4^, resulting in high levels of extinction across the tree of life. Among vertebrates, iconic Mesozoic groups such as non-avian dinosaurs^6^, pterosaurs^7^, and mosasaurs^8^ disappeared at the K-Pg boundary. Other clades persisted but suffered severe reductions in diversity, including birds^9^, mammals^2^, and squamates^10^. Studies of insect feeding traces also indicate major losses among herbivorous arthropods^11^, whereas land plants suffered species-level extinctions of 80% or more^12^. Despite the magnitude of the impact and subsequent extinctions, terrestrial ecosystems recovered rapidly. In the aftermath, a “fern spike” followed by pioneer angiosperm communities documents the recovery of Earth’s flora^13,14^, while surviving vertebrate groups, including mammals^15,16^, birds^17–19^, frogs^20^, and teleost fishes^21,22^, recovered and rapidly radiated in the early Cenozoic.

Historically, squamates were believed to have experienced minimal extinction at the K-Pg boundary^23^. However, analysis of the K-Pg transition in western North America found evidence for high rates of extinction among squamates^10^, although it remains unclear whether this pattern holds on a global scale. The evolutionary history of snakes across the K-Pg boundary has been particularly difficult to assess. The early fossil record of crown group snakes is fragmentary, often restricted to vertebrae and afflicted by relatively high rates of homoplasy^24^. As a result, phylogenetic analyses largely rely on restricted character sets, which is frequently misleading for vertebrates^25–28^, complicating our understanding of the affinities of many fossil snakes and possibly obscuring macroevolutionary patterns across the K-Pg boundary.

Molecular divergence dating efforts across Squamata suggest post-Cretaceous diversifications of major clades such as lacertids^29^ and amphisbaenians^30^. So far, molecular divergence time analyses of snakes recover conflicting patterns. Most studies^31–34^ suggest that the majority of extant snake clades diverged in the Cretaceous, although several analyses hint at a more recent diversification of the major subclade Alethinophidia^30,35,36^. Given this uncertainty, we attempted to improve our understanding of the timescale of crown snake diversification and the methodological factors that affect these inferences.

In this study, we investigate the effects of a plurality of modelling approaches—alternative calibration schemes (one developed here, in addition to that of Head et al.^37,38^, as well as variations on both), clock models (uncorrelated vs. autocorrelated), and associated priors (uniform, skew-T, and skew-Normal)—upon inferred node ages within crown snakes. Our results suggest a potential diversification of snakes near the time of the K-Pg transition. We also explore the effect of the K-Pg extinction event on vertebral morphological disparity, in order to directly incorporate an extensive sample of fossil taxa, while also benefiting from the tendency for morphological disparity indices to be less sensitive to sampling biases than diversity estimates^39^. We find a pattern of increasing vertebral disparity in the aftermath of the extinction, with concurrent increases in average and maximum body size, and dispersal to previously unoccupied landmasses.

## Results

### Divergence time estimation

**Fig. 1:**
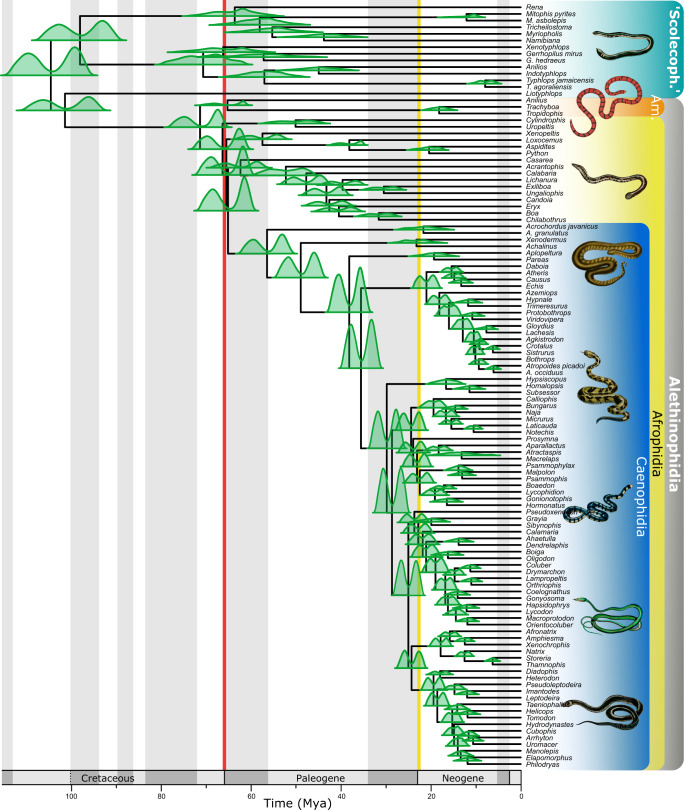
Time-calibrated phylogeny of crown snakes based on the 42 fossil calibration set developed in this study, under a Bayesian Inference framework. Green curves represent concatenated posterior age estimates for ingroup nodes using a skew-T and skew-Normal prior. Major clades discussed in the main text are shown on the right; Scolecoph., Scolecophidia; Am., Amerophidia. The K-Pg boundary is indicated by a red line and the Oligocene-Miocene boundary by a yellow line.

Our inferred tree topology is largely congruent with previous analyses^32^. Using our calibration set, we consistently recovered a diversification of crown Afrophidia (used here to refer to all alethinophidians, except Tropidophiidae + Aniliidae) near the K-Pg transition, and near-contemporaneous originations of the Amerophidia, Leptotyphlopidae, and Typhlopoidea crown groups, irrespective of the statistical framework, molecular clock model, priors, or phylogeny used (Fig. 1 and Supplementary Figs. 4–16). Implementing alternative assumptions regarding the quality of the fossil record by manipulating parameterizations of priors on fossil calibrations influenced mean estimated ages, but confidence intervals for nodes of interest—in particular for crown Afrophidia—converged near the K-Pg boundary. Choice of calibration priors influenced our estimated divergence times, reflecting the differential approaches to weighting fossil calibration ages inherent in the shapes of their probability distributions: skew-T yielded the youngest mean age estimates as it places the bulk of the effective prior distributions near the age of a fossil calibration, whereas uniform priors recovered ages averaging ~6.5 Ma older. Repeating divergence time analyses with different backbone topologies had little effect on our results (Supplementary Fig. S10, 11). The major differences between the results presented here and those of previous studies therefore appear to be a function of alternative calibration strategies (see Supplementary Information 2 for further discussion of our calibration approach), where the inclusion or exclusion of various Cretaceous fossils as internal calibrations for crown snakes influenced inferences on the timing of crown snake diversification with respect to the end-Cretaceous mass extinction. The conservative calibration approach we propose in Supplementary Information 2.2 excludes all putative records of Cretaceous crown snakes and we note that inferences on the role of the K-Pg transition in structuring the diversification of crown snakes discussed herein are dependent on this calibration scheme (see Supplementary Figs. 12–21 for exploration of alternative calibration strategies)—emphasizing the need to closely re-evaluate the phylogenetic placement of these fossils.

### Disparity through time

**Fig. 2:**
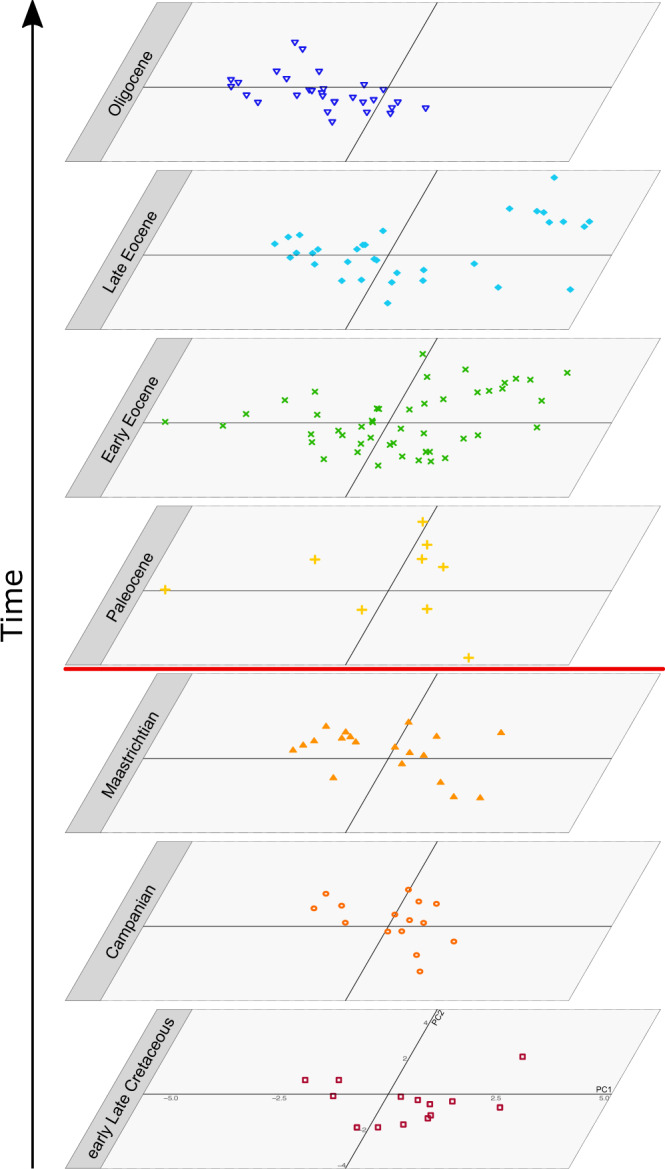
Principal component analysis of vertebral shape across different time bins, based on nine measurements, corrected for size. The K-Pg boundary is indicated by a red line.

Our analyses consider overall vertebral size independently from disparity of form, although we note that shape variables may scale allometrically. Shape disparity remained relatively constant throughout the Late Cretaceous, illustrating substantial morphological diversity early in the evolutionary history of total-clade snakes (Fig. 2 and see Supplementary Figs. 22–24 for additional axes, Supplementary Fig. 25 for labelled taxa). Disparity (sums of ranges and variances) increased across the K-Pg boundary, from the Maastrichtian to the Paleocene. Rarefaction analysis suggests that these differences were significant (the disparity of the smaller, Paleocene sample lies above the 95% confidence interval of the larger, Maastrichtian sample) and not a function of sample size differences (Supplementary Fig. 26). Higher Paleocene disparity was partly a function of a small number of morphologically eccentric taxa, notably *Titanoboa*^40^, an early palaeophiid^41^, and an indeterminate scolecophidian^42^. We note that the latter two records are late Paleocene to earliest Eocene in age—much of the apparent increase in vertebral disparity therefore appears to have been achieved after the first stage of the Paleocene. Sparse fossil sampling in the Danian means that the effects of the immediate aftermath of the K-Pg event are difficult to determine (although the very sparseness of the Danian record may point towards a decline in abundance following the extinction event^43^) and our approach, which necessarily involves time averaging and binning, is such that a short-lived crash in disparity following the K-Pg would be undetectable even if it were present. Moreover, empirical^44^ and simulation studies^45^ demonstrate that levels of disparity can be maintained in the face of even severe diversity loss, provided extinction is not centrifugal (i.e., preferentially concentrated away from the centre of morphospace).

Vertebral morphological innovations in the Paleogene were driven by adaptation to marine life by palaeophiids, including extreme dorsoventral vertebral elongation. By the late Eocene, palaeophiids form a cluster distinct from all other snakes (Fig. 2). Rarefaction analyses controlling for sample size differences reveal that the Oligocene fauna was significantly less disparate than that of the late Eocene using sum of range and sum of variance (intrinsically less susceptible to sample size differences) disparity indices (Supplementary Fig. 26).

Postzygapophyseal width has been used to estimate body length of *Titanoboa* based on regression from a sample of boine snakes^40^. Using prezygapophyseal width as a proxy for body size (similar to postzygapophyseal width but allowing for a greater sample size and also strongly correlated with the mean of all measurements, as seen in Supplementary Fig. 29), we document a significant overall decrease in size from the mid-Cretaceous to the Campanian (Supplementary Fig. 28 and Supplementary Table 4). There was no significant change over the K-Pg boundary, although the Paleocene contains some particularly large taxa such as *Titanoboa*^40^ and *Gigantophis*^46^. In the late Eocene, the increasing dorsoventral vertebral elongation and overall size increase among Palaeophiidae results in a significant difference in size between larger aquatic taxa and smaller terrestrial taxa (see Supplementary Fig. 28 and Supplementary Table 4).

### Biogeographical reconstruction

**Fig. 3:**
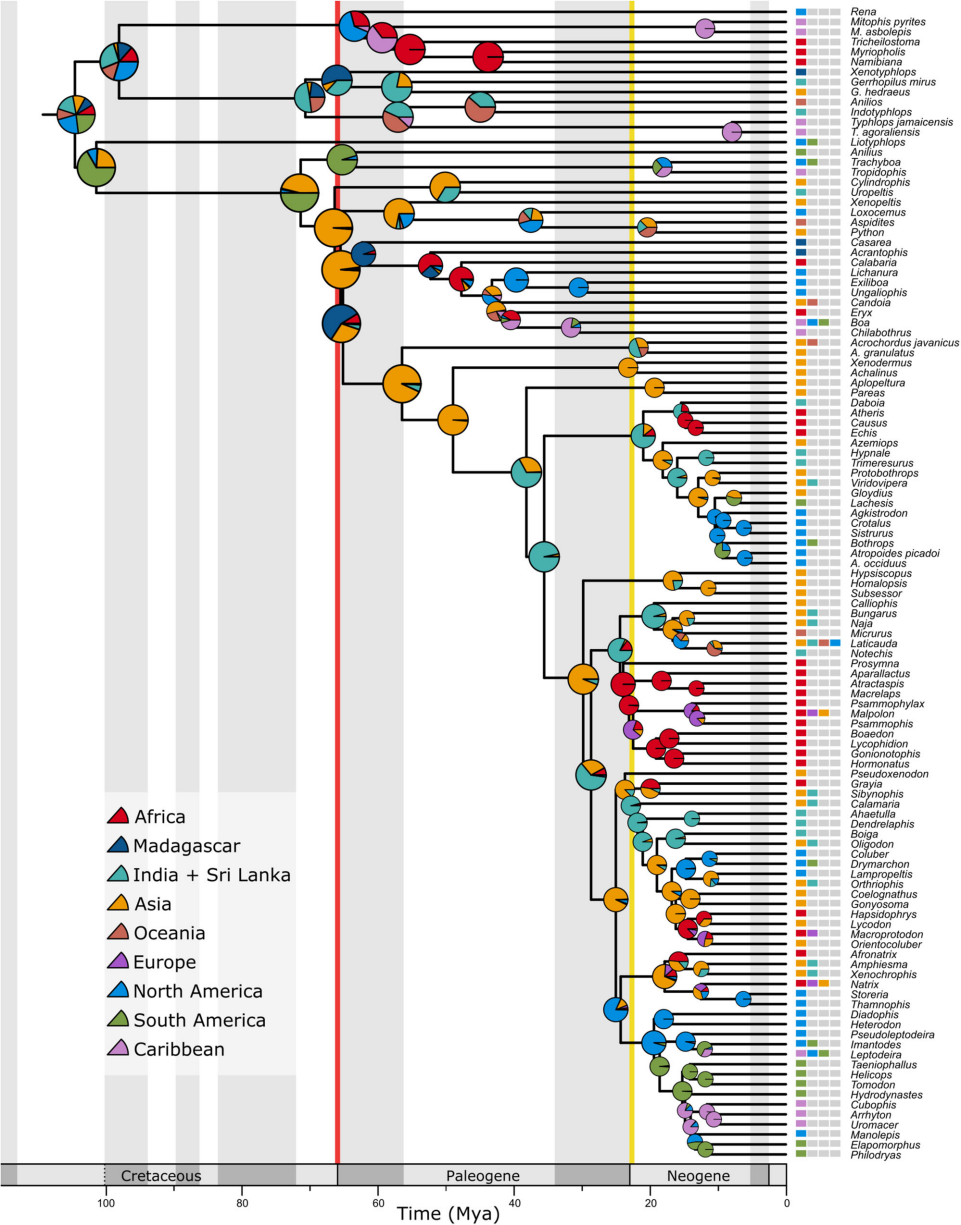
Reconstructed historical biogeography of crown snakes, applying the DIVAlike + *J* model from BioGeoBEARS. Pies represent the likelihood of the presence in each geographic area for each node; squares indicate present-day geographic distributions of extant taxa. The K-Pg boundary is indicated by a red line and the Oligocene-Miocene boundary by a yellow line.

Of six biogeographic models tested in BioGeoBEARS^47^, *+J* models (allowing founder events) consistently outperformed their simpler counterparts, with DIVALIKE *+* *J* exhibiting the best fit to our data (Supplementary Information 4.3 and Supplementary Table 6). This result suggests that crown snake biogeographic history has largely been driven by dispersal, extinction, vicariance, and especially founder events, rather than widespread or subset sympatry (DEC and BAYAREAlike models)^48^. We recovered a strong signal for an Asian origin of the major clade Afrophidia (Fig. 3); our preferred model also recovered a South American origin for Amerophidia and suggested African or North American origins for Leptotyphlopidae and Indian origins for Typhlopoidea.

## Discussion

### Early snake evolution

Cretaceous snake fossils are predominantly, but not exclusively, found in Gondwanan deposits, in line with previous analyses, which suggest a Gondwanan origin for crown snakes^34^. Diverse assemblages have been noted from as early as the Cenomanian, such as the Moroccan Kem Kem beds, which include both aquatic and terrestrial taxa^49–51^. This is also reflected in morphological disparity, with early Late Cretaceous disparity rivalling that achieved later in the Cretaceous (Fig. 2).

Our molecular clock inferences suggest that as few as six extant lineages crossed the K-Pg boundary (Fig. 1 and Supplementary Fig. 4). However, deviations from our preferred calibration scheme find different results (see Supplementary Information 2) and the fossil record documents additional extinct clades (Nigerophiidae^52^, Madtsoiidae^53^, and Coniophiidae^54^), which also passed through the end-Cretaceous extinction event. The effects of the K-Pg mass extinction on snake disparity are unclear, largely because our data do not permit fine temporal resolution. We recover higher disparity estimates in the Paleocene than in the latest Cretaceous, although several scenarios could explain this pattern. First, it is consistent with the persistence of a wide range of morphologies (and presumably ecological niches) across the boundary, indicating that, overall, the disparity of snake vertebral morphology may not have been dramatically affected by the end-Cretaceous mass extinction. Fossorial adaptations of ‘Scolecophidia’ and early branching alethinophidians may have been key to their survival, as burrowing would have provided protection against many environmental disturbances caused by the asteroid impact, including significant and global shifts in temperature^5^. Similarly, freshwater ecosystems may have been buffered against extinction through the high thermal inertia of water during an initial thermal pulse^4,5,55^ and subsequent cooling, along with food chains based more on detritus and less on photosynthesis^56,57^, and may have provided a refugium for surviving aquatic nigerophiids. Surviving alethinophidians, madtsoiids, and coniophiids were probably macropredators^34^, but snake specializations for infrequent feeding^58,59^ may have facilitated their survival despite the limited availability of large prey in the aftermath of the asteroid impact^60^. The ability of many snakes to hunt in darkness may also have aided their search for food in the low light conditions that prevailed following the impact^4^. The eventual recovery of global forests could have provided new opportunities for snakes, as it did for birds^61^ and possibly frogs^20^, with the earliest known putatively arboreal snake, *Corallus priscus*, appearing in the Early Eocene^62^.

Alternatively, snakes could have experienced a more severe but short-lived reduction in disparity across the K-Pg boundary, with regions of morphospace temporarily vacated and subsequently re-occupied by the end of the Danian. Unfortunately, the rarity of early Paleocene snakes makes it impossible to test this hypothesis at present. Additional fossils from this key interval will be needed to better understand patterns of morphospace change immediately above and below the K-Pg boundary.

### Post-extinction diversification

Fossil calibrations and their treatment have frequently been shown to have a profound effect on divergence time estimation^63,64^ and have therefore been a focus for improving molecular clock analyses^65^. Problematically, the snake fossil record is beset by a number of issues that complicate the development of unambiguous calibration schemes, relating primarily to the patchy and fragmentary nature of the snake fossil record. This has led to a number of disagreements regarding, e.g., the phylogenetic placement of fossils across the snake tree of life^33,34,37,38^; the rarity of distinctive, unambiguous synapomorphies in vertebral specimens^49^; and the absence of a comprehensive morphological phylogenetic framework with which to evaluate the affinities of problematic fossils^66,67^. These effects underlie differences in calibration choices between the scheme developed in this study and previously published calibration schemes^37,38^, such as the decision to remove Simoliophiidae and *Australophis* as calibrations due to their controversial phylogenetic placement (see Supplementary Information 2.1 for full discussion and Supplementary Figs. 4–21 to see exploration of different calibration approaches).

Alternative treatment of statistical probability distributions on fossil calibrations and chosen relaxed clock models similarly affect recovered divergence time estimates. We concatenated timetree results from two analyses applying different interpretations of the quality of the snake fossil record—using skew-T and skew-Normal priors on the fossil calibrations (see Fig. 1 and Supplementary Figs. 4 and 5). Autocorrelated clock models, in which related species are assumed to evolve at similar rates, recover younger estimated divergence times than uncorrelated clock models, where related lineages are not assumed to have similar rates of evolution. We favour the use of autocorrelated clock models on the basis that evidence for rate correlation can be detected in empirical datasets^68^ (see Supplementary Information 2.4 for full discussion).

Our inferred timescale for the evolution of crown Serpentes (following our conservative calibration scheme; see Supplementary Information 2.2) suggests a diversification of snakes spanning the K-Pg mass extinction. However, discerning the precise timing of divergence events near geologically instantaneous events such as the Chicxulub asteroid impact is challenging^16,19^. On both logical and theoretical grounds^69^, we consider it more likely for a diversification event to have occurred post-extinction, as this scenario does not imply the survival of numerous recently evolved and closely related taxa across one of the most severe mass extinction events in Earth’s history. Moreover, post-extinction fragmentation of populations and reduction in competition for resources could all facilitate a rapid diversification, as has been posited for numerous diverse vertebrate clades, such as placental mammals^15,16^, neoavian birds^18^, and acanthomorph fishes^21,22^. We therefore interpret our results regarding the timing of divergence events in Afrophidia to be consistent with a post-extinction burst in diversification.

The aftermath of the extinction event may have provided an ecological release for snakes. Following an extinction, survivors can either reoccupy vacated niche space or expand into hitherto unoccupied regions of ecospace and morphospace (to the extent that the two are correlated^44,70^) made available by the extinction of competitors and predators. A pattern of post-K-Pg morphospace expansion is supported by our disparity analysis, in which snakes realize several new and disparate vertebral morphologies in the Paleogene (Fig. 2). Our analyses also highlight the increasing specialization of marine palaeophiids, which ultimately become morphologically distinct from all contemporaneous snakes by the late Eocene. In snakes, vertebral morphology can be indicative of ecological niche and overall body size^40,71^. The extinction of non-avian dinosaurs^6^ and other terrestrial predators may have enabled snakes to exploit relatively abundant small vertebrate prey in the early Cenozoic. Similarly, the K-Pg-associated extinction of marine reptiles and large teleosts may have facilitated the exploitation of marine ecosystems by large Paleocene marine snakes (Supplementary Fig. 28).

Our results suggest that the K-Pg mass extinction also influenced biogeographic patterns among snakes. We infer an initial dispersal by snakes into Asia near the K-Pg boundary (a landmass that they had apparently not previously occupied, see Supplementary Information 4.1), coinciding with the diversification of crown Afrophidia (Fig. 3). These patterns are consistent with the hypothesis that extinction events may help drive biogeographic patterns^30^. Although the probability of intercontinental dispersal should be unaffected by a mass extinction event, the extinction of potential competitors and predators may increase the likelihood that dispersed lineages ultimately become established on a newly colonized landmass.

### Oligocene extinction and recovery

Significantly, our results indicate a second major snake extinction and recovery event in the Oligocene. We infer multiple dispersal and diversification events within caenophidian snakes beginning near the Oligocene-Miocene boundary and extending into the early Miocene (Figs. 1 and 3). We also infer a significant drop in morphospace occupation from the late Eocene to the Oligocene, supported further by the disappearance of early Cenozoic marine snakes such as Palaeophiidae and *Gigantophis* from the fossil record^72^ (Fig. 2). The Eocene-Oligocene event was associated with severe global cooling^73^ and may therefore have particularly affected poikilothermic taxa such as snakes. We hypothesize that caenophidians may have acquired their predominantly diurnal habits during this cooler interval as a result of being driven towards elevated daytime activity levels to take advantage of warmer daytime temperatures. Niche differentiation from their nocturnal antecedents may therefore have played a primary role underpinning caenophidian radiation and post-dispersal colonization during this interval^34,74^. Warming in the late Oligocene would have permitted the occupation of higher latitudes by poikilothermic snakes, facilitating their dispersal to the Americas via Beringia, as has been posited for birds^75^ (Fig. 3). Concurrent fragmentation of forests and the spread of newly appearing grasslands^76^ may also have provided opportunities for speciation and adaptation to changing environments (Fig. 1).

It is clear that integrating molecular sequence data and the fossil record can elucidate the influence of major events in Earth’s history on the evolutionary history of extant clades^19^. Our analyses suggest that the K-Pg mass extinction influenced the evolutionary history of snakes, potentially facilitating the diversification of Afrophidia and the origination of several other snake subclades in the extinction’s aftermath. This appears to have enabled the successful establishment of snakes in Asia and provided the opportunity for Palaeophiidae to specialize as marine predators in the Eocene. Along with the contemporaneous diversification and disparification of numerous other vertebrate clades^16–22^, our results help corroborate the fundamental role of the K-Pg mass extinction in shaping the vertebrate biodiversity occupying our planet today.

## Methods

### Phylogenetic analyses

Analyses included 115 extant snake taxa. Forty-four non-snake squamates across all major squamate clades were sampled as outgroups and to provide additional calibration points. Ten non-squamate amniote outgroups were included to calibrate deeper nodes (see Supplementary Fig. 3). Taxa were sampled from the Zheng and Wiens^32,77^ supermatrix. Criteria for inclusion were maximizing representation of extant families, minimizing missing data within the phylogenetic data matrix, and prioritizing slower evolving taxa. All extant snake families, except Anomochilidae and Xenophidiidae, were included, as their sparse gene sampling reduced resolution.

We extracted molecular sequence alignments for our chosen taxa from the Zheng and Wiens^32^ supermatrix, which includes 40 nuclear and 12 mitochondrial loci. Overall matrix completeness was 49.7%. We ran phylogenetic analyses in PhyloBayes v.4.1c^78^ for ~30,000 generations, under the CAT + GTR + G parameters and with a birth–death prior, as PhyloBayes does not co-estimate tree topology and divergence time, and is MPI (Message Passing Interface) enabled^79^. We built a consensus tree, with a burn-in of 7500 generations and retaining every tenth tree. For analyses where different tree topologies were enforced, we used Mesquite v. 3.10^80^ to manipulate relationships in the consensus tree, to set ‘Scolecophidia’ as monophyletic, and to change immediate outgroup to be Iguania + Anguimorpha, as in Reeder et al.^81^.

### Calibrations

We compiled a set of 42 calibrations, comprising previously published and novel calibrations (see Supplementary Information 2.2). Multiple variations of this calibration set were also tested, including the addition of Simoliophiidae as a minimum calibration for Alethinophidia^32–34,38^, as well as testing parviraptorids as a calibration for total-group snakes (see Supplementary Table 3 for summary). In addition, the calibration schemes proposed by Head^38^ and Head et al.^37^ were also tested, as well as a revision of this set with two amendments: the removal of Simoliophiidae to assess the effect of assigning them to crown Alethinophidia and the removal of *Australophis* (see Supplementary Information 2.5).

### Estimating divergence times

To investigate the effects of different probabilistic methods, different relaxed clock models and different prior distributions of our fossil calibrations on divergence time estimates, we performed analyses in the PAML package MCMCTree^82^. For these analyses, data were partitioned by gene^83,84^. Prior distribution shapes and scales for calibrations were calculated using the MCMCTreeR package^85^. We used soft maximum bounds, with a tail of 0.05, and a root prior for Amniota of 318–332.9 Ma^86^. Divergence time estimates were calculated under Skew-T, Skew-Normal, and Uniform distributions, to simulate progressively more liberal interpretations of the fossil record^83^. Further analyses were run using the uniform and/or skew-T prior distribution with both independent rates and correlated rates, alternative topologies, and with alternative calibration schemes (see Supplementary Figs. 4–21). Analyses performed under an agnostic distribution (the uniform prior) are important. These make the fewest assumptions regarding the quality of the fossil record and are modelled with a hard minimum age. If our preferred set of assumptions were to be found invalid, uniform priors help to circumscribe the range of inferences that might reasonably be made from our data. However, given the fossil record of snakes significantly improved in the Cenozoic, where most of our calibrations are found, there is no reason to prefer an agnostic distribution over other priors; for Fig. 1, results from skew-T and skew-Normal priors were concatenated. Divergence time analyses were run for 100,000 generations, with a burn-in of 25,000 and sampling every 25th remaining tree.

### Biogeographical reconstruction

Historical biogeography was reconstructed using BioGeoBEARS^47^. Geographical data were collected for ingroup taxa from the Reptile Database^87^. Full methods can be found in Supplementary Information 1 and input files in Supplementary Information 4.1-3. We assessed model fit using log likelihood, AIC (Akaike Information Criterion), and AICc (AIC with correction for small data samples) values.

### Disparity analyses

Nine linear measurements were taken between geometrically homologous landmarks on fossil snake vertebrae using the software ImageJ^88^. As some vertebrae were incomplete, missing values were inferred using multiple imputation. For this we used the *missRanger*^89^ package in R, which implements the *MissForest* chaining random forests algorithm of Stekhoven and Bühlmann^90^ with predictive mean matching and a maximum of 50 chaining iterations. To control for differences in specimen size, all measurements were scaled to the mean of all measurements for said specimen. The data were then subjected to R-mode principal component analyses using a correlation matrix. All resultant PC axes were used to calculate sums of ranges and sums of variances as indices of disparity. In order to account for the effects of sample size variation, we jackknifed both indices of disparity at all possible sample sizes using *Rare*^91^ to *n* = 9 (smallest time bin). Rarefied indices of disparity were then plotted against logged sample size. Changes in size through time were independently assessed in R^92^, based on the raw prezygapophyseal width data. Kruskal–Wallis tests were used to test for significant changes in size between time bins and between inferred ecologies in the same time bin. For specimens with age uncertainty, entries were duplicated so as to be present in both relevant time bins. Overall, resultant sample sizes were *n* *=* 14 for the early Late Cretaceous, *n* *=* 15 for the Campanian, *n* *=* 19 for the Maastrichtian, *n* *=* 9 for the Paleocene, *n* *=* 48 for the Early Eocene, *n* *=* 33 for the late Eocene, and *n* *=* 30 for the Oligocene.

### Reporting summary

Further information on research design is available in the Nature Research Reporting Summary linked to this article.

## Supplementary information

Supplementary Information

Reporting Summary

## Data Availability

Input and output files for molecular clock and disparity analyses are deposited at 10.5061/dryad.tv055. Information on extant taxon ranges was gathered from the Reptile Database https://reptile-database.reptarium.cz/. Gene sequences from Zheng and Wiens^32,77^ can be found at 10.5061/dryad.tv055. All other data can be found in the Supplementary Information. Different formats can be requested from corresponding authors.
