# Supplementary material for: Evolution and dispersal of snakes across the Cretaceous-Paleogene mass extinction
Source: Nat Commun. 2021 Sep 14;12:5335. doi: 10.1038/s41467-021-25136-y (PMC8440539; doi:10.1038/s41467-021-25136-y)
Supplement: Supplementary file 1 — Supplementary Information [file 41467_2021_25136_MOESM1_ESM.pdf]

Supplementary Information – Evolution and dispersal of snakes across the Cretaceous-Paleogene mass extinction

Table of Contents

SUPPLEMENTARY INFORMATION 1: EXTENDED METHODS 3

Phylogenetic analyses **3**
Supplementary Table 1 3
Supplementary Table 2 4

Calibrations **5**

Estimating divergence times **5**

Disparity analyses **6**
Measurements 7
Principal component analyses (PCA) 9
Size through time 11

Biogeographical reconstruction **11**

SUPPLEMENTARY INFORMATION 2: MOLECULAR CLOCKS 13

2.1 Rationale for choice of calibration points **13**

2.2 Calibration justifications **17**

2.3 Notes on excluded calibrations **31**
Calibrating the snake stem lineage 31
Simoliophiidae as crown Alethinophidia 32
Coniophis as crown Alethinophidia 32
Using geological events to inform new analyses 33
The exclusion of the Vellberg jaws 33

2.4 Rationale for choice of clock model **35**

2.5. Additional output **39**
Supplementary Figure 3 39
Supplementary Table 3. 40
Supplementary Figure 4 41
Supplementary Figure 5 42
Supplementary Figure 6 43
Supplementary Figure 7 44
Supplementary Figure 8 45
Supplementary Figure 9 46
Supplementary Figure 10. 47
Supplementary Figure 11 48
Supplementary Figure 12 49
Supplementary Figure 13 50
Supplementary Figure 14 51
Supplementary Figure 15 52
Supplementary Figure 16 53
Supplementary Figure 17. 54

Supplementary Figure 18. 55
Supplementary Figure 19. 56
Supplementary Figure 20. 57
Supplementary Figure 21. 58

SUPPLEMENTARY INFORMATION 3: DISPARITY AND SIZE THROUGH TIME 59

3.1 Principal Component Analysis **59**
Supplementary Figure 22. 59
Supplementary Figure 23. 60
Supplementary Figure 24. 61
Supplementary Figure 25. 62
Supplementary Figure 27. 65

3.2 Size through time analysis **66**
Supplementary Figure 28. 66
Supplementary Figure 29. 67
Supplementary Table 4. 67

SUPPLEMENTARY INFORMATION 4: BIOGEOGRAPHY 69

4.1 Dispersal multipliers **69**
Supplementary Table 5. 69
Areas allowed 70

4.2 Statistical results **70**
Supplementary Table 6. 70

SUPPLEMENTARY REFERENCES 71

Supplementary Information 1: Extended methods

Phylogenetic analyses

Our analyses included 169 extant taxa, of which 10 were non-squamate amniote outgroups (see supplement). 44 non-snake squamate taxa, sampling all major clades of extant squamates, were also included to serve as outgroups and provide additional calibration points. 115 extant snake taxa were included. The rationale for the inclusion/exclusion of taxa was to maximise the representation of extant families, minimise missing entries within the data matrix, and minimise branch lengths. All families except Anomochilidae and Xenophidiidae (*sensu* Zheng & Wiens[1]) were included, and the latter were excluded because of poor coverage and a consequent decrease in stability and branch support in the resulting tree.

Supplementary Table 1. List of all outgroup taxa used in the analysis.

Acontias breviceps
Agama agama
Alligator mississippiensis
Amphisbaena fuliginosa
Anelytropsis papillosus
Anniella pulchra
Anolis carolinensis
Basiliscus basiliscus
Bipes biporus
Callopistes maculatus
Chamaeleo calyptratus
Chelydra serpentina
Conolophus pallidus
Conolophus subcristatus
Cordylosaurus subtessellatus
Crocodylus porosus
Dibamus greeri
Dibamus montanus
Dibamus novaeguineae
Dromaius novaehollandiae
Elgaria multicarinata
Enyalioides laticeps
Eublepharis macularius
Gallus gallus
Gekko gecko
Geocalamus acutus
Heloderma suspectum
Homo sapiens
Lacerta viridis
Lanthanotus borneensis
Lepidophyma flavimaculatum
Lialis burtonis
Microlophus albemarlensis
Microlophus delanonis
Mus musculus
Pholidobolus macbrydei
Podocnemis expansa
Pseudopus apodus
Rhineura floridana
Scincus scincus
Shinisaurus crocodilurus
Smaug mossambicus
Sphenodon punctatus
Strophurus ciliaris
Tachyglossus aculeatus
Tiliqua scincoides
Trachylepis quinquetaeniata
Tupinambis teguixin
Uromastyx aegyptia
Varanus acanthurus
Varanus salvator
Xantusia vigilis
Xenosaurus grandis
Zonosaurus ornatus

Supplementary Table 2. List of all ingroup taxa used in the analysis

Achalinus meiguensis
Acrantophis madagascariensis
Acrochordus granulatus
Acrochordus javanicus
Afronatrix anoscopus
Agkistrodon contortrix
Ahaetulla pulverulenta
Amphiesma stolatum
Anilios australis
Anilius scytale
Aparallactus werneri
Aplopeltura boa
Arrhyton supernum
Aspidites melanocephalus
Atheris squamigera
Atractaspis irregularis
Atropoides occiduus
Atropoides picadoi
Azemiops feae
Boa constrictor
Boaedon fuliginosus
Boiga barnesii
Bothrops asper
Bungarus fasciatus
Calabaria reinhardtii
Calamaria pavimentata
Calliophis melanurus
Candoia carinata
Casarea dussumieri
Causus defilippii
Chilabothrus striatus
Coelognathus flavolineatus
Coluber constrictor
Crotalus viridis
Cubophis cantherigerus
Cylindrophis ruffus
Daboia russelii
Dendrelaphis bifrenalis
Diadophis punctatus
Drymarchon corais
Echis ocellatus
Elapomorphus quinquelineatus
Eryx colubrinus
Exiliboa placata
Gerrhopilus hedraeus
Gerrhopilus mirus
Gloydius halys
Gonionotophis capensis
Gonyosoma oxycephalum
Grayia ornata
Hapsidophrys smaragdina
Helicops angulatus
Heterodon platirhinos
Homalopsis buccata
Hormonotus modestus
Hydrodynastes bicinctus
Hypnale nepa
Hypsiscopus plumbea
Imantodes cenchoa
Indotyphlops braminus
Lachesis muta
Lampropeltis getula
Laticauda colubrina
Leptodeira annulata
Lichanura trivirgata
Liotyphlops albirostris
Loxocemus bicolor
Lycodon rufozonatus
Lycophidion capense
Macrelaps microlepidotus
Macroprotodon cucullatus
Malpolon monspessulanus
Manolepis putnami
Micrurus fulvius
Mitophis asbolepis
Mitophis pyrites
Myriopholis boueti
Naja naja
Namibiana occidentalis
Natrix natrix
Notechis scutatus
Oligodon taeniolatus
Orientocoluber spinalis
Orthriophis taeniurus
Pareas hamptoni
Philodryas olfersii
Prosymna janii
Protobothrops mangshanensis
Psammophis sibilans
Psammophylax variabilis
Pseudoleptodeira latifasciata
Pseudoxenodon bambusicola
Python molurus
Rena humilis
Sibynophis collaris
Sistrurus catenatus
Storeria dekayi
Subsessor bocourti
Taeniophallus affinis
Thamnophis marcianus
Tomodon dorsatum
Trachyboa boulengeri
Tricheilostoma bicolor
Trimeresurus trigonocephalus
Tropidophis haetianus
Typhlops agoralionis
Typhlops jamaicensis
Ungaliophis continentalis
Uromacer oxyrhynchus
Uropeltis melanogaster
Viridovipera stejnegeri
Xenochrophis piscator
Xenodermus javanicus
Xenopeltis unicolor
Xenotyphlops grandidier

Molecular sequence alignments for the chosen taxa were extracted from the supermatrix of Zheng & Wiens[1,2], which includes 40 nuclear and 12 mitochondrial loci. Matrix completeness was 49.7%. Phylogenetic analyses were run in PhyloBayes v.4.1c [3] for ~ 30,000 generations, under a CAT+GTR+G model, and with a birth-death prior. Three chains were run, and convergence was assessed using the bpcomp function in PhyloBayes, with the aim of having maximum discrepancy values below 0.3 and minimum effective size above 50. The two chains with the greatest convergence were used to build a consensus tree, with a burn-in of 7,500 generations and every tenth tree being retained. For subsequent analyses where different tree topologies were enforced we used Mesquite v. 3.10 to move branches [4].

Calibrations

A set of 42 calibrations was assembled, comprising previously published and novel calibrations (see Supplementary Information SI2). These consisted of 8 nodes calibrated with both maximum and minimum ages, 5 with maximum ages only, and 30 with minimum ages only. The calibration scheme focused on establishing relatively consistent calibration coverage across phylogenetic diversity and through time, drawing from relatively well-understood deeper divergence events at the base of the tree, and more recent nodes representing endemic island radiations. We also took a conservative approach to fossil minima, focusing on fossils that are unequivocally assigned to clades and excluding fossils whose referrals to extant clades, in the opinion of our fossil snake specialist co-author (NRL), were uncertain.

A variation of this calibration set was also tested, by adding Simoliophiidae as a minimum age calibration for Alethinophidia [1,5]. The calibrations proposed by Head[5] and Head et al.[6] were tested, as were amendments of this calibration set (removing Simoliophiidae and/or *Australophis* as a minimum calibration of Alethinophidia) to assess the effect of certain phylogenetically uncertain fossils on divergence time estimates (see SI2).

Estimating divergence times

To investigate the divergence time effects of different probabilistic methods, different relaxed clock models, and different prior distributions on the fossil calibrations, analyses were performed in PAML [7]. Branch lengths were calculated using the PAML package BASEML, and divergence times were approximated using the PAML package MCMCTree[7]. MCMCTree has a comprehensive

selection of models of rate variation, but was primarily chosen for its fast approximate likelihood method[7,8] (Yang2007, dos Reis 2015). Parameters were amended to mirror analyses run in PhyloBayes, and data were partitioned by gene. Prior distribution shapes and calibration scales were calculated using the MCMCTreeR code by Puttick[9]. Divergence time estimates were calculated under Skew-T, Skew-Normal, and Uniform distributions, so as to test alternative interpretations of the fossil record's quality. As the calibration set consists of some nodes with only minimum or maximum ages specified, the opposite age was estimated by adding or subtracting 200 My from the 'known' age. This arbitrary number was chosen as it creates a wide-enough band for internal truncation of node distributions to act; a node cannot, for example, be older than its ancestral node, and any calibration information given to the ancestral node thus limits the potential maximum age of the initial node. These analyses were run with both independent rates and correlated rates. Divergence time analyses were run for 100,000 generations, with a burn-in of 25,000 and sampling every 8th remaining tree. Consensus trees of multiple analyses were computed by compiling results from analyses, then drawn using the MCMCTreeR package.

Analyses looking into the effects of alternative calibration strategies, parameter choices, clock models, and partition schemes were run under a Uniform or a Skew-T prior. One weakness of the Skew-T prior as applied here is that it can allow the violation of minimum calibrations, as these are not hard bounds, even if younger ages are given extremely low probabilities (see Fig. S13, where the age of total-group snakes is recovered as younger than the calibration at 164.9 Mya). While this could allow the "washing out" of erroneous calibrations at odds with other calibrations, this also means that the effect of different calibration schemes on recovered age estimates cannot be clearly tested. While it is our belief that the snake fossil record is not so poor as to suggest the use of a relatively uninformative prior such as the Uniform prior as necessary, as reflected in the use of concatenated results using Skew-T and Skew-Normal priors for Figure 1, it is therefore nonetheless informative to run additional analyses under the Uniform prior.

Disparity analyses

In order to quantify the morphological diversity of snakes, whilst accounting for the paucity of fossilised material, we used linear measurements from mid-trunk vertebrae. Vertebrae are the most readily fossilised bones in snakes, both because of their number and their greater robustness in comparison with slender elements of the skull. Measurements were taken in order to capture

the variation in morphology of snakes occupying different ecological niches. As the main locomotor module, snake trunks reflect niche occupation; fossorial vertebrae show highly reduced features and are generally cube in shape, whereas aquatic vertebrae may showcase lateral compression, long neural spines, or be pachyostotic. Lawing et al.[10] showcased the relationship of both mean vertebral width-to-length ratio and vertebral shape (landmarked on anterior views) to substrate use. Additional measurements used here include interzygapophyseal constriction and hypapophysis height. The former relates to aquatic habits observed in Simoliophiidae, as vertebral shape is 'filled out' through pachyostosis, presumably in order to aid in sinking (eg. visible in [11,12] compared to [13]).

Measurements

Measurement 1 (Fig. S1A):

Prezygapophyseal width – taken as the distance between the lateralmost extents of the articular facets of the prezygapophyseal articular facets. Prezygapophyseal processes were not included in width measurements. Measurements were taken primarily on anterior views of vertebrae, but dorsal views were used in the absence of anterior views.

Width at the zygapophyses has previously been used as a proxy for total length[14], and was used here to scale all subsequent measurements for the principal component analyses.

Measurement 2 (Fig. S1B):

Interzygapophyseal width – taken as the width of the vertebra at the narrowest point between the prezygapophyses and postzygapophyses.

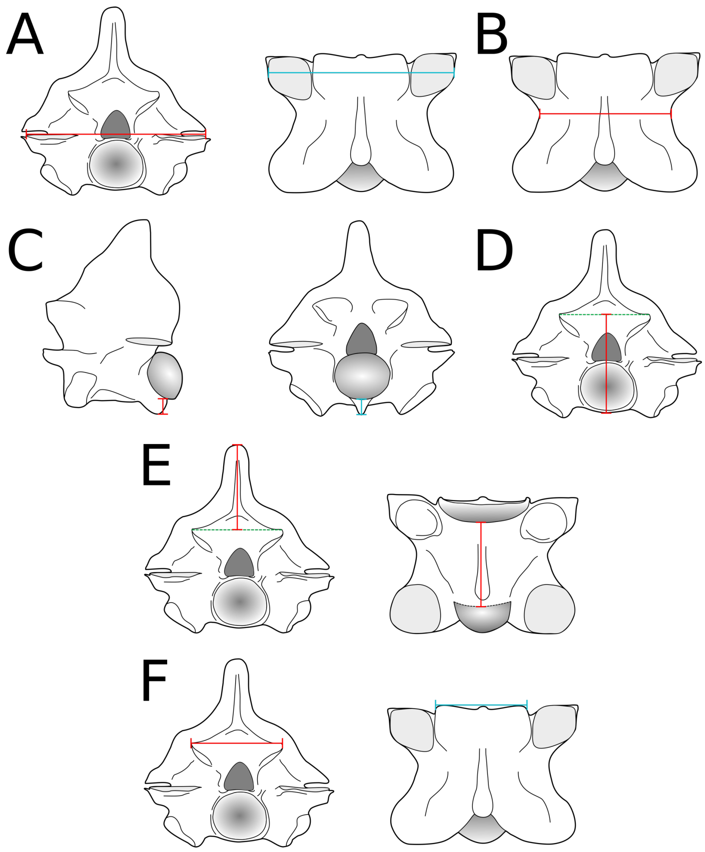

Supplementary Figure 1. Indication of how data for measurements 1 to 6 were taken, with preferred methods in red and alternative methods in blue.

Measurement 3 (Fig. S1C):

Hypapophysis height – taken as the vertical distance from the distalmost extent of the hypapophysis or neural keel, to the ventral extent of the centrum where the condyle is attached. Measurement primarily taken in lateral view as the condyle can distally project ventrally, but measurements were taken in posterior view as an alternative source of data.

Measurement 4 (Fig S1D):

Height – taken as the distance between the ventral aspect of the cotylar lip and the midpoint between the dorsal extent of the lateral zygosphene articular surfaces. Measurements were taken on anterior views. The dorsal extent of the zygosphene roof was not used, as it can be vaulted in taxa such as *Pterosphenus*.

Measurement 5 (Fig. S1E):

Centrum length – taken as the distance from the median point of the cotylar lip in ventral view, to the posteriormost point before the start of the condyle. The condyle was not included in measurments to allow the inclusion of fossils in which it is incompletely preserved.

Measurement 6 (Fig. S1F):

Zygosphene width – taken as the distance between the lateral extents of the ventrolateral articular surfaces of the zygosphene. Measurements primarily taken on anterior views but could be taken in dorsal view as an alternative. In dorsal view, measurements were taken at the anterior border of the zygosphene, as they can expand posteriorly.

Measurements 7 and 8 (Fig. S2A,B):

Condyle width and height – taken as the maximum vertical and horizontal extents of the condyle. Taken primarily on posterior views of vertebrae, but in their absence were taken on anterior views. In this case, measurements were taken inside the cotylar lip, so as to only include the articulating surfaces.

Measurements 9 and 10 (Fig. S2C,D):

Neural canal width and height – taken as the maximum vertical and horizontal extents of the neural canal. Measurements were taken primarily on posterior views of vertebrae, so as to maximise comparability of data. If the posterior view was not provided or poorly preserved, anterior views were used instead.

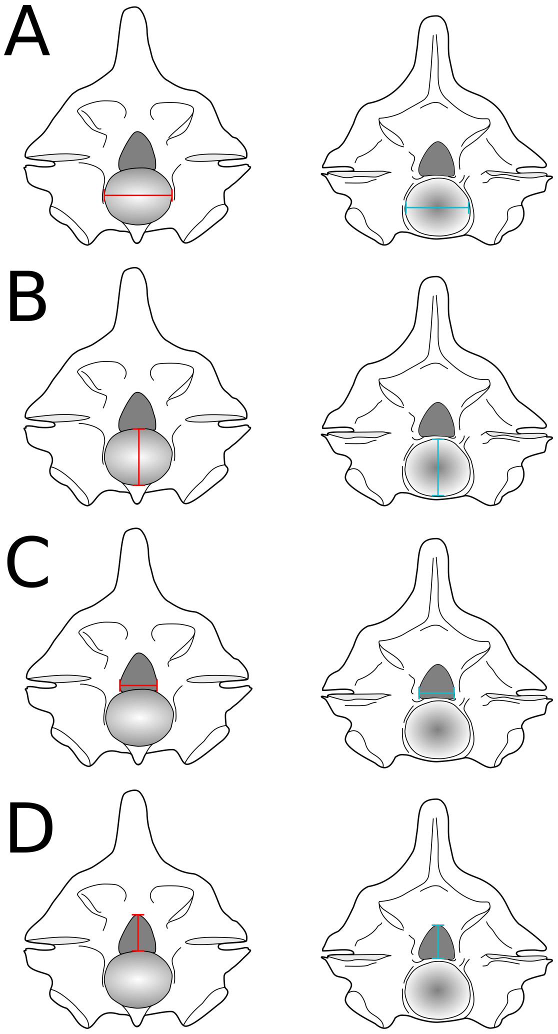

Supplementary Figure 2. Indication of how data for measurements 7 to 10 were taken, with preferred methods in red and alternative methods in blue.

Principal component analyses (PCA)

In order to better visualise patterns of morphological variance across our sample, and also to reduce the dimensionality of some subsequent analyses, we subjected our data to R-mode principal component analysis (PCA). Prior to analysis, missing values were imputed using the

missRanger[15] package in R, using 10,000 trees. All measurements were then scaled by the mean of all measurements for the corresponding vertebra. The remaining measurements were thereby a reflection of variations in shape rather than a combination of both size and shape. Many vertebrae had incurred damage to the neural spines, such that 55% of cells had no data for this variable. Hypapohysis height varies highly with position along the column, and although only 'mid-trunk' vertebrae were considered, this still covers a significant amount of intracolumnar variation. We therefore removed neural spine and hypapophysis heights from our calculations, leaving nine dimensions from which to estimate disparity. These contained far fewer missing values than the neural spine (a mean of 6.1% and maximum of 12.3%).

Where multiple specimens are available to represent a single species, there is potential for biasing the sample. As such, average measurements of taxa were calculated so that they are not over-represented in the dataset. This was not done if specimens were recovered in different time bins, vastly different geographical regions, or reflected morphologies which were significantly different in preliminary principal component analyses. This last was to overcome potential issues of taxonomic misidentification or hidden diversity. Time bins of approximately equal duration were set at 'early Late Cretaceous', 'Campanian', 'Maastrichtian', 'Palaeocene', 'Early Eocene', 'Late Eocene', 'Oligocene'. Finer time binning was not possible because our sample was not large enough. The 'early Late Cretaceous' spans from the Cenomanian to the Santonian because sampling in the Turonian, Coniacian, and Santonian is sparse.

All PCAs were computed in PAST[16], and derived from matrices of correlations between variables, thereby effectively scaling each variable to unit variance (equivalent to transforming the values for each variable into their z-scores: expressed as standard deviation units around the mean). The resulting PCA plot captured 39%, 23% and 12% of total variance on the first, second and third PC axes respectively (74% of total shape variation on the first three axes combined). We used all nine resultant PC axes to calculate two disparity metrics – sums of variances and sums of ranges – for taxa from each time bin. Variance-based indices of disparity are much less sensitive to differences in sample size than are those derived from ranges. Moreover, where all axes are incorporated, the sum of variances also has the advantage that it is insensitive to the orientation of those axes. This means that the sum of variances inferred from all of the original variable axes should scale in proportion to sum of variances from all of the PCA axes. Range-based indices, by contrast, are much more sensitive to sample size differences, which varies from 3 (aquatic taxa only) to 33 (all

sampled Oligocene taxa) across our time bins. We therefore subjected the taxa in each bin to rarefaction analysis using the script ‘Rare’ [17]. At all sample sizes between two and the number of taxa in each bin, we jack-knifed (sampling without replacement) 5,000 samples and inferred a median and 5% tails. These rarefied disparity indices have been plotted against the log of sample size (see Fig. S26) such that disparity can be compared at all possible sample sizes.

Size through time

Preliminary PCA analyses, without scaling measurements to size (i.e., expressing all as a multiple of the width of the prezygapophysis), revealed that size accounts for ~95% of total variation in the linear dimensions. It was therefore important to look at size through time in addition to the scaled analyses of shape disparity. Only images of vertebrae with scale bars were used. Prezygapophyseal width was used as a proxy for body size, as has been shown previously in boine snakes[14]. This correlates strongly with the mean measurement scaling used for PCA (see SI3 Fig. S22), suggesting they support the same signals. Data were also time binned (‘early Late Cretaceous’, ‘Campanian’, ‘Maastrichtian’, ‘Palaeocene’, ‘Early Eocene’, ‘Late Eocene’, ‘Oligocene’). As well as analysing all data together, further analyses were also run separating the snakes by inferred ecological preference (aquatic vs terrestrial). Shapiro-Wilk tests on each individual bin revealed non-normally distributed data. We therefore performed Kruskal-Wallis tests to check for significant differences between time bins.

Biogeographical reconstruction

Historical biogeography was reconstructed using the R package BioGeoBEARS[18,19]. Geographical data was collected for all ingroup taxa from the Reptile Database[20]. The taxon *Indotyphlops braminus* was replaced by its sister taxon *I. pammeces* as the global distribution of *I. braminus* is both uninformative and causes the modelling to become too computationally intense, by forcing the maximum number of areas occupied by a single taxon from 5 to 9. Relative likelihood of dispersing between continental landmasses was assessed using palaeogeographical reconstructions[21] and given values from 0.001 (dispersal from A to B directly extremely unlikely) to 1.0 (dispersal highly likely due to physical connection of landmasses). Values were estimated for 3 time bins, to represent broad changes in palaeogeography. Input files can be found at SI4 and Table S5.

Based on log likelihood, AIC and AICc values (see SI4, Table S6), the Diva-like + J model was best able to fit the data, although it only marginally outperformed the DEC+J and Bayarea-like + J models.

Supplementary Information 2: Molecular clocks

2.1 Rationale for choice of calibration points

Molecular clock models are heavily dependent upon the calibration points used to constrain the minimum and maximum ages of phylogenetic divergences. Our approach was informed by arguments made concerning the best practices for phylogenetic analysis[22] which emphasize the use of a voucher specimen, fossils that are well-constrained by apomorphies and ideally phylogenetic analysis, and careful dating of fossils. The calibration scheme applied here was established by our fossil snake specialist co-author (NRL) and includes a number of calibrations that have previously been proposed for snakes[5,6]. However, some previously proposed calibrations were not used because they were not deemed to fulfil conservative requirements for well-chosen calibrations as discussed below. Fossils were rejected for a variety of reasons including high levels of anatomical homoplasy resulting in stemward fossil taxa being difficult to place phylogenetically, conflict between molecular and morphological phylogenetic hypotheses for snakes, and the fragmentary nature of many fossils. As a result, our dataset is more restricted than other previously proposed calibration schemes for snakes.

1. **The K-Pg boundary was not used as a maximum age, to avoid circularity.** A number of groups of animals, for example Primates, show a pattern in which stem group representatives appear soon after the K-Pg boundary, followed by members of the crown group. Such patterns appear consistent with a post-extinction radiation and may suggest that the K-Pg boundary would be an appropriate maximum bound for probability distributions associated with fossil calibration points, and it has been applied as one in some studies[23]. However, if the timing of origin with respect to the boundary is the hypothesis of interest, such an approach is inherently circular. We therefore uniformly avoided the K-Pg boundary as a maximum age constraint for any clade to allow our model to recover crown snake lineages extending back into the Cretaceous.

2. **Continental splits were not applied as divergence time minima, to avoid circularity**. A number of molecular clock studies have applied the age of continental rifting events as constraints for the minimum ages of clades[24,25], based on the assumption that taxa found on several continents may have been widespread prior to landmass

fragmentation (i.e. their widespread distribution is due to vicariance). However, basing biogeographic arguments for vicariance on the outcome of divergence time estimates that themselves assumed vicariance as a basis for the minimum age calibration of those clades is circular. As our analyses sought to illuminate the biogeographic history of crown snakes we avoided the application of such biogeographic age constraints.

3. **Extensive outgroup calibration.** To better constrain both the timing of divergence and rates of evolution for lineages deep in the phylogeny, we included and calibrated multiple outgroups including archosaurs, turtles, and birds.

   We assigned maximum ages to outgroup divergences where the timing of events is thought to be relatively well-understood, following recent work[26] (e.g., the split for a number of deeper divergences within Amniota and within Squamata such as crown Lepidosauria and crown Squamata). This approach assumes that the fossil record provides generally accurate, if not highly precise, constraints on the maximum ages of a number of the deeper divergences in crown-group amniote evolution. We assume that the fossil record provides reasonably accurate information on the maximum ages of outgroup amniote clades, but no such fossil-based assumptions were used for the ingroup under study (crown Serpentes) in order to allow for the possibility that crown snake lineages are significantly older than the K-Pg boundary.

4. **Island maxima were used to calibrate ingroup taxa.** To provide constraints on the maximum ages of ingroup clades, the ages of endemic island clades were used to calibrate the maximum age of splits within both snakes and lizards. The rationale is that if an island endemic clade originated on an island, then necessarily the clade can be no older than the island in question.

   This approach is not infallible: it could provide erroneous estimates for maximum ages in certain circumstances, including, for example, if the island's age is inaccurately estimated, or the clade of interest originated on another landmass where it is now extinct, and since dispersed to a younger island one or more times. However, the advantage of using islands to provide maxima is that islands provide an objective, repeatable, and intellectually justifiable constraint on the maximum age of clades. Using the fossil record to derive maxima, by contrast, relies on subjective assumptions

about the likelihood that a lineage has remained unsampled, or assumptions about the link between geological events and the origination of particular clades.

5. **Major geological stage boundaries are used for maxima for outgroup taxa.** Large-scale studies of the fossil record suggest that turnover is often concentrated at major boundaries, particularly the Cretaceous-Paleogene, Triassic-Jurassic, and Permo-Triassic boundaries[27]. Such major transitions are associated with elevated levels of extinction, sometimes exceeding 50%[28], as well as relatively rapid diversification events in their aftermaths[29]. It follows that adaptive radiation in the wake of extinctions means that cladogenesis is more likely in the aftermath of such events, thus the probability of a divergence above such a boundary increases[27]. Clades suddenly appearing above such boundaries (e.g., crown turtles and crown mammals in the Jurassic, or crown archosaurs in the Triassic) are therefore more likely to represent post-extinction originations (as opposed to representing multiple lineages extending back across an extinction horizon). A similar argument can be made for the mid-Cretaceous; a series of oceanic anoxic events including the mid-Aptian OAE1 and the Cenomanian-Turonian OAE2, which is associated with a minor mass extinction[29] appear to be associated with elevated levels of turnover. Accordingly, we have used 100 Ma as a maximum for several crown clades including Euarchontoglires and crown Aves. This approach was avoided for our ingroup, as discussed above.

6. **Conservative calibration choice resulted in Cretaceous snake fossils largely being excluded from our calibration scheme, due to poor phylogenetic resolution and large amounts of missing data.** Calibration choices were complicated first by the fact that the fossil record of squamates, and especially snakes, is incomplete, and that morphological homoplasy in squamates is more severe than has often been appreciated[30].

   Molecular phylogenies[1,31,32] and combined evidence analyses[30,33,34] have shown that many clades inferred on the basis of morphological data[35,36] are questionable[30]. In particular, within snakes, many traditional groupings such as "Booidea", "Anilioidea", "Scolecophidia" and Caenophidia + Tropidophiidae are strongly rejected by molecular and combined evidence studies. Instead, molecular phylogenies (largely, but not exclusively) support a topology in which Tropidophiidae and Anilioidea form a clade,

with the remaining Alethinophidia as their sister group. *Loxocemus* and *Xenopeltis* cluster with pythons, and the relationships among several basal alethinophidian lineages (e.g., boas, pythons, uropeltids, and bolyeriids, as well as between Caenophidia and Henophidia), are poorly resolved.

Rather than being split into a primitive 'Anilioidea' and a derived 'Macrostomata', as has traditionally been the case on the basis of morphological data, 'anilioids' such as *Anilius* and Cylindrophiidae are found to nest within 'macrostomatans', with *Anilius* grouping with Tropidophiidae, and Cylindrophiidae clustering with boas, pythons, and caenophidians. This implies that many apparent plesiomorphies exhibited by 'anilioid' taxa may in fact be secondarily derived, affecting the optimization of these characters, and therefore affecting the placement of fossil taxa available for use as divergence time constraints[37] or tips in combined analyses[30,33,34], including Madtsoiidae and *Dinilysia*.

We elected to favour results from molecular phylogenetic analyses over those from morphological analyses when phylogenetic hypotheses were in conflict, because such results were supported by analyses of both nuclear and mitochondrial loci, were broadly congruent across different loci within these systems, and such analyses provide no support for competing 'morphological' topologies, whereas morphological support for 'molecular' topologies can be found[30]. This, as well as the observation that our own molecular sequence analyses supported prior molecular results, suggests that, when in conflict, molecular topologies are likely to reflect the true phylogenetic history of snakes more closely than do topologies inferred purely on the basis of morphological data. This is significant, because analyses using either combined data[30,33,34] or a molecular constraint of morphological data[37] have resulted in placements of both extant and fossil snakes that are at odds with those inferred on the basis of morphology alone[35,36,38]. The placement of many taxa, including *Dinilysia*, Madtsoiidae, and Simoliophiidae, can be greatly affected, and these have variously been assigned to both the snake stem group and crown group depending on the analysis. In light of this, the placement of these taxa cannot be considered robust, and is instead contingent on whether molecular data are incorporated or not. Given the difficulty of placing relatively complete Cretaceous snakes such as *Dinilysia*, Madtsoiidae, and Simoliophiidae, or even inferring the relationships of extant taxa known from complete skeletons on the basis of morphology, fragmentary vertebrae[5,6] of extinct clades are

likely to be even more problematic—as such, we elected not to incorporate fragmentary vertebrae from the Mesozoic into our calibration scheme.

By contrast, extensive use was made of Cenozoic fossil snakes for the assignment of minimum age calibrations for phylogenetic divergences. We extensively calibrated the snake crown group using Cenozoic fossils. The ability to compare Cenozoic snakes to close extant relatives makes it possible to constrain fossils with a much higher degree of confidence than is possible for fossils of Cretaceous age. Much of this material has not previously been subjected to phylogenetic analysis, but many of these fossils can be constrained with a high degree of confidence due to extensive similarity to extant taxa, or highly autapomorphic morphologies (e.g., the distinctive fangs of Elapidae, or the heat sensitive pit on the maxilla in Crotalinae).

2.2 Calibration justifications

Mammalia

***Tachyglossus aculeatus* — *Homo sapiens* (Monotremata-Eutheria split)**
Min. 164.9 Ma; Max. 201.5 Ma

Min. 164.9 Ma, *Ambondro mahabo* UA-10602[39]. *Ambondro* is considered an australosphenidan (stem monotreme) and calibrates the split between monotremes and therians[26]. We follow Benton *et al*.[26] who assigned *Ambondro* to the Bathonian. The top of the Bathonian is dated to 166.1 ± 1.2[40], giving a minimum age of 164.9 Ma for *Ambondro* and the split between monotremes and eutherians.

Max. 201.5 Ma. Crown mammals first appear in the Jurassic and appear to be a Jurassic radiation based on both fossils[41,42] and molecular divergence dating[23], and the Triassic-Jurassic boundary is therefore used as a conservative estimate for the maximum age of the therian crown.

Crown Euarchontoglires

***Mus musculus* — *Homo sapiens* (Rodentia-Primates split)**
Min. 65.7 MA; Max. 100 Ma

Min. 65.7 Ma, *Purgatorius coracis*, UALVP 16070, holotype isolated right M2, and referred material from the early Puercan (magnetochron C29R) W-1 horizon, Medicine Hat Brick and Tile Quarry, Ravenscrag Formation, Saskatchewan. *Purgatorius* is considered the earliest known primate[43,44], as is supported by phylogenetic analysis of multiple datasets[45]. The C29R interval

represents the first 200-300,000 years of the Paleocene[46,47], so accordingly a minimum age of 65.7 Ma was used here.

Max. 100 Ma. Definitive examples of crown or stem Glires, crown or stem Euarchonta, and crown or stem euarchontoglires are unknown prior to the K-Pg boundary, and definitive examples of crown placentals are unknown with the possible exception of *Protungulatum*, which has been recovered from the latest Cretaceous (late Maastrichtian) of the Hell Creek[48]. The sudden appearance of primates in the basal Paleocene does suggest a Cretaceous split between Euarchonta and Glires, but the absence of a fossil record for these groups or their outgroups argues against an extensive history of Euarchontoglires or crown Placentals in the Cretaceous.

In addition to the absence of fossil evidence, molecular divergence dating puts the origin of crown Euarchontoglires in the latest Cretaceous, circa 75 Ma[23]. Accordingly, given the absence of fossil evidence or molecular signal that the clade extended back to the mid-Cretaceous, 100 Ma is arbitrarily assigned as a maximum for this clade. In light of the most recent molecular dates, this calibration can be considered conservative.

CROWN REPTILIA

Gekko gecko - *Gallus gallus* (Lepidosauria—Archosauromorpha split)
Min 255.9 Ma; Max 299.05 Ma.

Min. 255.9 Ma, *Protorosaurus speneri* RCSHC 308. We follow Benton et al.[26] as considering *Protorosaurus* as the oldest crown reptile.

Max 299.05 Ma. Early Permian assemblages are dominated by stem mammals and rarer stem reptiles, and lack either crown or crown-grade reptiles. We chose the next major geological subdivision below the oldest crown reptile, *Protorosaurus*- the Permian-Carboniferous boundary, approximately 45 million years earlier- as the maximum age for crown reptiles. This is slightly older than the maximum of 295.9 Ma used by Benton et al.[26] and therefore slightly more conservative.

CROWN TESTUDINES

Chelydra serpentina — *Podocnemis expansa* (Cryptodira-Pleurodira split)
Min. 156.3 Ma; Max 201.5 Ma

Min. 156.3 Ma, *Caribemys oxfordiensis* MNHNCu P-3209[49]. *Caribemys* is considered a stem pleurodire and the oldest definitive crown turtle. It is dated to the Oxfordian and the top of the Oxfordian is used as a minimum[49]. The latest dates for the Oxfordian suggest an age of 157.3 ± 1.0 Ma, accordingly an age of 156.3 Ma is used here[40].

Max. 201.5 Ma. Triassic-Jurassic boundary. Crown turtles have an excellent fossil record because they are highly diagnostic and have a high preservation potential: they are relatively large, have dense and durable shells and bones that survive weathering and transport, and inhabit depositional environments such as lakes, rivers, and shallow marine shelves. Accordingly, their fossil record probably corresponds reasonably well to their time of origin, and they are unlikely to

be dramatically older than their first fossil representatives in the middle Jurassic. We chose the next major stage boundary and extinction event below this calibration, the Tr-J, as a maximum for the age of crown turtles.

CROWN ARCHOSAURIA

***Alligator mississippiensis — Gallus gallus* (Crocodylia-Aves split)**

Min. 247.2 Ma; Max 251.926 Ma

Min. 247.2 Ma, *Ctenosauriscus koenini* GZG.V.4191, late Olenekian Solling Formation. Following Benton et al.[26] we use *Ctenosauriscus* as a minimum for crown Archosauria. The fossils are Olenekian in age making them a minimum of 247.2 Ma in age[40].

Max 251.926 Ma. Crown archosaurs are thought to emerge in the early Triassic[50] as part of a recovery fauna that emerged after the Permian-Triassic mass extinction. Accordingly, the Permo-Triassic boundary at 251.926 Ma[40] is used as a maximum.

CROWN CROCODYLIA

***Alligator mississippiensis — Crocodylus porosus* (Alligatoridae-Crocodylidae split)**

Min. 74.74 Ma; Max. 145 Ma

Min. 74.74 Ma, *Albertachampsa langstoni* SMM P67.15.3[51], late Campanian Dinosaur Park Formation, Alberta. *Albertachampsa* is interpreted as a stem alligatoroid. Other possible stem alligatoroids, including *Stangerochampsa mccabei* and *Brachychampsa montana*, appear in the early Maastrichtian[52] and late Maastrichtian[53], respectively, supporting the origin of alligators by the latest Cretaceous. ^{40}Ar-^{39}Ar dates for the base of the Bearpaw Formation, which overlies the Dinosaur Park Formation, indicate an age of 74.98 ±.24, giving a minimum age of 74.74 Ma for the top of the Dinosaur Park Formation and *Albertachampsa langstoni*[54].

Max 145 Ma. Stem alligators appear as successive outgroups to crown Alligatoridae in the Late Cretaceous[55] and archaic aquatic crocodylomorphs, not closely related to Crocodylia, appear in the middle Jurassic.
Crocodylians have a high preservation potential, like turtles, due to their relatively large size, robust skulls and skeletal elements, and aquatic habits; the clade also appears to be Laurasian which increases the odds of fossils being recovered due to a sampling bias towards the northern hemisphere. Accordingly, the next major geological boundary below the calibration, the J-K boundary at ~145, represents a highly conservative maximum.

CROWN AVES

***Dromaius novaehollandiae — Gallus gallus* (crown Aves)**

Min. 66 Ma; Max. 100 Ma

Min. 66 Ma, *Vegavis iaai* MLP 93-I-1[56]. *Vegavis* is widely recognized as a crown member of Aves, although its precise placement as either a crown[56] or stem member[57] of Anseriformes is unknown. The age of *Vegavis* has variously been assigned to 66-68[56] or 66-69 Ma[58]. Accordingly, a minimum of 66 is assigned.

Max. 100 Ma. Mid-Cretaceous. Definitive members of the crown avian orders are not known until the early Cenozoic[59] and a diverse fauna of ornithurines of modern aspect first appear in the late Cretaceous, with the appearance of *Iaceornis* in the Santonian-Campanian Niobrara chalks[60] and diverse ornithurine-dominated faunas in the Campanian[61] and Maastrichtian[62] include ornithurines of modern aspect, which are likely to represent "near-crown" stem members and basal branches of modern avian clades such as Palaeognathae, Galloanseres, and Neoaves. Older bird faunas such as the Jehol conspicuously lack derived Ornithurae. Accordingly, the origin of the avian crown is estimated to lie in the mid-Cretaceous, arbitrarily assigned to an age of 100 Ma.

CROWN LEPIDOSAURIA

Sphenodon punctatus — Gekko gecko (Rhynchocephalia-Squamata split)
Min. 238 Ma

Min. 238 Ma, Vellberg rhynchocephalian, SMS 91060 and 91061. Phylogenetic analysis recovers the Vellberg jaw as a stem rhynchocephalian[63]. The specimen is from a mudstone from the top of the Lower Grey Marls in the Lower Keuper (Erfurt Formation)[63]. U-Pb dating of zircons indicate an age of 238-238.8 Ma[63].

CROWN SQUAMATA

Gekko gecko — Boa constrictor (Geckonidae-Unidentata split)
Max. 251.926 Ma

Max. 251.926 Ma. The oldest fossils that appear referable to crown Squamata are Middle Jurassic in age[35,64,65]. Crown squamates are conspicuously absent from the Triassic[66], the fossils suggest an origin of crown squamates in the Jurassic or perhaps late Triassic. Molecular clocks typically recover an origin of crown squamates either in the Jurassic[63] or the Late Triassic[1,67]. Accordingly, the constraint used here, the Permian-Triassic boundary, is highly conservative. The latest dates for the Permian-Triassic boundary recover an age of 251.902 ± 0.024 Ma[40] and so a maximum age of 251.926 is used here.

Eublepharis macularius—Gekko gecko (split between Gekkonidae and Eublepharidae),
Min. 43 Ma.

Min. 43 Ma, *Yantargekko balticus* GAM 1400 is known from Baltic amber from the Lower Eocene Samland Peninsula[68] and is referred to the gekkonid subclade Gekkonidae on the basis of the loss of movable eyelids and enlarged scansorial pads[68]. The specimen's provenance is proven by the presence of numerous stellate oak bud trichomes and a dolichopodid fly[68]. The Baltic Amber is dated to 44.1 ±1.1 Ma and so a minimum of 43 Ma[69] has been used. This calibration could potentially calibrate a more exclusive clade- Gekkonidae + Phyllodactylidae- but constraints on

taxon sampling mean that it is used to calibrate the more inclusive Eublepharidae-Gekkonidae clade.

CROWN DIPLODACTYLOIDEA

Lialis burtonis—Strophurus ciliaris (Pygopodidae-Diplodactylidae split)
Min. 20 Ma

Min. 20 Ma, *Pygopus hortulanus* QMF 16875 is referred to the Pygopodidae[70] on the basis of the stout, straight dentary, low dentary tooth count, and robust teeth, a combination of characters unique to Pygopodidae among the Gekkota[70]; subsequent studies have corroborated this assessment[71]. The deposits at Riversleigh that produced *P. hortulanus* are dated to 20-22 Ma[70,71]. This constrains the split between pygopodids and all other geckos to a minimum of 20 Ma.

UNIDENTATA

Boa constrictor–Scincus scincus (Toxicofera-Scinciformata split)
Min. 145 Ma; Max. 201.5 Ma

Min 145 Ma, *Paramacellodus* cf. *P. oweni* DINO 15914, 14864. *Paramacellodus* sp. from the Brushy Basin member of the Morrison Formation of Utah[72] is among the oldest lizards referred to the crown. Phylogenetic analysis suggests that *Paramacellodus* is a scincomorph[35], a result that is recovered in both morphology-only[35] and combined analyses[30] and therefore robust against different approaches to phylogenetic reconstruction.

The Brushy Basin member is Tithonian in age based on single crystal, laser-fusion ^{40}Ar-^{39}Ar dates[73] and single-crystal U-Pb zircon dates[74]. The top of the Tithonian at 145 Ma[75] is therefore used as a minimum age.

Eichstaettisaurus schroederi BSPG 1937[35] represents another possible calibration for the crown. *Eichstaettisaurus* (coded as a composite of *E. schroederi* and Early Cretaceous *E. gouldi*) was recovered as a stem gecko[35]. *Eichstaettisaurus schroederi* is from the Solnhofen lithographic limestone[76], which is Tithonian in age[77], and therefore has a minimum age of 145 Ma[75]. However, the placement of *Eichstaettisaurus* is not stable under different phylogenetic approaches; it is a stem gecko in a morphology-only topology[35] but a stem squamate in combined analyses[30].

Other possible calibrations for crown Unidentata also exist but are based on material that is either fragmentary or whose affinities remain uncertain. *Parviraptor* has been interpreted as an anguimorph[65] or a stem snake[78]. The uncertainty about its placement, and which material can be referred to these fossils[65,78], makes its use as a calibration problematic (see below).

Max 201.5 Ma. The earliest squamate fossils that appear referable to the crown are from the Middle Jurassic[35,64,65]; no crown squamates are known from the Triassic. This suggests a Jurassic origin for crown Squamata and more exclusive clades such as Unidentata. Accordingly, the Triassic-Jurassic boundary at 201.3+- 0.2[40] is used as a maximum constraint for Unidentata.

SCINCOMORPHA

Xantusia vigilis—Cordylosaurus subtessellatus (Xantusiidae-Cordylidae split)

Min. 93.9 Ma

Min. 93.9 Ma, *Utahgenys* sp. MN V9113, Cenomanian Dakota Formation[79]. The Cretaceous *Contogenys* and related taxa were recently identified as stem members of the Xantusiidae[79]. Derived features shared with Contegeniidae and Xantusiidae include enlarged posterior marginal teeth with blunt, chisel-shaped apices, an enlarged dentary coronoid process, reduction of the posterior emargination of the dentary, and an elongate angular process projecting well below the coronoid process. The contogeniid *Utahgenys* is found in the Cenomanian-Turonian of Utah[79] with older specimens in the Cenomanian; accordingly a minimum age of 93.9 (Cenomanian-Turonian boundary) is used for the divergence between xantusiids and cordylids.

***Xantusia vigilis — Lepidophyma flavimaculatum* (Xantusiinae—Lepidophyminae split)**
Min. 55.8 Ma

Min 55.8 Ma, *Palaeoxantusia* sp. CG (UCMP 150848, 150974) and Xantusiid CG (UCMP 150965), Castle Gardens Fauna, Bighorn Basin, Paleocene-Eocene Boundary[80]. Both members of the *Xantusia* lineage (*Palaeoxantusia* sp. CG) and the *Lepidophyma* branch of the Xantusiidae (Xantusiid CG) are present in the assemblage, constraining the minimum age of the split between *Xantusia* and *Lepidophyma*.
The Castle Garden fauna lies in the Wasatchian 0 interval[80]. Wa-0 in turn corresponds to the PETM event[81], with the onset of Wa-0 and the PETM warming documented by both the arrival of new species such as horses in the Western Interior, and dwarfing of mammal species; the end of the Wa-0 interval is in turn marked by an increase in size of mammals, corresponding to cooling associated with the end of the PETM[81]. The onset of the PETM is at the Paleocene-Eocene boundary at 56 Ma[40,82] and the event lasted for approximately 200 Ka[82], constraining the minimum age of these fossils to 55.8 Ma.

***Tiliqua scincoides— Trachylepis quinquetaeniata* (Australian and non-Australian skink split)**
Min. 24 Ma.

Min 24 Ma, *Proegernia palankarinnensis* SAM P39204[83]. *Proegernia* is referred to the "*Egernia* group" based on the closed Meckelian groove and the size, shape, and position of the alveolar foramen. As an Australian lygosomine it can be constrained to an Australian endemic clade, constraining the timing of the split between Australian endemic lygosomine skinks and non-Australian forms. The most closely related non-Australian species used in this analysis is *Trachylepis quinquetaeniata*, and it is thus used to calibrate the node. *P. palankarinnensis* was recovered from the lower Etadunna Formation Minkina Local Fauna[83]. It is dated to 24-26 Ma based on land mammal biostratigraphy and magnetic polarity zonation[84], in the late Oligocene[83] accordingly a minimum of 24 Ma is used here.

***Cordylosaurus subtessellatus –Zonosaurus ornatus* (Gerrhosaurinae-Zonosaurinae split)**
Min. 15 Ma

Min. 15 Ma, *Gerrhosaurus* cf. *major* KNM-RU2605, Miocene of Rusinga Island, Kenya. A partial mandible of a gerrhosaur, identified as *Gerrhosaurus* cf. *major*[85] is known from the Miocene of Rusinga Island in Kenya. The fossil is identified as *Gerrhosaurus* on the basis of its large size and

wrinkled tooth crowns[85]. The Kulu Formation of Rusinga is estimated at 15-17.8 Ma[86] and accordingly a maximum of 15 Ma is used here.

CROWN TEIIDAE

***Tupinambis teguixin — Callopistes maculatus* (*Tupinambis-Callopistes* split)**
Min. 20 Ma

Min. 20 Ma, *Tupinambis* sp. (numerous fragmentary fossil fragments, see [87] for all specimen numbers), Sarmiento Formation, Argentina. A tupinambine referred to *Tupinambis* is reported from the Trelew Member of the Early Miocene Sarmiento Formation at Gaiman, Chubut Province, Patagonia[87]. Referral to *Tupinambis* is based on the presence of blunt posterior teeth, the occasional enlarged 3rd maxillary tooth, and multiple other characters[87]. The fossils come from the Colhuehuapian mammal zone. Radiometric dates for the Colhuehuapian mammal zone faunas in the Sarmiento Formation at Gran Barranca span 20.0-20.4 Ma[88].

CROWN LACERTIBAENIA

***Rhineura floridana – Lacerta viridis* (Lacertidae-Amphisbaenia split)**
Min. 66 Ma

Min. 66 Ma. *Chthonophis subterraneus* AMNH 30799, K-Pg boundary, 66 Ma[89]. Placement of *Chthonophis* in the Amphisbaenian crown[89] would instead calibrate crown Amphisbaenia (Rhineuridae-Amphisbaenidae split) but *Chthonophis* represents a highly unusual amphisbaenian and so its affinities are somewhat uncertain. It is therefore used to calibrate the more inclusive clade of Lacertibaenia. *Chthonophis* was found in the Maastrichtian to Paleocene Bug Creek Anthills[89]. It is believed to be earliest Paleocene in age[89].

CROWN AMPHISBAENIA

***Rhineura floridana – Amphisbaena fuliginosa* (Rhineuridae-Amphisbaenidae split)**
Min. 62 Ma

Min. 62 Ma. *Plesiorhineura tsentasi*, UNM NP-596. *Plesiorhineura* exhibits typical rhineurid characters, including apically positioned teeth, a closed Meckelian groove, and a long, narrow dentary process of the coronoid overlapping the dentary laterally beneath the toothrow[90]. *Plesiorhineura* is from the '*Pantolambda* Zone' of the Nacimiento Formation [90], which corresponds roughly to the Torrejonian 3 Land Mammal Age[91] To3 is correlated with the end of magnetochron C28r through the beginning of C27r[91], approximately 62-63 Ma[75]. As a very primitive rhineurid, *Plesiorhineura* was used to calibrate the split between Rhineuridae and Amphisbaenidae.

***Bipes biporus– Amphisbaena fuliginosa* (Bipedidae-Amphisbaenidae split)**
Min. 55.8 Ma

Min. 56 Ma, *Anniealexandria gansi*, UCMP 167594, Paleocene-Eocene boundary (PETM)[80]. *Anniealexandria* is recovered as a stem bipedid[89] suggesting divergence between Bipedidae and their sister taxon Amphisbaenidae. It can be excluded from Rhineuridae based on the open Meckelian groove[80]. The fossils occur in a layer that contains the Paleocene-Eocene carbon isotope excursion that marks the PETM and are assigned to earliest interval of the Wasatchian, Wa-0[80]. As discussed above, Wa-0 corresponds to the PETM[81]. The onset of PETM is the Paleocene-Eocene boundary at 56 Ma[40,82] and it lasted approximately 200 Ka[82], constraining the minimum age of the fossils to 55.8 Ka.

***Geocalamus acutus—Amphisbaena fuliginosa* (*Geocalamus-Amphisbaena* split)**
Min. 15 Ma

Min. 15 Ma, *Listromycter leakyi*, BMNH R.8292[92]. Phylogenetic analysis recovers *Listromycter* as sister to *Monopeltis*[89]. *Listromycter* comes from the Early Miocene of Rusinga Island, in Kenya[92]. Kulu Formation of Rusinga is estimated at 15-17.8 Ma[86]. *Monopeltis* is not included in the current dataset, and so instead this constraint was used to provide a constraint on a slightly deeper split, between *Geocalamus* and the remaining Amphisbaenidae.

IGUANIA

***Basiliscus basiliscus—Agama agama* (Iguanidae-Agamidae split)**
Min. 71 Ma

Min. 71 Ma, *Isodontosaurus gracilis* AMNH 6647[93]. Phylogenetic analysis of referred specimens recover *Isodontosaurus* as a stem iguanid[35], so it is used here to calibrate the divergence between Iguanidae and Agamidae, and thus the age of crown Iguania. *Isodontosaurus* comes from the Djadokhta Formation exposures at Bayn Dzak ("Flaming Cliffs"). The precise age of the Flaming Cliffs is not well-constrained. Magnetostratigraphy suggests that the formation is younger than magnetochron 34n, the top of which lies near 84 Ma[75], and the beds may lie between 75-71 Ma[94]. Accordingly, a minimum of 71 is used here.

***Uromastyx aegyptia—Agama agama* (Uromasticinae-Agaminae split)**
Min. 47.8 Ma

Min. 47.8 Ma. The oldest known member of the uromastycine agamids is known on the basis of jaw fragments (ZIN PH 1-4/1, 6/1, 8/1) from the Early Eocene (Late Ypresian) Alay beds at the Andarak 2 locality of Kyrgyzstan[95]. These jaws are referable to the Uromastycinae as defined by Alifanov[96] on the basis of numerous features, including tooth crowns that are low, closely set, and with crescentic crowns lacking accessory cusps, and an elevated coronoid process of the dentary[95]. A late Ypresian age suggests a minimum date of 47.8 Ma[75] for the divergence between uromastycines and all other Agamidae.

CROWN PLEURODONTA

Basiliscus basiliscus-Anolis carolinensis (Corytophanidae-Dactyloidae)
Min. 55.8 Ma

Min. 55.8 Ma. The oldest known crown iguana is *Suzaniwanna patriciana* UCMP 167664[80] which is identified as a stem corytophanid on the basis of cladistic analysis[97], although it has no apomorphies. It is here used as a constraint for the minimum age of the divergence between Corytophanidae and Dactyloidae + Hoplocercidae.
Suzanniwanna comes from UCMP locality V99019, part of the Willwood Formation, Bighorn Basin, which represents the earliest part of the Wasatchian land mammal age, Wasatchian 0. Wa-0 starts at the Paleocene-Eocene boundary and spans the PETM[81]. Wasatchian 0 is therefore constrained to between 56 Ma, the Paleocene-Eocene boundary[40,82], and 200 Ka later, at the end of the PETM[82], constraining the minimum age of these fossils to 55.8 Ma.

CROWN ANGUIMORPHA

Varanus salvator—Shinisaurus crocodilurus (Varanidae-*Shinisaurus* split)
Min 98.39 Ma

Min. 98.39 Ma*, Primaderma nessovi* OMNH 26742[98] exhibits multiple apomorphies of the Varanoidea, including enlarged, recurved teeth, crowns with well developed, serrated carinae, and expanded and infolded tooth bases which lack replacement pits. Although *Primaderma* and other Cretaceous varanoids have been considered to be related to helodermatids, it is here considered a stem varanoid following a number of recent studies referring Cretaceous varanoid-like taxa to the varanoid stem[30,35,99] ; helodermatids appear to represent a lineage unrelated to the Varanidae[32] appearing in the Eocene[100]. *Primaderma* is from OMNH locality V695 the Mussentuchit Member of the Cedar Mountain Formation, dated to 98.39 Ma using volcanic ash layers[98].

Varanus salvator— Lanthanotus borneensis (Varanidae-Lanthanotidae split)
Min. 48.5 Ma

Min. 48.5 Ma. The early varanid *Saniwa* FMNH PR2378[101] from 'locality H' in the Green River Formation has been recovered as a stem varanid on the basis of phylogenetic analysis[30,35]. It is therefore used here to constrain the split between Varanidae and its sister taxon, Lanthanotidae. The Green River Formation spans a period from 53.5-48.5 Ma based on argon-argon dating of tuff beds[102].

Varanus salvator—Varanus acanthurus
Min 23 Ma

Min. 23 Ma. Varanidae are present in Australia in the Etadunna Formation[103] at the Oligocene-Miocene boundary, at 23 Ma[40], indicating a split between Australian varanids (the lineage leading to *V. acanthurus*) and other forms by this time[104].

***Xenosaurus grandis —Pseudopus apodus* (Xenosauridae-Anguidae split)**
Min. 78 Ma

Min. 78 Ma. The split between Anguidae+Anniellidae and other Anguimorpha (in our analysis, Xenosauridae is the sister clade) is documented by the presence of the anguid *Odaxosaurus* in the Late Cretaceous of Western North America[105]. The oldest well-documented report of *Odaxosaurus* is from the Middle Campanian (Judithian Land Mammal Age) Clambank Hollow fauna, in the Judith River Formation of Montana[106]. Judith River exposures to the north are dated to approximately 78 (78.2 +- 0.2) Ma[107].

***Pseudopus apodus— Elgaria multicarinata* (Anguinae—Gerrhonotinae split)**
Min. 55.8 Ma

Min. 55.8 Ma. An anguid exhibiting derived characters of the Gerrhonotinae, Gerrhonotine CG, is united with Gerrhonotinae based on its reduced crista transversalis which trends longitudinally along most of the premaxillary process, an elongate premaxillary process, and fused frontals[80]. It is identified from the PETM (Wasatchian 0) interval of the Bighorn Basin Castle Gardens fauna[80]. The fossils occur in a layer that contains the Paleocene-Eocene carbon isotope excursion that marks the PETM[80] starting at approximately 56 Ma[40]. The PETM lasted approximately 200 Ka[82] constraining the age of the fossils to approximately 55.8 Ma.

CROWN TOXICOFERA

***Boa constrictor—Varanus salvator* (Serpentes-Anguimorpha split)**
Min. 113 Ma

Min. 113 Ma, *Tetrapodophis amplectus* BMMS BK 2-2[37]. *Tetrapodophis amplectus* is recovered as a stem snake on the basis of multiple derived characters of the teeth, jaws, vertebrae, pelvis, and hindlimbs, based on either morphology-only, constrained[37] or combined analyses[33]. Further discussion of *Tetrapodophis* and parviraptorids as calibrations for this node can be found in section 1.3 below.

AMEROPHIDIA

***Tropidophis haetianus-Anilius scytale* (Tropidophiidae-*Anilius* split)**
Min. 61.6 Ma

Min. 61.6 Ma, *Kataria anisodonta*, Museo de Historia Natural de Cochabamba 13323. *Kataria*[108] is here identified as a tropidophiid based on a combination of characters that includes a strongly inflated parietal, well-developed orbital ridge of the frontals, a forked postorbital, and a vomerine process of the palatine that turns laterally to buttress the vomer. This placement has been validated by phylogenetic analysis of constrained[37] and combined data[33]. *Kataria* comes from the Tiupampa locality of Bolivia, which is estimated to be Danian in age based on faunal composition and correlation to isochrons[108] making *Kataria* and the tropidophiid-aniliid split no younger than 61.6 Ma[75].

***Loxocemus bicolor-Python molurus* (Loxocemidae-Pythonidae split)**
Min. 35.2 Ma

Min. 35.2 Ma, *Ogmophis compactus* PTRM 19378, quadrate, Chadronian aged Chadron Formation, Medicine Pole Hills of North Dakota. Smith[109] has identified an isolated quadrate as a very likely relative of *Loxocemus* and *Xenopeltis,* based on its highly expanded and twisted dorsal head and vertebral similarities of associated material, constraining the split between *Loxocemus* and pythons. These fossils are dated to 35.2 Ma based on $^{40}Ar/^{39}Ar$ dating and sediment accumulation rates[109,110].

***Lichanura trivirgata-Exiliboa placata* (Charininae-Ungaliophiinae)**
Min. 35.2 Ma

Min. 35.2 Ma, *Calamagras weigeli,* PTRM 19607, caudal vertebra, Chadronian aged Chadron Formation, Medicine Pole Hills, North Dakota[109]. Smith[109] identified an isolated caudal vertebra as having features unique to *Ungaliophis* and *Exiliboa* among extant snakes due to the absence of hemapophyses, constraining the minimum age of the split between Ungaliophiinae (family to which *Ungaliophis* and *Exiliboa* belong) and their closest relatives, the Charininae[109]. The fossils are dated to 35.2 Ma[109,110].

***Python molurus-Aspidites melanocephalus* (*Python*-Australian pythonid split)**
Min. 12.5 Ma

Min. 12.5 Ma, *Morelia (Montypythonoides) riversleighensis*[111] documents the oldest known occurrence of pythons in Australia. It has been referred to *Morelia*, a paraphyletic assemblage of Australian pythonids; based on biogeography an assignment to the Australian clade of pythons appears probable, and therefore these fossils are used to calibrate the split between the Asian *Python* and the Australian clade. This assemblage is dated to 12.5 Ma based on faunal correlation to the Bullock Creek local fauna[5].

CROWN CAENOPHIDIA

***Coluber constrictor-Acrochordus javanicus* (Acrochordidae-Colubridae split)**
Min. 50.5 Ma

Min. 50.5 Ma. Rage et al.[112] report a caenophidian from the Early Eocene (Ypresian) of the Vastan Lignite Mine, in India, *Procerophis sahnii* VAS 1014. The elongation of the centrum, long and low neural spine, and paracotylar foramina represent colubroid affinities. The fossils have since been constrained more tightly, on the basis of planktonic foraminifera, to the middle Ypresian, 53-50.5 Ma, with the younger date used here[5].

***Coluber constrictor-Naja naja* (Elapidae-Colubridae split)**

Min. 30.8 Ma

Min. 30.8 Ma, *Coluber cadurci* MGT 3505 Rage[113]. *Coluber cadurci* is referred to the Colubroidea+Elapidae clade based on the prominent and narrow haemal keel, epizygaophyseal spines and long prezygapophyseal accessory processes[6]. It is dated to Paleogene mammal biochron 22, whose top lies at 30.9 ±.1 Ma, accordingly a minimum of 30.8 is used here. The slightly older *Texasophis galbreathi* dates to approximately 32 Ma[114], however skepticism has been expressed about the referral of these fossils to this clade[6].

***Boaedon fuliginosus-Naja naja* (Elapidae-Lamprophiidae divergence)**
Min. 20.44 Ma

Min. 20.44 Ma. Oppenheim-Nirstein quarry elapid (Fig. 1A[115]). Diagnostic fangs of elapids are found in the Oppenheim/Nierstein quarry in the Mainz Basin of Rhineland-Palatinate, Germany[115]. Elapid fangs are identifiable and can be distinguished from viperid fangs because the venom canal is closed but not fused, leaving a distinct suture (a plesiomorphy); furthermore, the venom canal is twisted such that the distal opening of the venom canal is visible in lateral view (an autapomorphy). The beds are assigned to the Aquitanian age of the Early Miocene[115], which span a period from 20.44-23.03 Ma[75]; a minimum of 20.44 Ma is therefore used here.

***Notechis scutatus-Micrurus fulvius* (*Micrurus*-Hydrophiinae divergence)**
Min. 10 Ma

Min. 10 Ma, *Incongruelaps iteratus* (holotype QM F42691, maxilla QM F23085) is an elapid from the Encore Site at the Riversleigh fossil site in Australia[116]. Elapid features include large hollow fangs (found convergently in viperids) separated from the posterior teeth by a diastema, and a prominent medially projecting ectopterygoid process. Scanlon et al.[116] argued that the fossil is nested within the Hydrophiinae, a clade that includes true sea snakes (*Hydrophis* and kin), sea kraits (*Laticauda*), and Australian terrestrial Elapidae[32].

Scanlon et al.[116] suggested that the fossil could be nested inside crown Hydrophiinae but note that the fossil exhibits "a unique combination of derived characters otherwise found in several extant hydrophiine taxa that are only distantly related". Although it seems likely (particularly given the existence of a possible *Laticauda* in the same formation)[116] that this fossil represents a crown hydrophiine, we instead chose to be more conservative and calibrate a deeper node, the split between stem Hydrophiinae and Micrurus. The site is about ~ 10 Ma based on mammalian biostratigraphy [116].

***Crotalus viridis-Daboia russelii* (Viperinae-Crotalinae divergence)**
Min. 9.7 Ma

Min. 9.7 Ma. Kalfinsky Formation crotaline, IZAN 3748[117]. A maxilla from the Gritzev locality in Kalfinsky Formation in the Ukraine (IZAN 3748)[117] shares with vipers an extreme anteroposterior compression of the maxilla, reduction of the dentition to two tooth positions, and hollow fangs. It can further be identified as a crotaline by a large embayment that accommodates the heat-

sensitive pit organ in extant pitvipers[117]. The locality is assigned to the MN9 mammal biozone, which extends from 11.1-9.7 Ma[118].

ISLAND CALIBRATIONS

Mitophis pyrites—Mitophis asbolepis (Hispaniola leptotyphlopid clade divergence)
Max. 10 Ma

Max. 10 Ma, *Mitophis pyrites* and *M. asbolepis* are part of a clade of Leptotyphlopidae endemic to the island of Hispaniola[25]. Following Vidal et al.[25] we used a maximum age of 10 Ma[119] for the island of Hispaniola. As a clade originating on an island must postdate the island itself, this is taken as a maximum for the clade.

Typhlops agoralionis—Typhlops jamaicensis (Hispaniola typhlopid clade divergence)
Max. 10 Ma

Max. 10 Ma, *Typhlops agoralinionis* and *T. jamaicensis* are nested within a clade of Typhlopidae endemic to the island of Hispaniola. We follow Vidal et al.[25] in using a maximum age of 10 Ma[119] for the island. As a clade originating on an island must postdate the island itself, this is taken as a maximum for the clade.

Conolophus subcristatus — Conolophus pallidus (*Conolophus* diversification)
Max. 5 Ma

Max. 5 Ma, age of the oldest Galapagos Islands[120]. The oldest emergent Galapagos Islands are dated to 4-5 Ma[120].

The existence of older seamounts, with evidence of erosion suggesting that they potentially represent formerly emergent islands[121], does raise the possibility that the Galapagos Archipelago could substantially predate the existing islands and that Galapagos endemic radiations may predate the islands themselves[121,122]. However, it is difficult on the basis of geology to establish with certainty whether a continuously emergent archipelago predates the current archipelago, and if so, by how long.

Molecular clock studies could help constrain the age of the fauna. Older molecular divergence estimates for e.g. Galapagos iguanas suggest ages of up to 10 Ma[122], suggesting that some island radiations predate the current islands. However, more recent analyses of Galapagos iguanas[123], Galapagos lava lizards[124], Galapagos tortoises[125] and Galapagos finches[126] all indicate that these radiations are comparable in age to (*Amblyrhynchus* + *Conolophus*), or markedly younger than (all others), the oldest islands. Congruent results from multiple unrelated taxa with distinct ecologies and dispersal patterns therefore argue for a young origin of the Galapagos fauna, postdating the modern islands, rather than an origin on an older, and now submerged, island.

Accordingly we use the age of the oldest islands as a constraint, with the older end of the range taken as the maximum. Current molecular ages for *Conolophus* + *Amblycristatus* are 4.48 Ma (3.18-5.85 Ma 95% credibility interval) and the age for the divergence of a paraphyletic *C. subcristatus* containing *C. pallidus* is 290 Ka (150- 530 Ka 95% credibility interval)[123]. Given that a

maximum of 5 Ma is over 10 times as old as the current molecular dates for this split, this maximum is highly conservative.

Microlophus delanonis — Microlophus albemarlensis
Max. 5 Ma

Max 5 Ma. Age of the oldest Galapagos islands[120]. The rationale for the choice of 5 Ma as a rationale is discussed above. As with *Conolophus*, the age of the lava lizard radiations are currently reconstructed as markedly younger than the oldest Galapagos islands[124]. As a result, the use of a maximum of 5 Ma is conservative.

2.3 Notes on excluded calibrations

Our analysis excluded a number of calibrations previously used by other researchers. A detailed rationale for every exclusion is beyond the scope of this project, but a few potentially important calibrations deserve commentary here.

Calibrating the snake stem lineage

Here we have calibrated the divergence of crown Toxicofera using the stem snake *Tetrapodophis*[37]. This is a conservative calibration, much younger than age estimates recovered from molecular clock analyses (see Figs S4 – S21). We acknowledge that there has been discussion of the validity and phylogenetic position of this taxon. However, we note that thus far (and to the extent that this has a bearing on its use as a calibration point), all analyses including both extensive taxon sampling and character sampling, either with morphology or combined data, have recovered *Tetrapodophis* as a stem snake[33,37]. Even if *Tetrapodophis* were to represent an early member of Mosasauroidea, as has been proposed[127], in this hypothesis mosasaurs are recovered as stem snakes, and so it would calibrate the same node as in the original phylogenetic placement. We are confident that *Tetrapodophis* derives from the Crato Formation. Each locality has a distinct taphonomic profile, composition of the matrix, and colour of the bone. As discussed in the original paper, all available lines of evidence (matrix, preservation of bone, associated coprolites) point to a Crato origin for *Tetrapodophis*, and are inconsistent with any other known locality[37]. Given that *Tetrapodophis* was used as a conservative calibration in conjunction with numerous other calibrations, we consider that its inclusion or otherwise is unlikely to have had a significant effect on the outcome of our molecular clock analyses. This is supported by our observation that an alternative calibration based on *Parviraptor* only induced slightly older node ages (see Figs. S4, S6, S12, S13 for comparisons).

Parviraptor and kin provide an alternative minimum age calibration for the snake-anguimorph split. These taxa are predatory lepidosaurs known from the middle Jurassic to earliest Cretaceous of Europe and North America[78]. *Parviraptor* has been included in multiple phylogenetic analyses over several decades[33,37,65,78,128,129]. Initially described in 1994, it was considered to not show enough diagnostic characters to support a confident referral to the snake total clade[65]. Phylogenetic analyses of morphological data have variably recovered it as a stem snake[78], an anguimorph[128], basal to Scleroglossa[128], or a basal gekkonomorph[36]. Evans and Wang[128] analysed

Parviraptor across multiple morphological matrices, and recovered low support for any position. More recent analyses based on an updated matrix from Caldwell et al.[78] find parviraptorids near the base of their trees, either in a polytomy with clades representing stem snakes and crown snakes+*Coniophis* (parsimony analysis), or at the root with *Varanus* (the outgroup taxon) and Ophidia (Bayesian analysis)[129]. Thus, parviraptorids were not used as calibrations in our main analyses due to this ongoing uncertainty about their taxonomic position compounded by known issues within morphological squamate datasets (extensive homoplasy, focus on cranial characters), and the difficulties associated with assessing the depositional environment of Durlston Bay (the locality of the oldest specimens of *Parviraptor*) and its effect on interpretation of fossils.

Simoliophiidae as crown Alethinophidia

Simoliophiidae, which exhibit well-developed hindlimbs, were originally interpreted as stem Serpentes[130] but have been recovered as crown members of Alethinophidia by the majority of more recent phylogenetic analyses, including morphology-only[35,38,131–133], constrained[37], and combined[30,33,34] analyses.

Despite this, considerable uncertainty exists about the placement of Simoliophiidae. They are variously recovered as stem Alethinophidia[34,37], crown Alethinophidia[34,35], or within the *Anilius* + *Tropidophis* clade[30]. In a small but potentially significant set of analyses, they are recovered as stem Serpentes[134], or unresolved with respect to crown snakes[78]. Accordingly, the position of Simoliophiidae cannot be considered settled. Although the skull is highly derived[131], the large, well-developed hindlimbs and pelvis are much more primitive than in any crown snake. This raises the possibility of either multiple losses of large hindlimbs in crown snakes (assuming Simoliophiidae belong to the crown), or that the derived skull and jaw morphology seen in the Simoliophiidae has been lost in various “Scolecophidia” perhaps as a result of their extensive specialization for a subterranean lifestyle.

Simoliophiidae first appear in the Cenomanian (93.9 – 100.5 Ma[75]) and specifically are known from the lower Cenomanian[11]. This is almost perfectly within the range of the 95% confidence interval recovered in the PAML analyses here with a skew-T prior (see Fig 1.), for the split estimated for Alethinophidia and Anomalepididae (100.64-93.19 Ma, mean 96.71 Ma).

Coniophis as crown Alethinophidia

The late Maastrichtian *Coniophis precedens* has traditionally been interpreted as a member of the 'anilioid' snakes[105]. More recently, jaws found in the same deposits as the vertebrae and referred to *Coniophis* by Estes[105] have been interpreted as coming from a stem snake[38], an interpretation supported by subsequent analyses[33,34,37,78]. This would prevent *Coniophis* from being used to calibrate the crown or any subclades within the crown. Although the bulk of the evidence suggests that Estes was correct[38] the assignment of this dissociated material has remained controversial[78]. In either case, whether the vertebrae and skull material is associated or not, there is no strong evidence to suggest placement of *Coniophis* in the crown, and thus the use of *Coniophis* to calibrate splits within the crown[1,135] is not justifiable.

Using geological events to inform new analyses

Vidal et al.[31] used the geological age of the Antilles to calibrate the split between *Trachyboa* and *Tropidophis*, whilst Vidal et al.[25] used the opening of the Atlantic Ocean to calibrate the split between Amerophidia and Afrophidia. In the former analysis, the use of the geological calibration is considered justified as results recovered with it included tend to recover ages within the credibility intervals derived from analyses where it was excluded. However, the 3/21 nodes where this does not hold are consecutive Caenophidian nodes, revealing the considerable impact of the geological calibration elsewhere in the tree.

Vidal et al.[25] justify the use of a geological calibration by referring to Vidal et al.[31]; the earlier analysis suggests a divergence between Afrophidia and Amerophidia driven by vicariance. The calibration choice for Vidal et al.[25] is therefore not independent of the 2009[31] analysis, causing a circular set of results. The use of geological events as calibrations based on the assumption of vicariance has been cautioned against by multiple recent studies[136], and an assumption of vicariance has therefore not been made in any of the calibration choices here.

The exclusion of the Vellberg jaws

Jones et al.[63] described a new *cf. Diphydontosaurus* rhynchocephalian jaw fragment. It is at least 238 Ma old, however recent analyses[1,135] continue to set a maximum age of crown Lepidosauria at 222.8 Ma. It should be noted that this age estimate for the fossil the calibration is based on, the Vinita specimen[137], is outdated[63]. Based on the updated 2017 International Chronostratigraphic Chart[40], the Vinita specimen would date to ~237 Ma, as it originates from the Ladinian-Carnian

boundary. An alternative specimen to the Vinita fossil does exist; *Brachyrhinodon* is from the Carnian of the UK[138], dating it to ~227 to ~237 Ma. It is a relatively derived rhynchocephalian, further supporting the prior existence of the clade. The Vellberg jaw, *cf. Diphydontosaurus,* remains the oldest known rhynchocephalian at 238-240 Ma and was used here as a calibration to the exclusion of the Vinita specimen and *Brachyrhinodon*.

2.4 Rationale for choice of clock model

The molecular clock models used for divergence time dating can be divided into two general classes: strict and relaxed. The earliest implementations assumed that the rate at which mutations accumulate is homogenous, and does not vary between lineages[139]. While this assumption may hold in some cases[140], most genetic data depart from a strict clock model, with different rates pertaining along different branches. This prompted the development of models that relax the assumption of a strict molecular clock— relaxed clock models[139]. Relaxed clock models allow rates to vary between branches, and models differ in how rates are allowed to change across branches. Two commonly used variants are the autocorrelated relaxed clock and the uncorrelated relaxed clock models[139]. Appropriate model choice is vital, since results from different models can differ markedly - Tao *et al.* document cases where uncorrelated models report both younger and older ages than their correlated counterparts[141,142]. More complex models are not always preferable, and over-parameterization can be detrimental[143].

Both autocorrelated and uncorrelated models assume that rates are constant along branches, but allow evolutionary rates to vary between branches. The two differ in one key assumption: the effect of relatedness on evolutionary rates.

In *autocorrelated relaxed clock* models, closely related lineages are assumed to evolve at similar rates. In this model, if a lineage evolves quickly, its daughter and sister lineages are likely to evolve quickly, and if it evolves slowly, related lineages are likely to evolve slowly[139].

In *uncorrelated relaxed clock* models, rates of molecular evolution are not correlated with evolutionary relationships. If one lineage evolves quickly or slowly, closely related lineages are not assumed to be evolving at similar rates[139].

Ideally, choice of model should be based on strong theoretical and/or empirical arguments independent of the hypothesis being tested; i.e. models should be chosen without making any assumptions about how the particular group in question is expected to evolve, including patterns of extinction or radiation. The autocorrelated model provides a more realistic description of molecular evolution[141]. Until recently, there has been little empirical evidence to favour its use[141], but new analyses suggest that autocorrelation is likely too widespread in molecular data for easy detection[142]. Here we outline the theory and evidence behind the choice of the autocorrelated molecular clock model as the main clock model used in our analyses.

I. Theoretical basis for the autocorrelated molecular clock

Both intrinsic aspects of organisms' biology (genes) and extrinsic biological factors (environment) are likely to control the rate of molecular evolution.

IA. Intrinsic Effects on Evolutionary Rate

Closely related lineages are likely to share those aspects of their biology such as DNA repair mechanisms, metabolic rate, and generation time that affect the mutation rate[144]. Therefore, their molecular sequences should evolve at more similar rates than those of more distantly related lineages.

Generation time. The generation time hypothesis holds that organisms with short generation times go through the cycles of cell division needed to produce gametes more often over a unit of time than organisms with longer generation time[145]. This has been demonstrated in mammals[145,146] and in invertebrates[147]. Since generation time tends to be nonrandomly distributed across a phylogeny, we also expect some correlation of rates across the tree.

Metabolic rate. Mass-specific metabolic rate (energy consumption per unit mass) affects rates of molecular evolution[148]. This has been proposed to result either from higher rates of damage to DNA caused by free radical production, increased mutation rates caused by more rapid copying of DNA (generation time hypothesis) or both[148,149].

Body size. Several studies find that body size negatively correlates with evolutionary rate, likely due to the fact that it co-varies with generation time and metabolic rate[146,149].

IB. Extrinsic controls on rate of evolution

Evolutionary rates are also affected by extrinsic factors, that is, their environments and ecosystems. Closely related organisms tend to inhabit similar environments, and therefore exhibit similar rates of evolution. Extrinsic factors affecting evolutionary rate include climate/latitude, population size (in turn affected by factors such as available geographic range or food) and when a species exists in time.

Climate/latitude. Molecular evolution has been shown to occur faster in mammals[150], birds[151], amphibians[152], and marine fishes[153] in warm climates and low latitudes than in cool climates and high latitudes. Intriguingly, however, such an effect is not seen in squamates[154].

Geographic range. Clusters of species inhabiting small areas, e.g. islands or archipelagos, are likely to have smaller populations than lineages inhabiting large areas such as continents. In such circumstances, founder effects can promote rapid speciation and change. It has been demonstrated empirically that more nearly neutral mutations drift to fixation in small island populations[155], while substitution rates tend to be higher when effective population sizes are small[156,157].

Time period. Time may be an indirect correlate of clock rate. Many physical environmental variables (notably temperature) change over time, and to the extent that these may influence rate[150,152] , clock rates may vary through time. A significant component of this variation might therefore be expected to lie along branches (root to tips) rather than across them. For example, high temperatures in the late Cretaceous and early Cenozoic[158] might result in elevated rates of evolution in lineages at this time. It is also possible that Red Queen interactions or the biotic environment[150] may influence rates. Clades evolving in periods of high diversity (such as the Recent or latest Cretaceous) are more likely to experience intense competition and antagonistic interactions with predators and parasites. Such interactions might be expected to elevate rates relative to those experienced in the immediate wake of a mass extinction event, such as in the Paleocene.

II. Empirical Evidence

We have identified a number of theoretical reasons for preferring an autocorrelated model of molecular evolution. Ultimately, however, models must be verified or rejected on the basis of how well they describe real world observations. This is complicated by the fact that there is no absolute benchmark for accuracy, and tests are always implemented within the framework of one or other set of model assumptions. Studies of molecular data have also provided conflicting results.

A study of viral and mammalian data showed no autocorrelation in viruses, and weak but nonsignificant rate autocorrelation in the mammalian data[159]. However, the authors suggested that the signal of rate autocorrelations might increase with the size of the data set. This is because

larger data sets will tend to encompass more variation in life history parameters and other intrinsic aspects of biology, such that there might be a stronger signal of rate autocorrelation[159]. By contrast, Lepage et al.[160] evaluated three datasets, one for eukaryotes, one for vertebrates, and one for mammals. They found that two autocorrelated models, the lognormal and CIR model, outperformed uncorrelated models for all three. Yet another study simulated DNA evolution under a variety of different relaxed clock models, and then attempted to detect support for these models. It was found that rate autocorrelation was difficult to detect even with good taxon sampling[161]. This raises the possibility that previous failures to detect rate autocorrelation by some studies may result from methodological inadequacies, rather than the absence of autocorrelation *per se.*

This suspicion was borne out by a recent study[142] which used machine learning to detect autocorrelation in data sets that other methods might miss. Their study revealed the existence of extensive rate autocorrelation in both DNA and amino acid sequence data for a wide variety of taxa, including prokaryotes, protazoans, fungi, plants, insects, birds, and mammals. They found that rate autocorrelation is common across the evolutionary tree, being significant in all of the 17 data sets that they tested[142].

Conclusions

Theoretical considerations suggest that autocorrelated relaxed clock models are probably, in general, more realistic models of molecular evolution than uncorrelated relaxed clock models. Empirical work now bears this out. Accordingly, we suggest that the results of the autocorrelated molecular clock are likely to provide a more accurate estimate of divergence time for our data.

2.5. Additional output

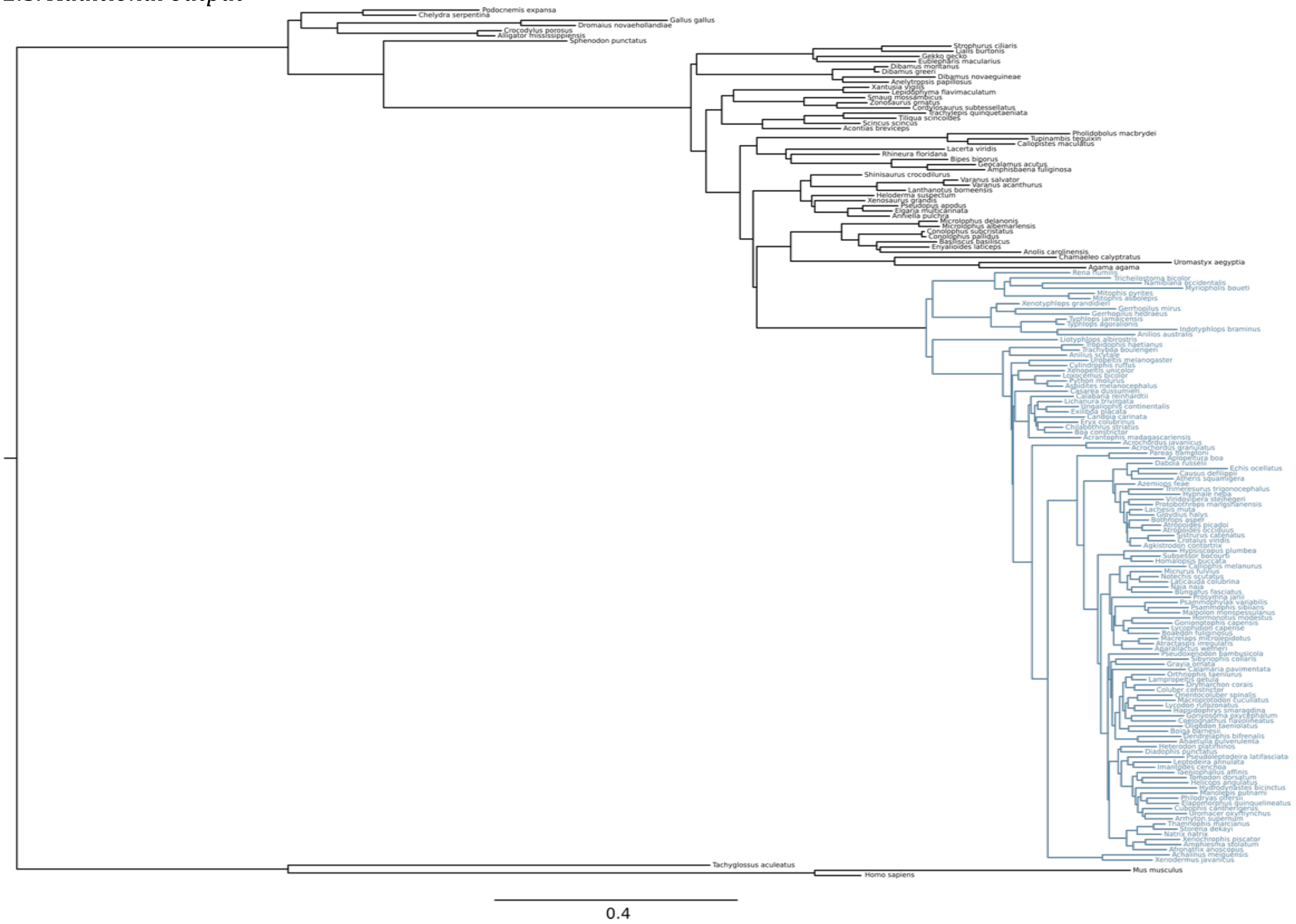

Supplementary Figure 3. Consensus tree from PhyloBayes showing branch lengths. Ingroup shown in blue.

Supplementary Table 3. Overview of parameters, models, and input data used for each molecular clock analysis. KEA – Klein et al calibration set, as set out above in SI2.1.

Name	Fig.	Clock model	Prior distribution	Calibration set	Partitions	Tree topology
Skew-T prior	S4	Correlated rates	Skew-T	KEA	12	new
Skew-Normal prior	S5	Correlated rates	Skew-Normal	KEA	12	new
Correlated-rates model	S6	Correlated rates	Uniform	KEA	12	new
Independent-rates model	S7	Independent rates	Uniform	KEA	12	new
Independent-rates model	S8	Independent rates	Skew-T	KEA	12	new
1 Partition	S9	Correlated rates	Uniform	KEA	1	new
Scolecophidia' monophyly	S10	Correlated rates	Uniform	KEA	12	new, monophyly of 'Scolecophidia' enforced
Anguimorpha + Iguania clade	S11	Correlated rates	Uniform	KEA	12	new, clade of Anguimorpha + Iguania enforced
KEA + *Parviraptor*	S12	Correlated rates	Uniform	KEA, *Parviraptor* replacing *Tetrapodophis* as calibration	12	new
KEA + *Parviraptor*	S13	Correlated rates	Skew-T	KEA, *Parviraptor* replacing *Tetrapodophis* as calibration	12	new
KEA + Simoliophiidae	S14	Correlated rates	Uniform	KEA + Simoliophiidae added	12	new
KEA + Simoliophiidae	S15	Correlated rates	Skew-T	KEA + Simoliophiidae added	12	new
KEA + *Australophis*	S16	Correlated rates	Uniform	KEA + *Australophis* added	12	new
HEA	S17	Correlated rates	Uniform	Head et al.[5,6]	12	new
HEA	S18	Correlated rates	Skew-T	Head et al.[5,6]	12	new
HEA - Simoliophiidae	S19	Correlated rates	Uniform	Head et al.[5,6], Simoliophiidae removed	12	new
HEA - *Australophis*	S20	Correlated rates	Uniform	Head et al.[5,6], *Australophis* removed	12	new
HEA - Simoliophiidae, *Australophis*	S21	Correlated rates	Uniform	Head et al.[5,6], Simoliophiidae and *Australophis* removed	12	new

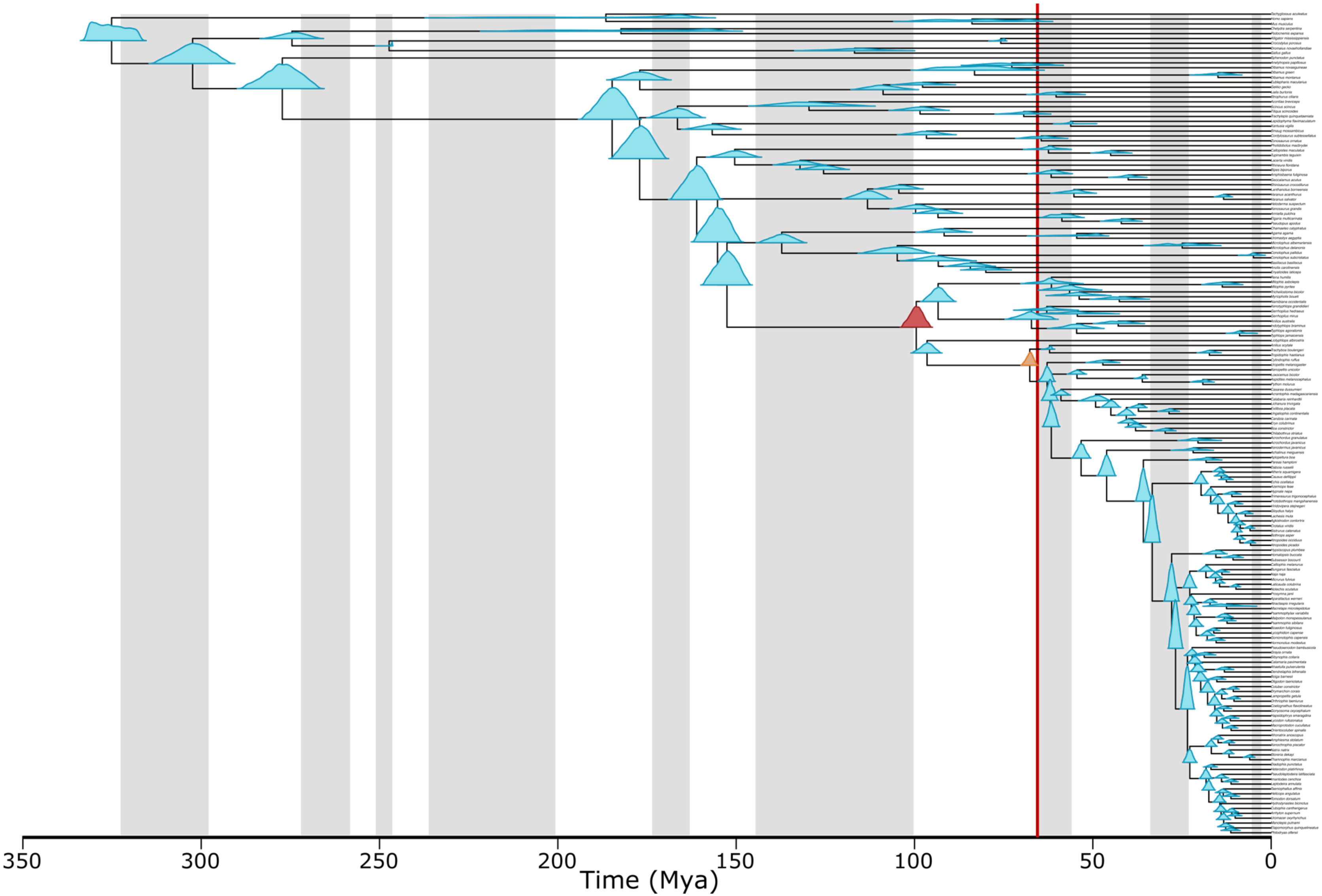

Supplementary Figure 4. Time-calibrated phylogeny based on 42 calibrations, under a Bayesian Inference framework applying the **skew-T prior**. Distribution curves represent posterior age estimates for ingroup nodes: red – crown snakes, orange – crown Alethinophidia. Red vertical bar represents K-Pg mass extinction event.

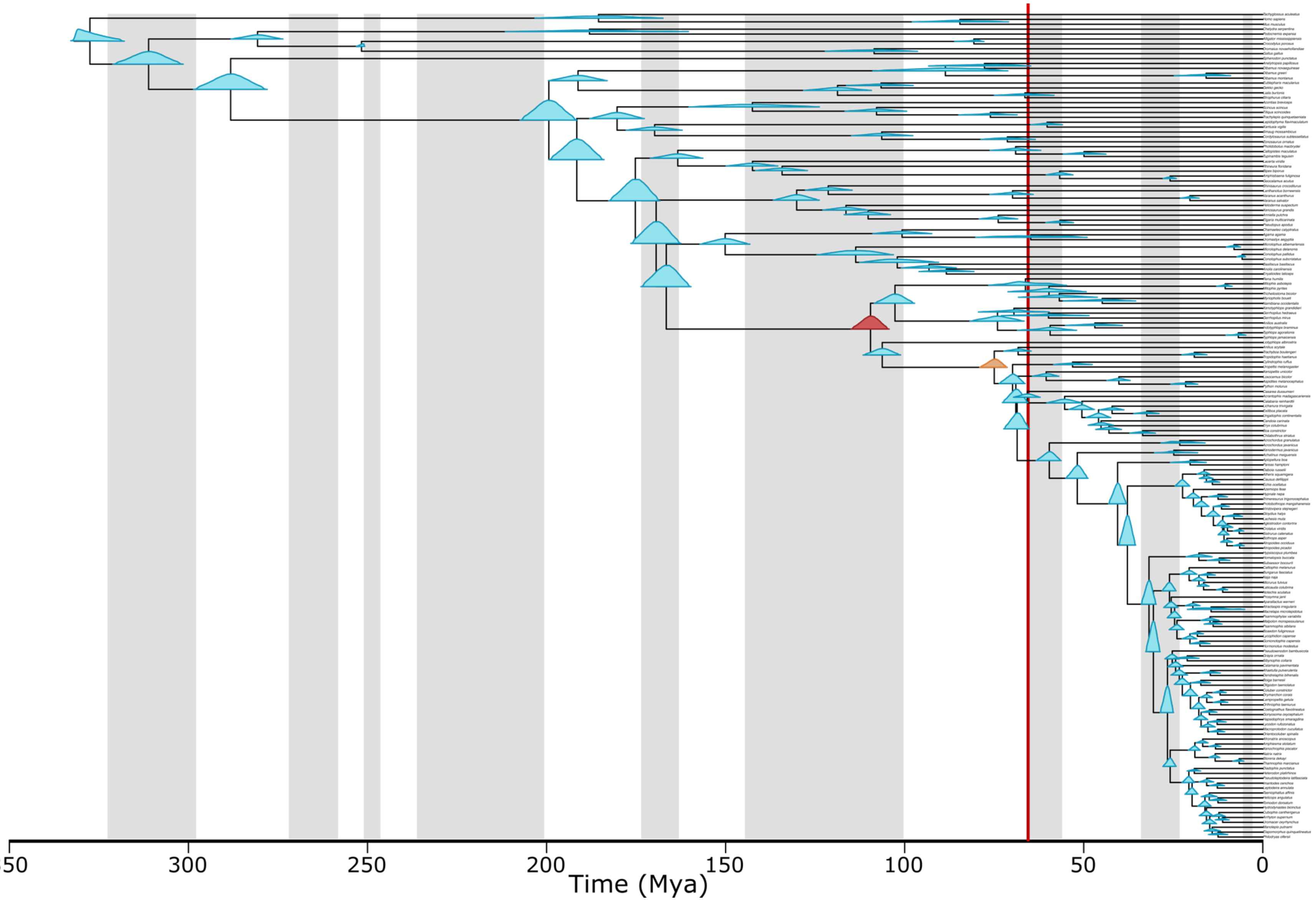

Supplementary Figure 5. Time-calibrated phylogeny based on 42 calibrations, under a Bayesian Inference framework applying the **skew-normal prior**. Distribution curves represent posterior age estimates for ingroup nodes: red – crown snakes, orange – crown Alethinophidia. Red vertical bar represents K-Pg mass extinction event.

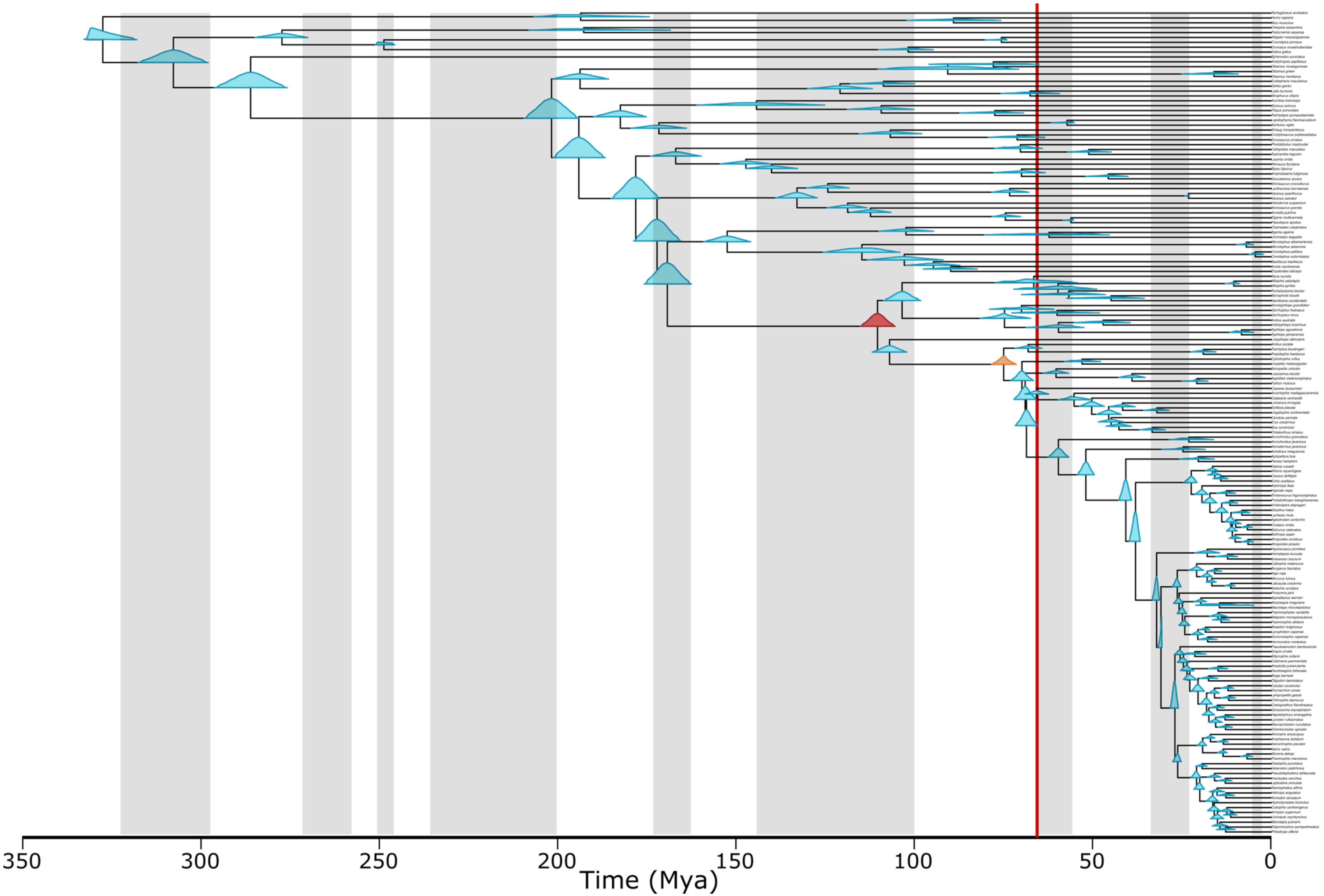

Supplementary Figure 6. Time-calibrated phylogeny based on 42 calibrations, under a Bayesian Inference framework and **uncorrelated rates model**, applying the **uniform prior**. Distribution curves represent posterior age estimates for ingroup nodes: red – crown snakes, orange – crown Alethinophidia. Red vertical bar represents K-Pg mass extinction event.

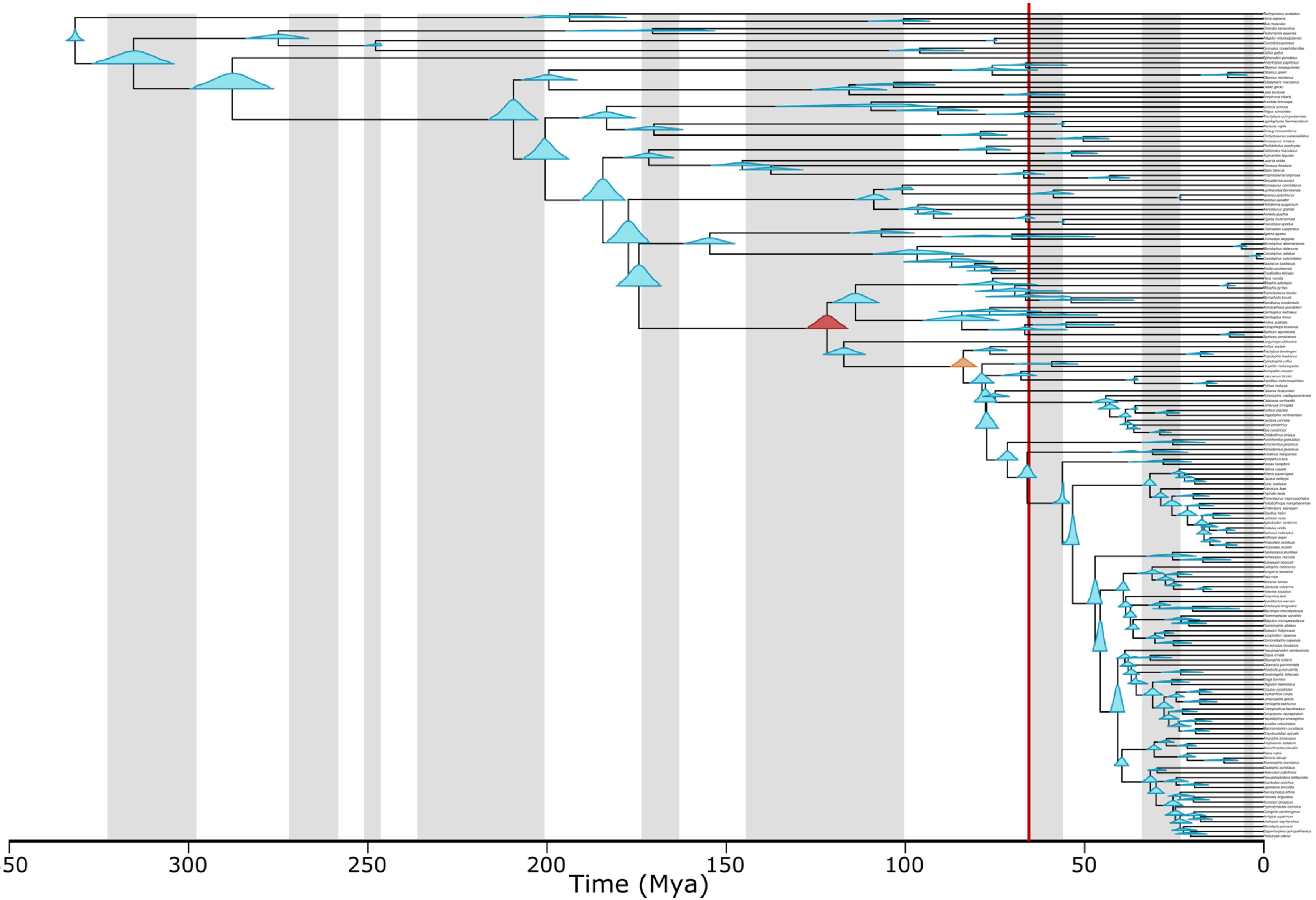

Supplementary Figure 7. Time-calibrated phylogeny based on 42 calibrations, under a Bayesian Inference framework and **correlated rates model**, applying the **uniform prior**. Distribution curves represent posterior age estimates for ingroup nodes: red – crown snakes, orange – crown Alethinophidia. Red vertical bar represents K-Pg mass extinction event.

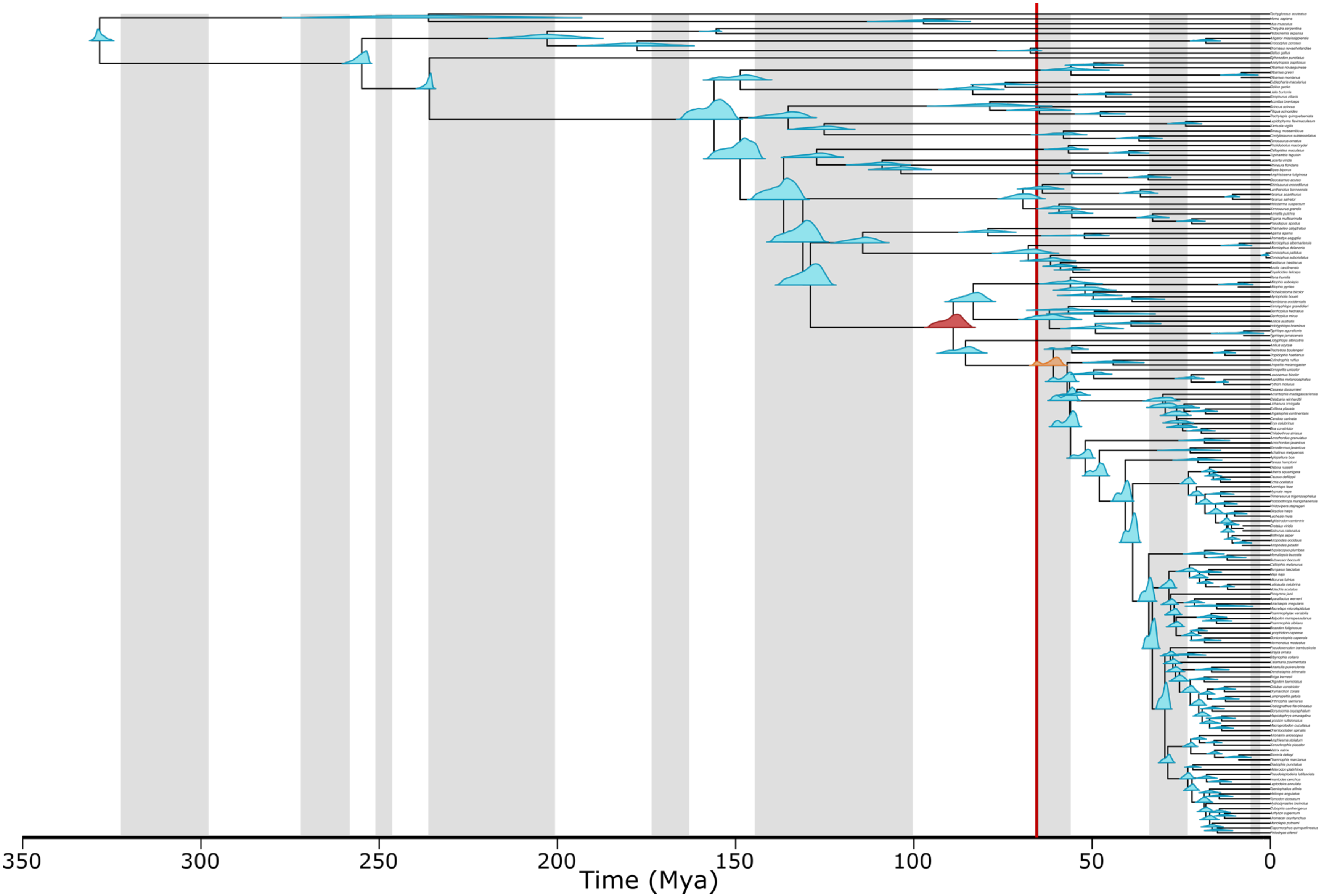

Supplementary Figure 8. Time-calibrated phylogeny based on 42 calibrations, under a Bayesian Inference framework and **correlated rates model**, applying the **skew-T prior**. Distribution curves represent posterior age estimates for ingroup nodes: red – crown snakes, orange – crown Alethinophidia. Red vertical bar represents K-Pg mass extinction event.

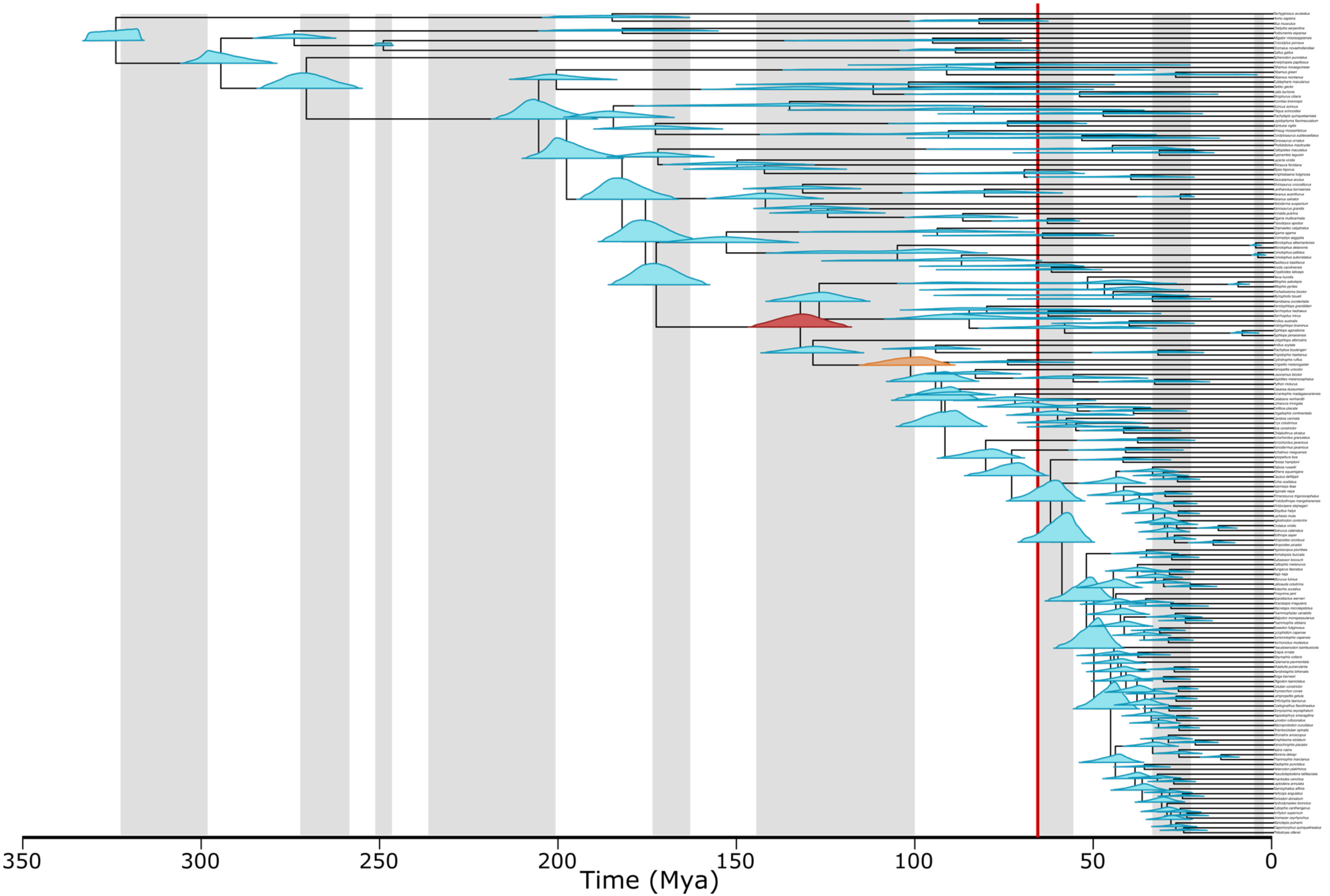

Supplementary Figure 9. Time-calibrated phylogeny based on 42 calibrations, **a single partition**, under a Bayesian Inference framework and correlated rates model, applying the uniform prior. Distribution curves represent posterior age estimates for ingroup nodes: red – crown snakes, orange – crown Alethinophidia. Red vertical bar represents K-Pg mass extinction event.

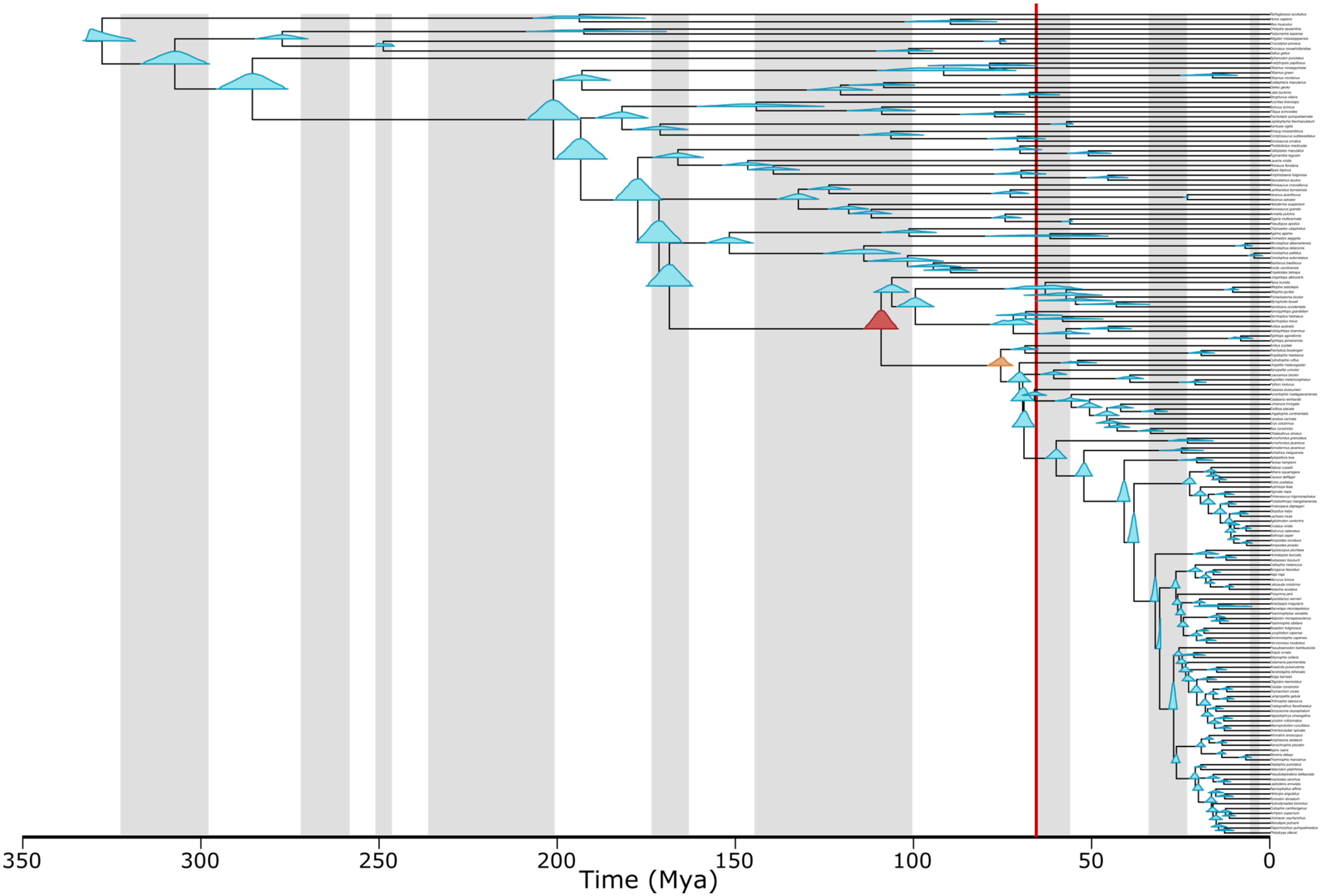

Supplementary Figure 10. Time-calibrated phylogeny based on 42 calibrations, under a Bayesian Inference framework and correlated rates model, applying the uniform prior. Phylogeny amended to recover **monophyletic 'Scolecophidia'**. Distribution curves represent posterior age estimates for ingroup nodes: red – crown snakes, orange – crown Alethinophidia. Red vertical bar represents K-Pg mass extinction event.

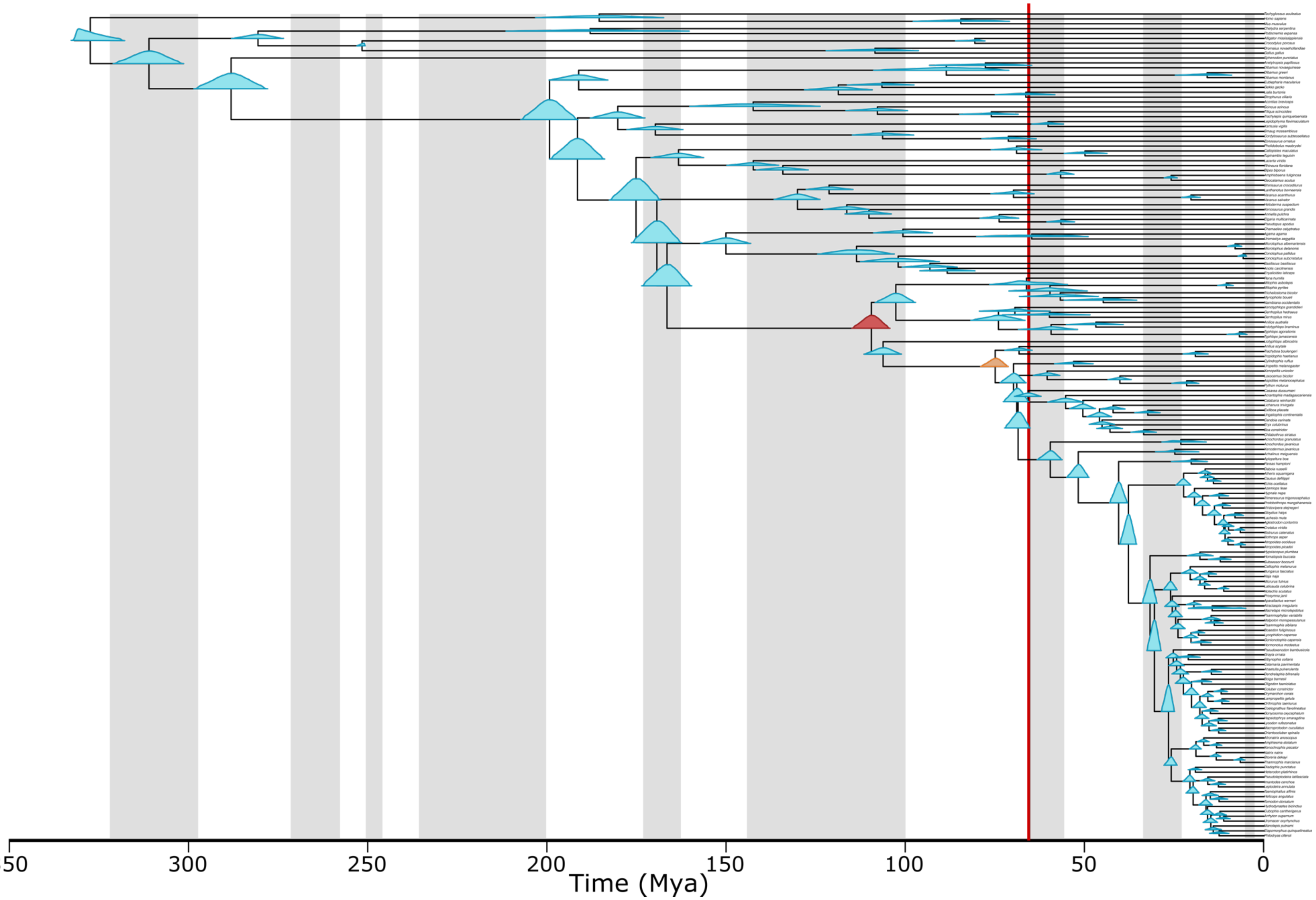

Supplementary Figure 11. Time-calibrated phylogeny based on 42 calibrations, under a Bayesian Inference framework and correlated rates model, applying the uniform prior. Phylogeny amended to recover **Anguimorpha + Iguania clade**. Distribution curves represent posterior age estimates for ingroup nodes: red – crown snakes, orange – crown Alethinophidia. Red vertical bar represents K-Pg mass extinction event.

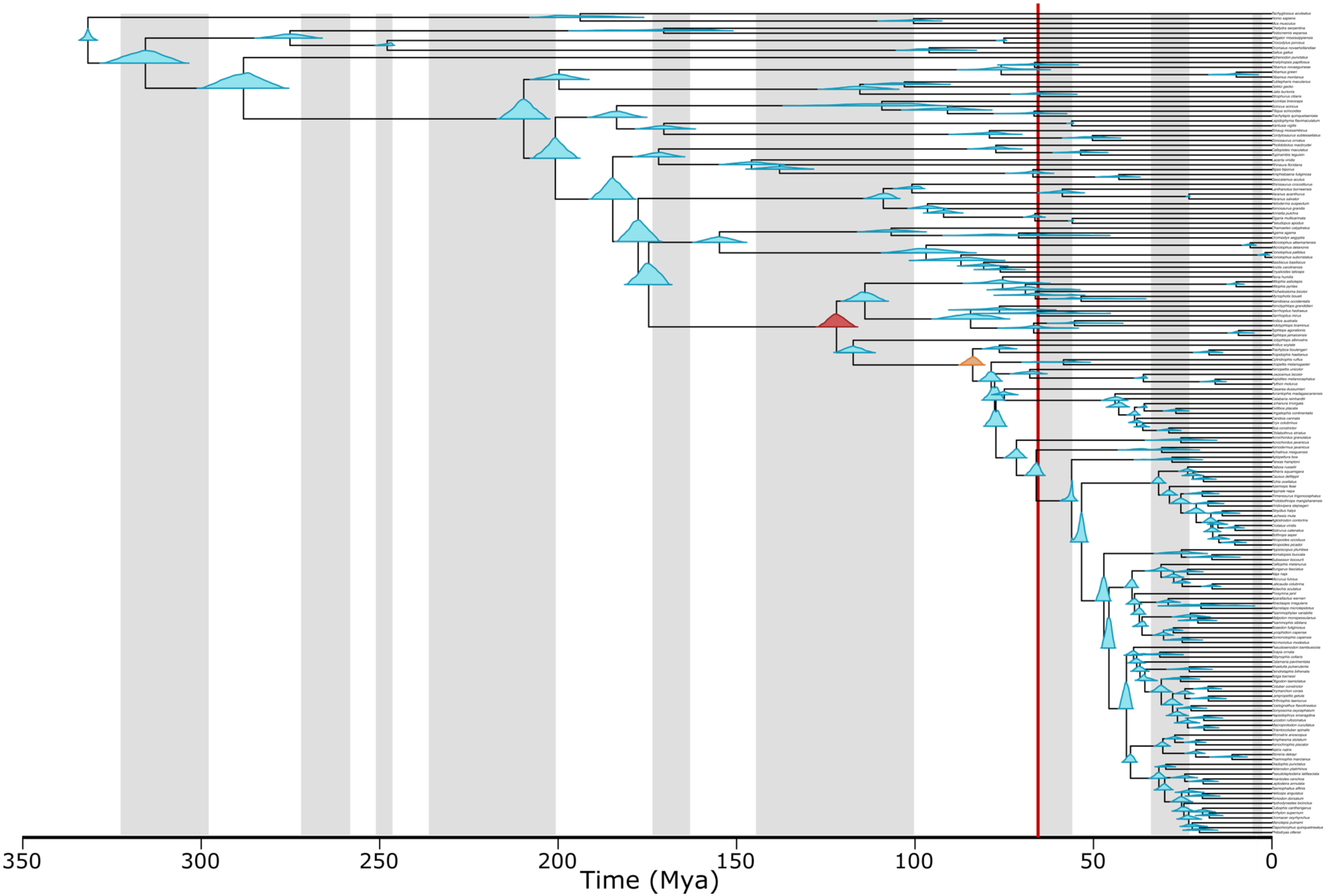

Supplementary Figure 12. Time-calibrated phylogeny based on 42 calibrations (using parviraptorids to calibrate total-group snakes), under a Bayesian Inference framework and **correlated rates model**, applying the **uniform prior**. Distribution curves represent posterior age estimates for ingroup nodes: red – crown snakes, orange – crown Alethinophidia. Red vertical bar represents K-Pg mass extinction event.

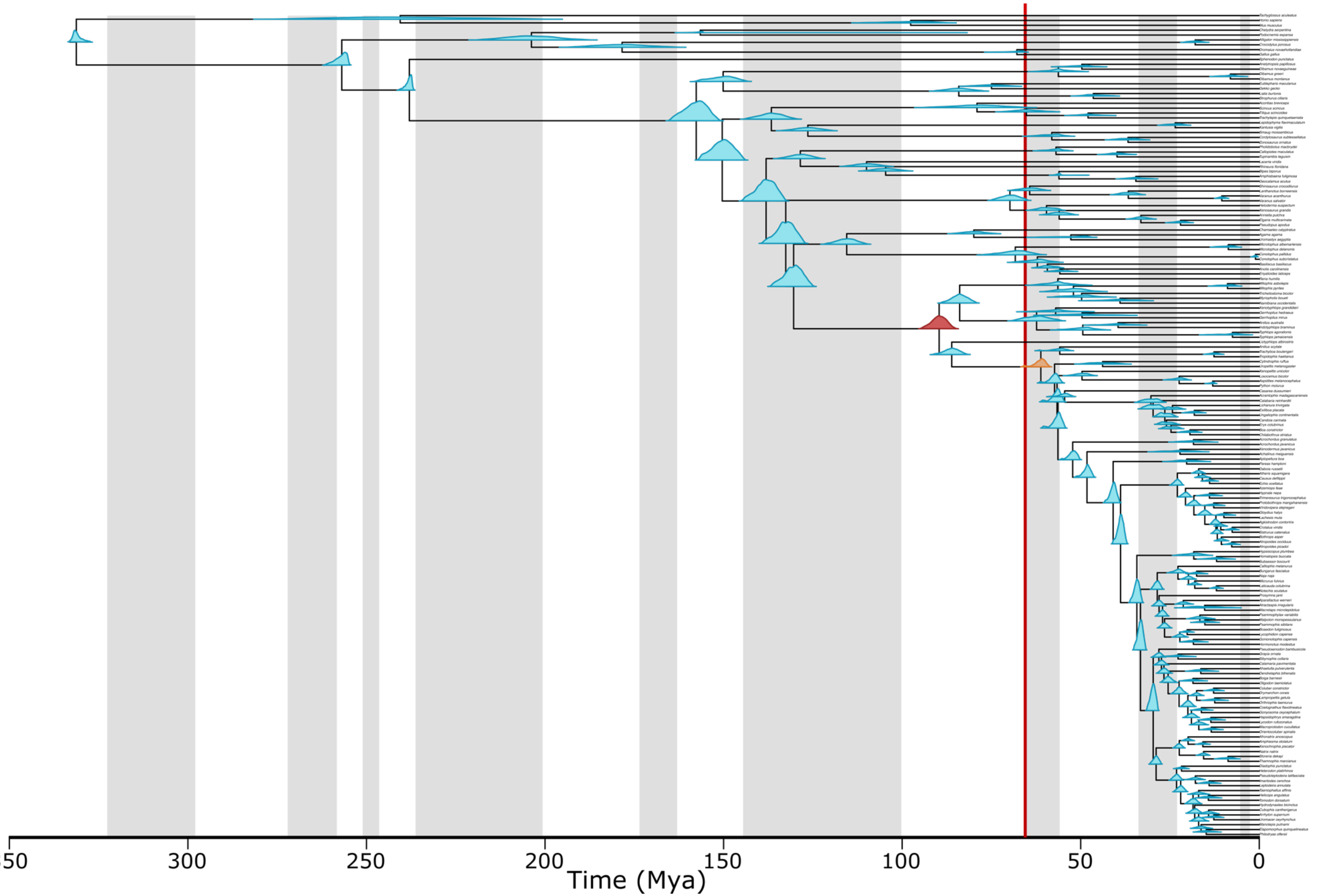

Supplementary Figure 13. Time-calibrated phylogeny based on 42 calibrations (using parviraptorids to calibrate total-group snakes), under a Bayesian Inference framework and **correlated rates model**, applying the **skew-T prior**. Distribution curves represent posterior age estimates for ingroup nodes: red – crown snakes, orange – crown Alethinophidia. Red vertical bar represents K-Pg mass extinction event.

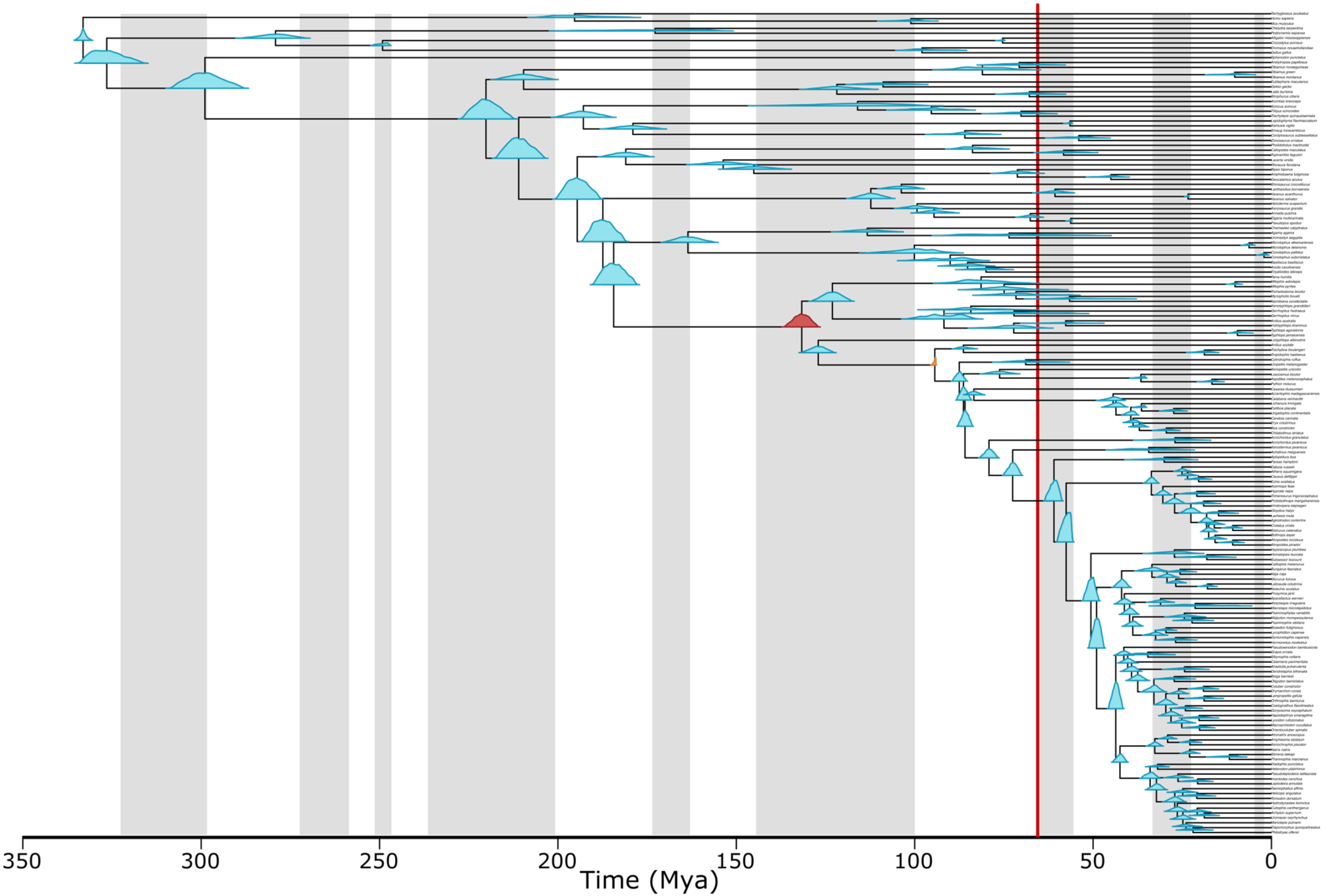

Supplementary Figure 14. Time-calibrated phylogeny based on 43 calibrations (addition of Simoliophiidae as calibration), under a Bayesian Inference framework and **correlated rates model**, applying the **uniform prior**. Distribution curves represent posterior age estimates for ingroup nodes: red – crown snakes, orange – crown Alethinophidia. Red vertical bar represents K-Pg mass extinction event.

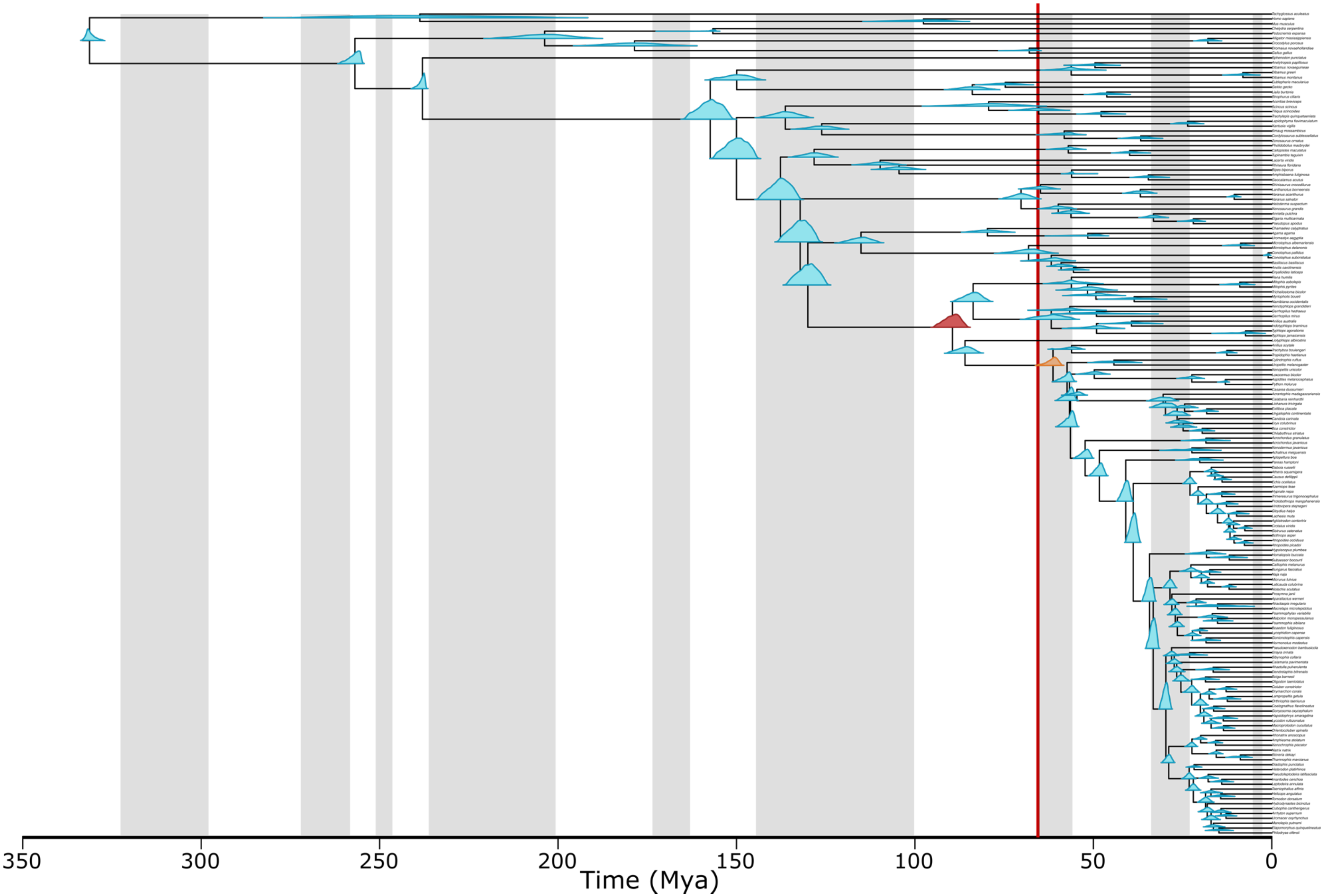

Supplementary Figure 15. Time-calibrated phylogeny based on 43 calibrations (addition of Simoliophiidae as calibration), under a Bayesian Inference framework and **correlated rates model**, applying the **skew-T prior**. Distribution curves represent posterior age estimates for ingroup nodes: red – crown snakes, orange – crown Alethinophidia. Red vertical bar represents K-Pg mass extinction event.

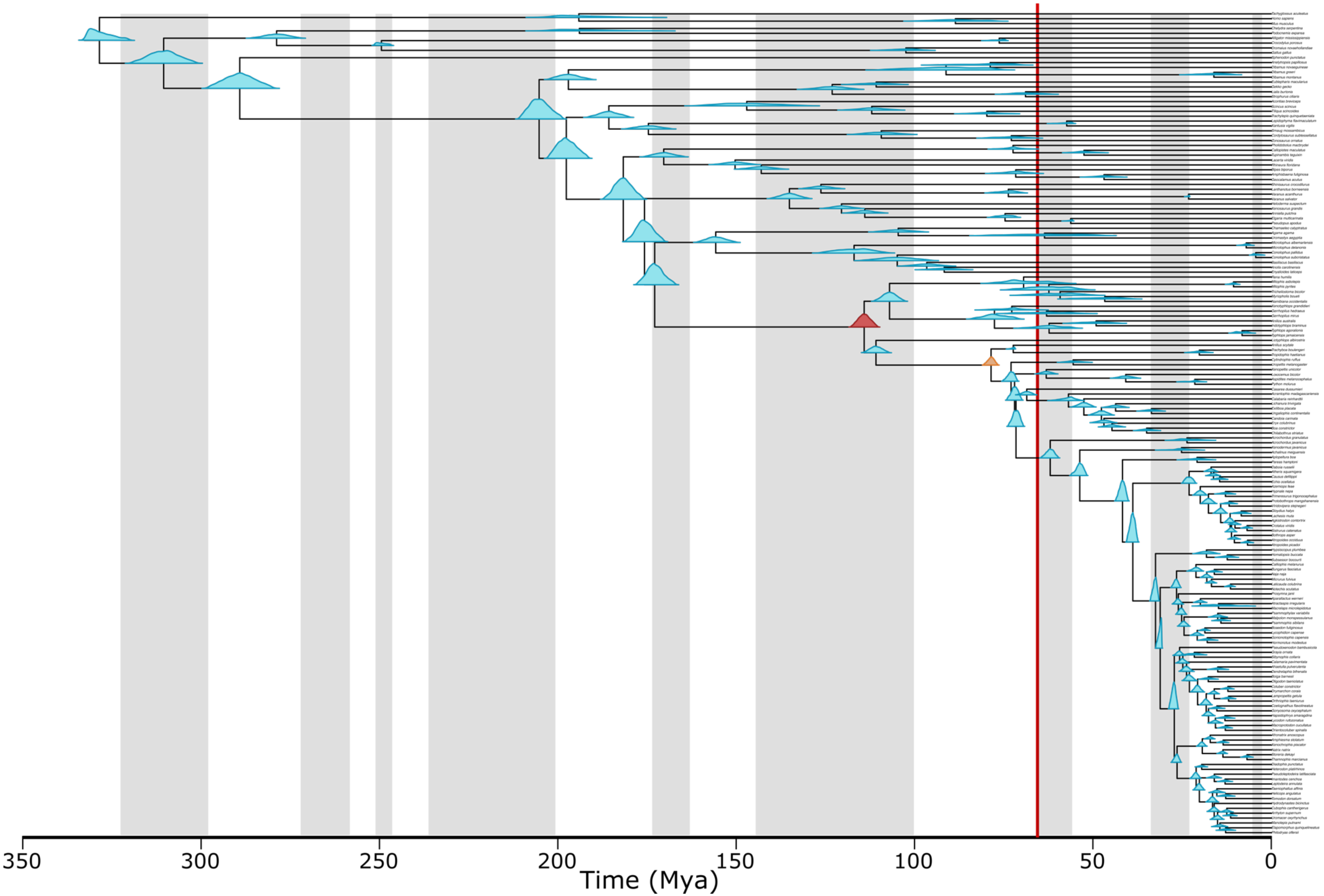

Supplementary Figure 16. Time-calibrated phylogeny based on 42 calibrations (substituting in *Australophis* as calibration), under a Bayesian Inference framework and **correlated rates model**, applying the **uniform prior**. Distribution curves represent posterior age estimates for ingroup nodes: red – crown snakes, orange – crown Alethinophidia. Red vertical bar represents K-Pg mass extinction event.

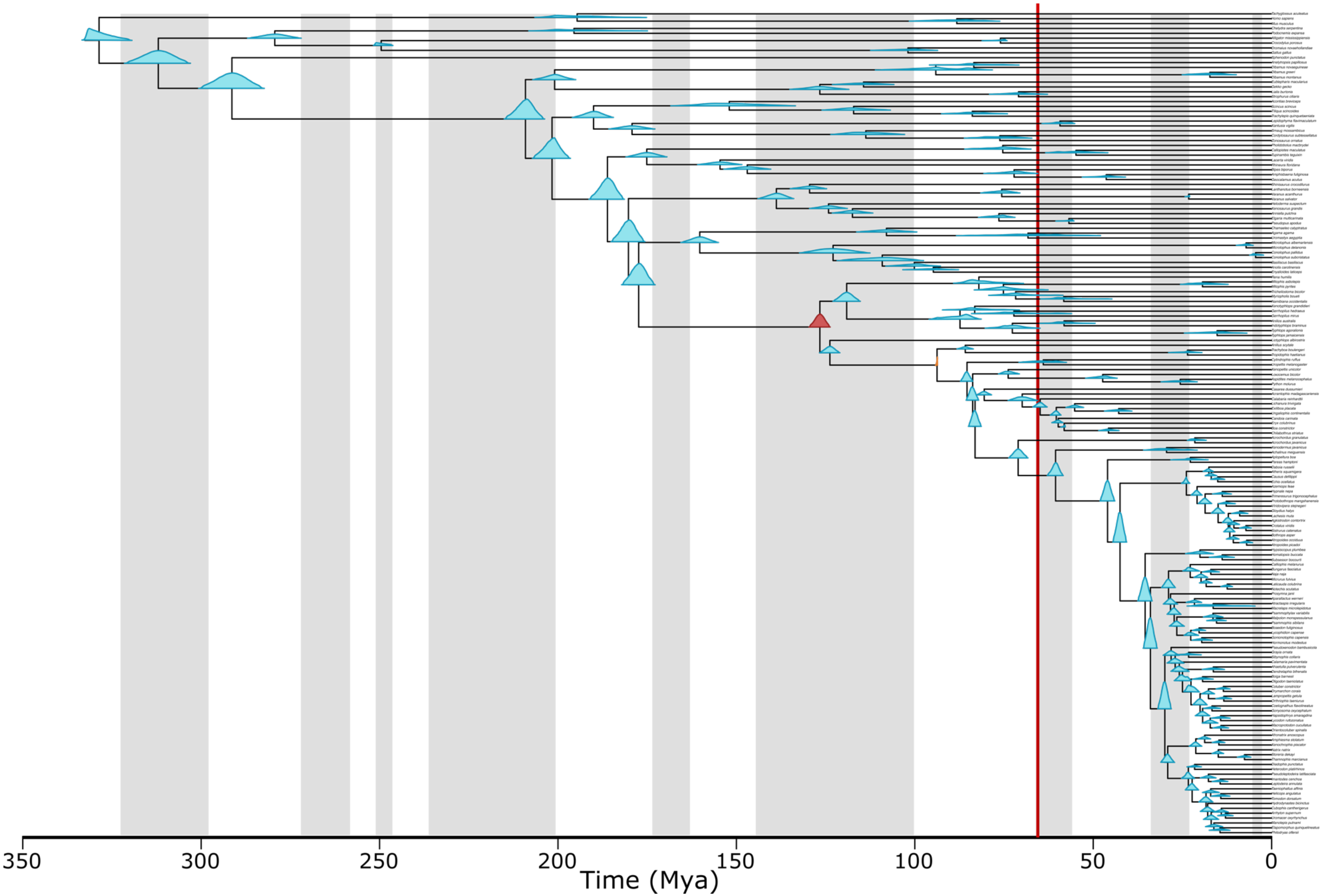

Supplementary Figure 17. Time-calibrated phylogeny based on outgroup calibrations and **Head[5] and Head et al.[6] ingroup calibrations**, under a Bayesian Inference framework and correlated rates model, applying the uniform prior. Distribution curves represent posterior age estimates for ingroup nodes: red – crown snakes, orange – crown Alethinophidia. Red vertical bar represents K-Pg mass extinction event.

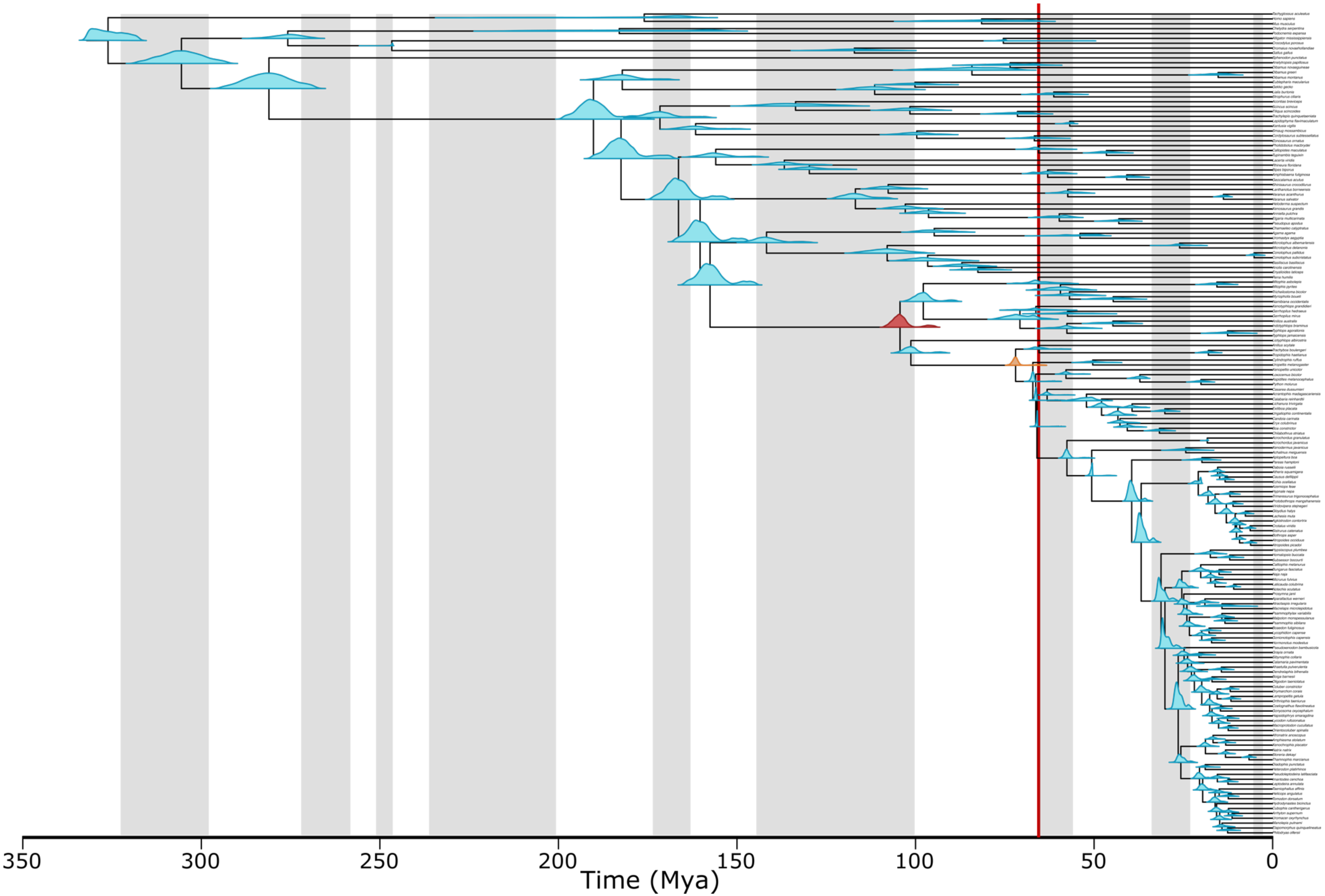

Supplementary Figure 18. Time-calibrated phylogeny based on outgroup calibrations and **Head[5] and Head et al.[6] ingroup calibrations**, under a Bayesian Inference framework and correlated rates model, applying the skew-T prior. Distribution curves represent posterior age estimates for ingroup nodes: red – crown snakes, orange – crown Alethinophidia. Red vertical bar represents K-Pg mass extinction event.

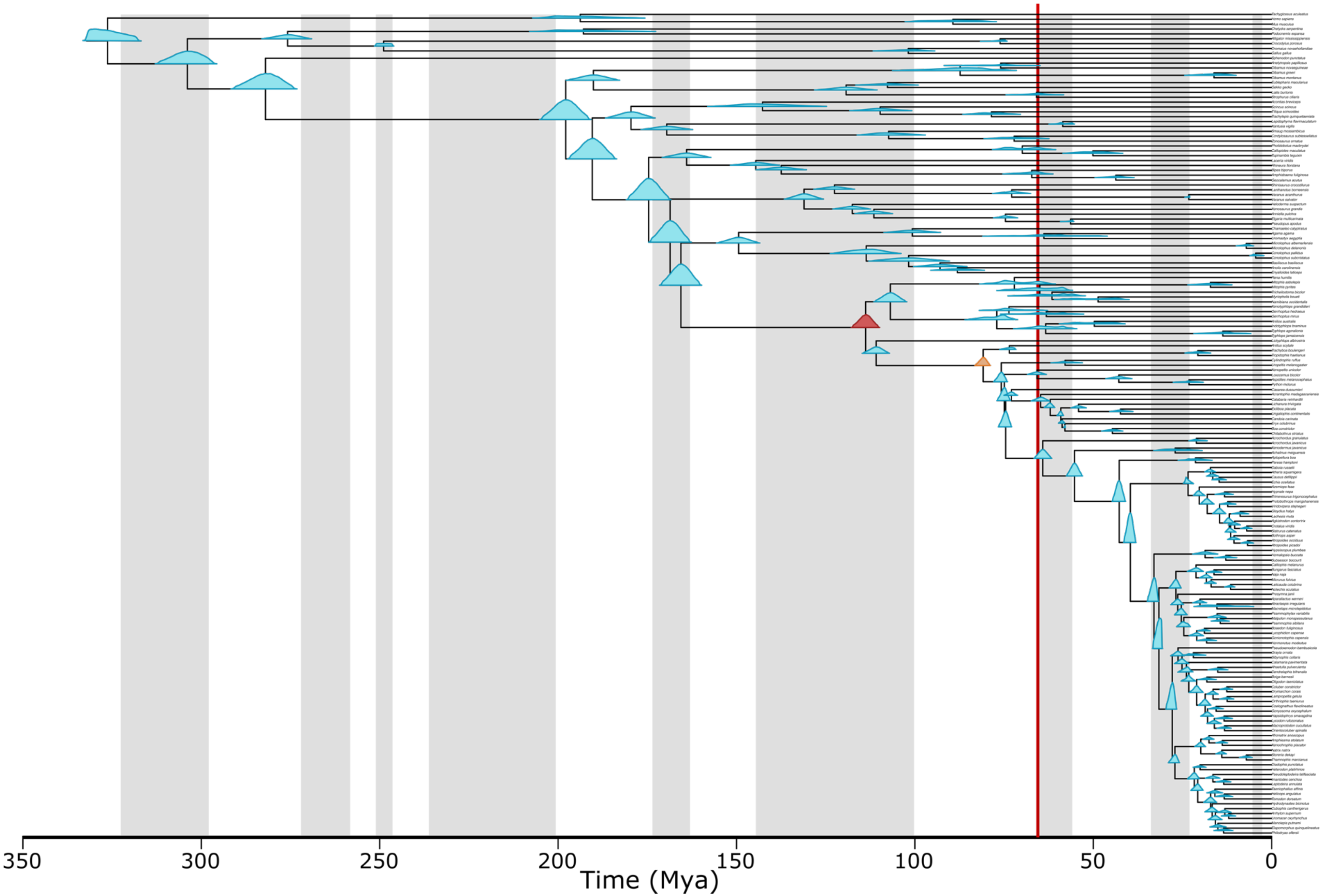

Supplementary Figure 19. Time-calibrated phylogeny based on outgroup calibrations and **Head[5] and Head et al.[6] ingroup calibrations** (Simoliophiidae - calibration for crown Alethinophidia - removed), under a Bayesian Inference framework and correlated rates model, applying the uniform prior. Distribution curves represent posterior age estimates for ingroup nodes: red – crown snakes, orange – crown Alethinophidia. Red vertical bar represents K-Pg mass extinction event.

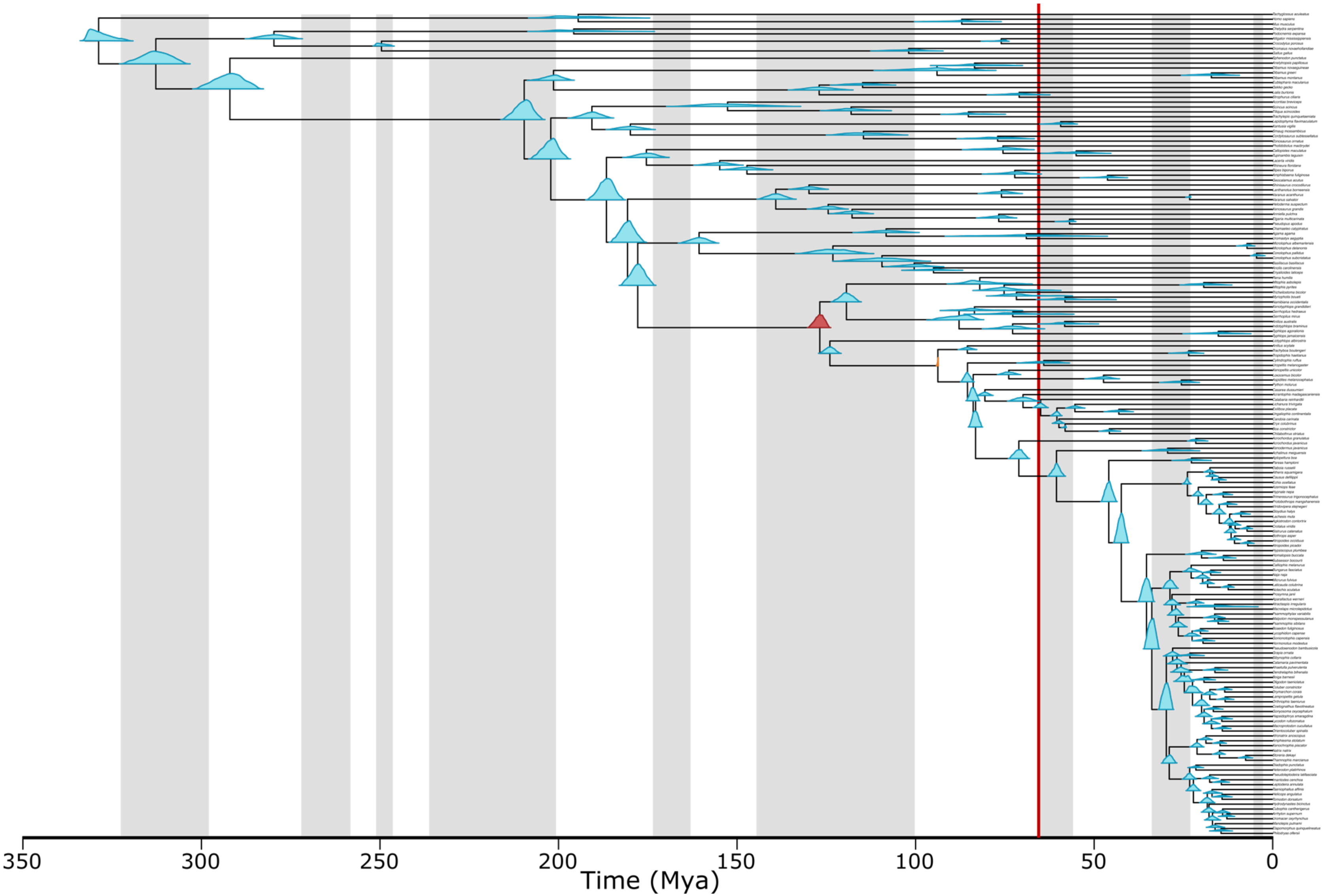

Supplementary Figure 20. Time-calibrated phylogeny based on outgroup calibrations and **Head[5] and Head et al.[6] ingroup calibrations** (*Australophis* calibration removed), under a Bayesian Inference framework and correlated rates model, applying the uniform prior. Distribution curves represent posterior age estimates for ingroup nodes: red – crown snakes, orange – crown Alethinophidia. Red vertical bar represents K-Pg mass extinction event.

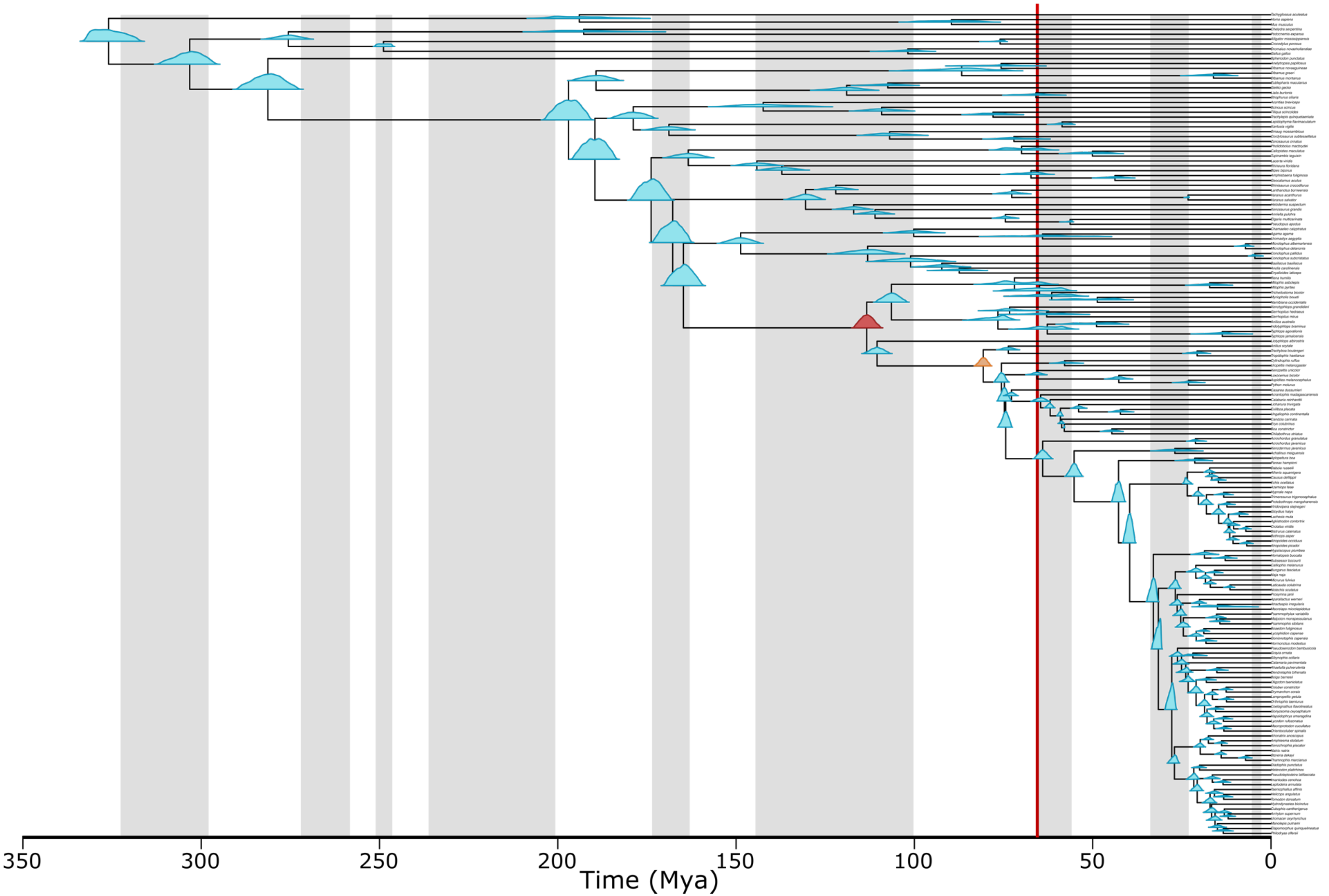

Supplementary Figure 21. Time-calibrated phylogeny based on outgroup calibrations and **Head[5] and Head et al.[6] ingroup calibrations** (Simoliophiidae and *Australophis* calibrations removed), under a Bayesian Inference framework and correlated rates model, applying the uniform prior. Distribution curves represent posterior age estimates for ingroup nodes: red – crown snakes, orange – crown Alethinophidia. Red vertical bar represents K-Pg mass extinction event.

Supplementary Information 3: Disparity and Size Through Time

3.1 Principal Component Analysis

After the data was collected and vertebral measurements were corrected for size, analyses were run in PAST. The input file can be found at SI6.

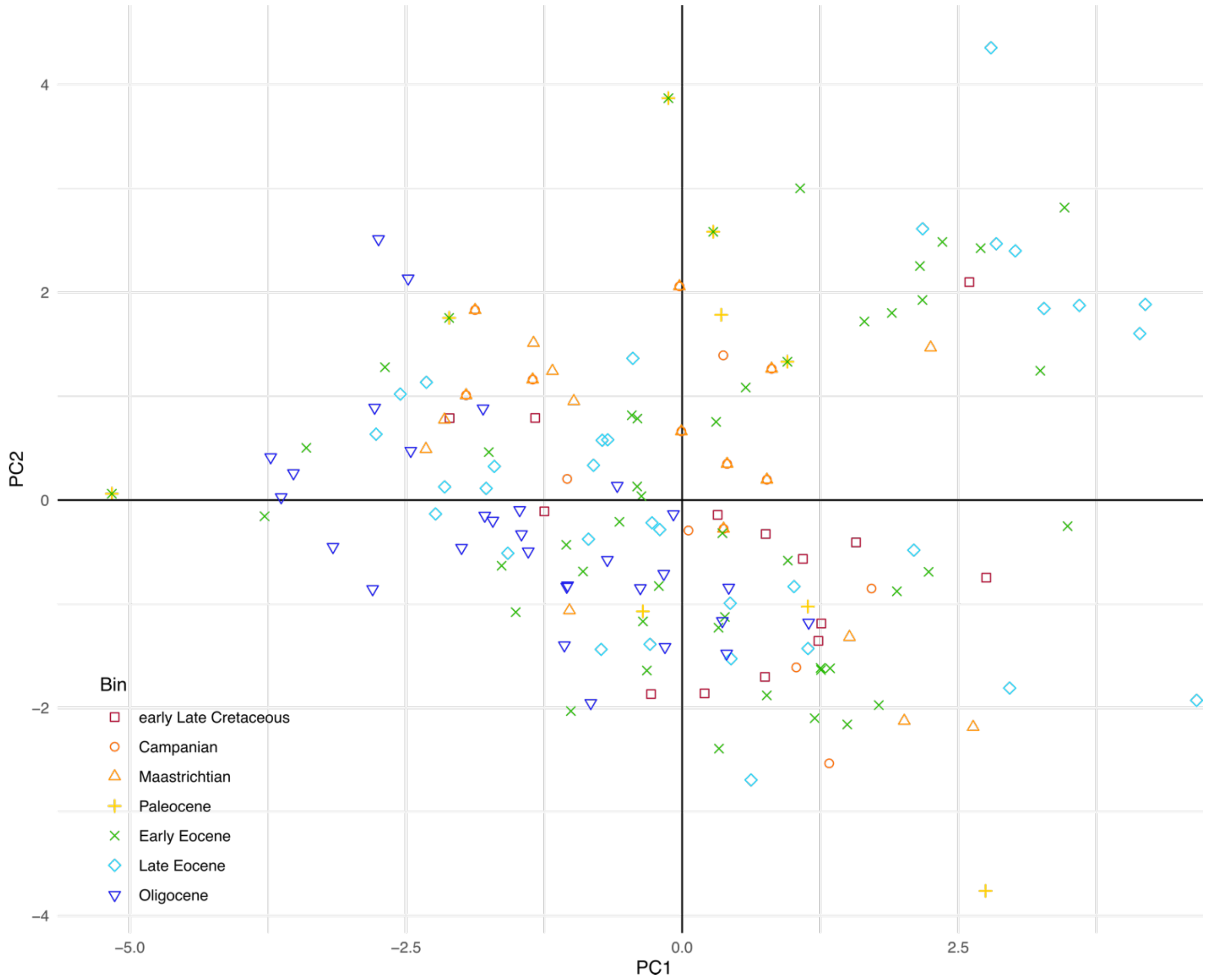

Supplementary Figure 22. Morphospace from the PCA representing principal component axes 1 and 2, over all time bins.

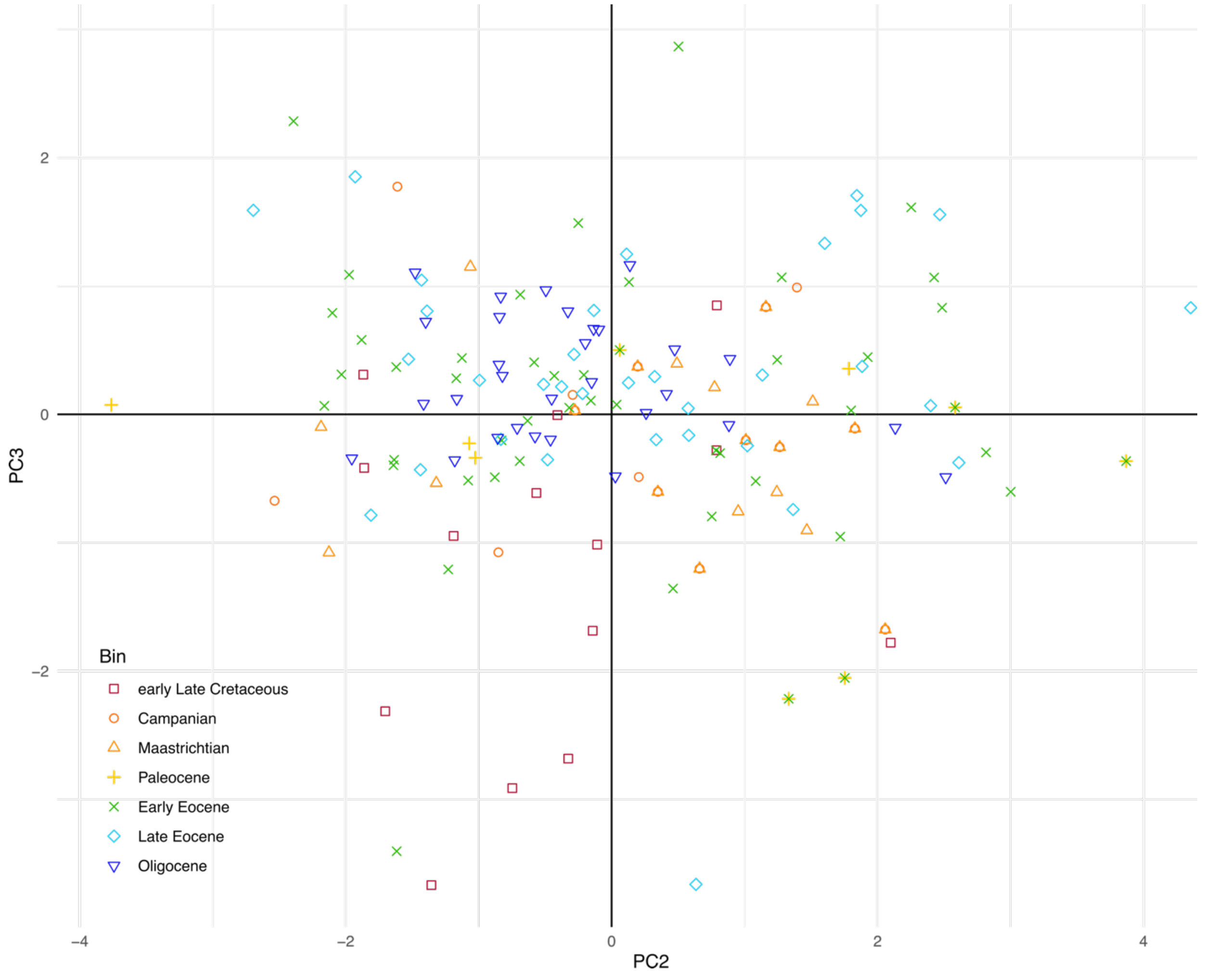

Supplementary Figure 23. Morphospace from the PCA representing principal component axes 2 and 3, over all time bins.

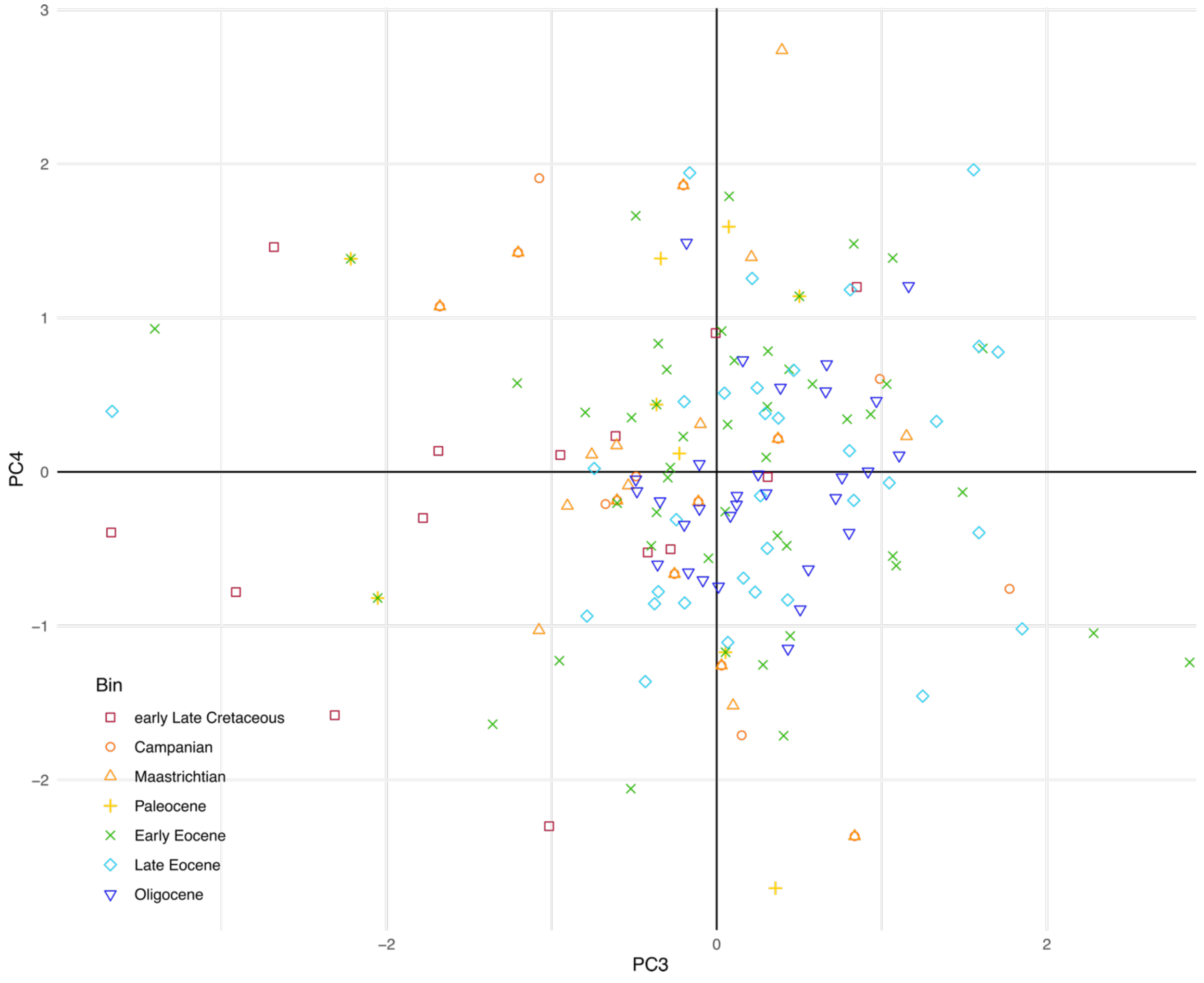

Supplementary Figure 24. Morphospace from the PCA representing principal component axes 3 and 4, over all time bins.

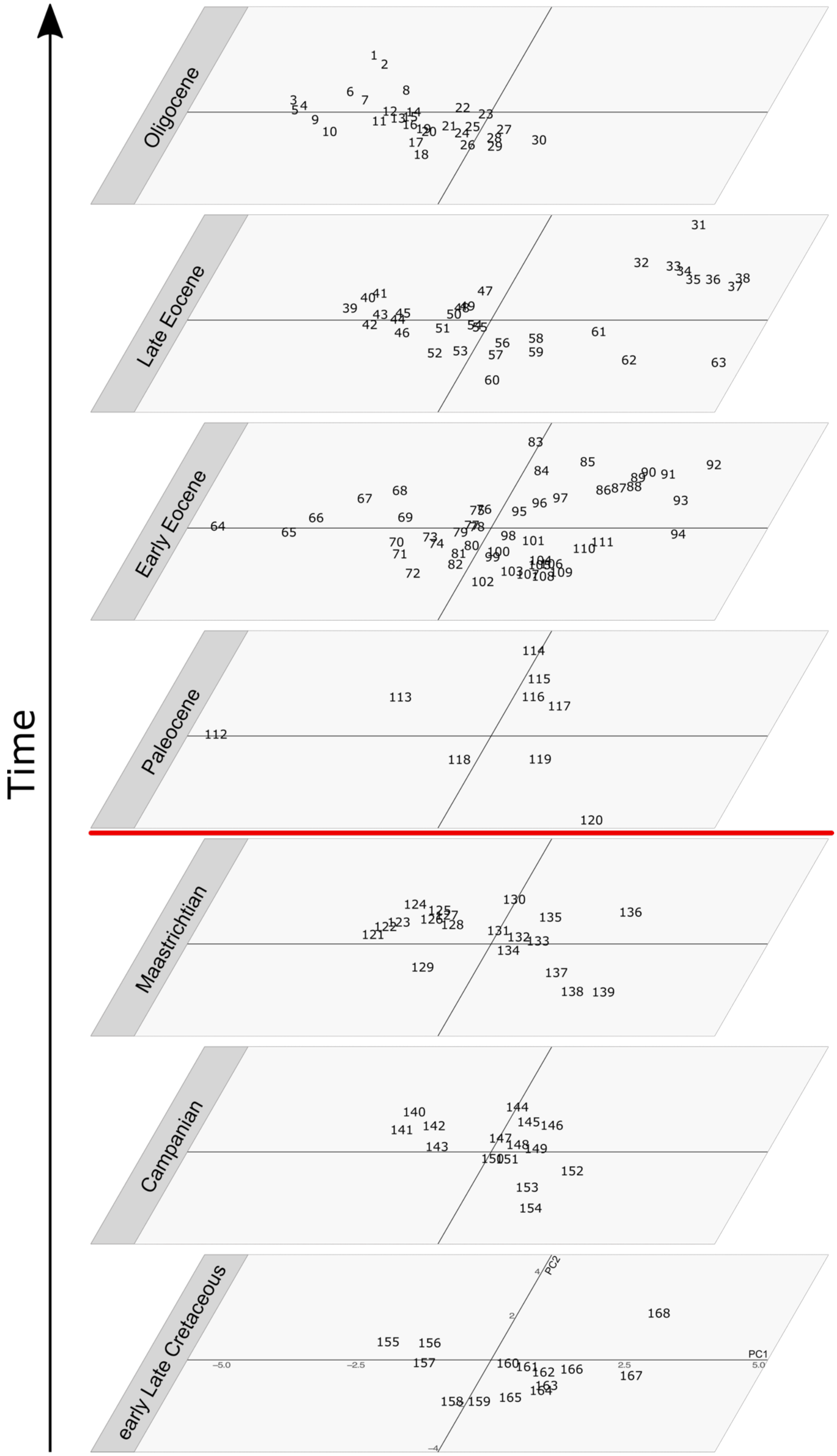

Supplementary Figure 25. Figure 2, with taxa labelled:

<u>Oligocene:</u> 1. Scolecophidia indet.; 2. *Nebraskophis oligocenicus;* 3. *Floridophis auffenbergi;* 4. *Texasophid bohemiacus;* 5. *Texasophis hecki;* 6. Colubroid morphotype B; 7. *Coluber cadurci;* 8. *Bransateryx* sp.; 9. Booid morphotype C; 10. *Rukwanyoka holmani;* 11. *Eoanilius;* 12. *Eoanilius oligocenicus;* 13. *Platyspondylia* sp.; 14. *Anilioides nebraskensis;* 15. *Eoanilius* aff. *Oligocenicus;* 16. *Platyspondylia germanica;* 17. *Calamagras* sp.; 18. *Conantophis alachuaensis;* 19. *Platyspondylia lepta* (AV); 20. *Rottophis atavus;* 21. *Falseryx neervelpensis;* 22. *Congerophis lego;* 23. Boinae D; 24. *Bavarioboa* sp.; 25. *Bransateryx vireti* (AV); 26. *Bavarioboa crocheti* (AV); 27. *Bavarioboa vaylatsae;* 28. *Bavarioboa herrlingensis* (AV); 29. Boinae B; 30. cf. *Bavarioboa* sp.
<u>Late Eocene:</u> 31. *Pterosphenus kutchensis;* 32. *Palaeophis nessovi* (AV); 33. *Palaeophis virginianus;* 34. *Palaeophis udovichenkoi;* 35. *Pterosphenus biswasi;* 36. *Pterosphenus* sp.; 37. *Pterosphenus schweinfurthi* (AV); 38. *Pterosphenus schucherti;* 39. Colubrid indet.; 40. *Coniophis* sp.; 41. *Coniophis* sp.; 42. *Platyspondylia sudrei;* 43. *Vectophis wardi;* 44. *Dunnophis cadurcensis;* 45. *Paraplatyspondylia batesi;* 46. *Renenutet enmerwer;* 47. Tropidophiid snake; 48. *Calamagras weigeli* (AV); 49. *Ogmophis compactus;* 50. *Totlandophis thomasae*; 51. Colubroid indet.; 52. *Huberophis georgiensis*; 53. Booid B; 54. Russellophiid snake; 55. cf. *Calamagras;* 56. Booid A; 57. Alethinophidia indet.; 58. *Paleryx rhombifer* (AV); 59. *Palaeopython* aff. *Cadurcensis;* 60. *Palaeopython cadurcensis;* 61. *Gigantophis garstini* (AV); 62. Boinae indet.; 63. *Gigantophis garstini* C
Early Eocene: 64. Scolecophidia indet.; 65. *Thaumastophis missiaeni;* 66. *Russellophis tenuis;* 67. *Dunnophis* cf. *matronensis;* 68. cf. *Dunnophis;* 69. *Russellophis crassus;* 70. *Cheilophis huerfanoensis* B; 71. *Szyndlaria aureomontensis;* 72. *Hechtophis ausrrinus;* 73. *Hoffstetterella brasiliensis*; 74. *Paulacoutophis perplexus;* 75. *Patagoniophis australiensis* (AV); 76. *Patagoniophis* sp. cf. *P. parvus* (AV); 77. *Calamagras gallicus* (AV); 78. *Waincophis australis;* 79. Boinae indet.; 80. *Calamagras turkestanicus;* 81. Booid A cf. *Dunnophis;* 82. *Alamitophis tingamarra;* 83. *Palaeophis* sp.; 84. *Palaeophis* sp.; 85. ?*Palaeophis toliapicus;* 86. *Woutersophis novus;* 87. *Palaeophis maghrebianus* B; 88. *Palaeophis nessovi* B; 89. *Palaeophis ferganicus;* 90. *Palaeophis* sp.; 91. *Palaeophis* sp.; 92. *Palaeophis typhaeus;* 93. *Palaeophis maghrebianus;* 94. *Palaeophis colossaeus* (AV); 95. *Alamitophis* sp. cf. *A. argentines;* 96. *Anomalophis bolcensis;* 97. *Tuscahomaeophis leggetti* (AV); 98. *Itaboraiophis depressus;* 99. *Tallahattaophis dunni;* 100. Boinae indet.; 101. *Paraungaliophis pricei;* 102. *Corallus priscus;* 103. *Waincophis cameratus*; 104. *Sanjuanophis froehlichi;* 105. *Madtsoia camposi;* 106. Boinae A; 107. *Waincophis pressulus;* 108. Boinae B; 109. *Palaeopython fischeri;* 110. Madtsoiidae indet.; 111. ?*Boa* sp.
<u>Paleocene:</u> 112. Scolecophidia indet.; 113. cf. *Dunnophis;* 114. *Palaeophis* sp.; 115. *Palaeophis* sp.; 116. *Nigerophis mirus*; 117. *Tuscahomaophis leggetti* (AV); 118. Boid; 119. *Gigantophis* sp.; 120. *Titanoboa cerrejonensis*
Maastrichtian: 121. ?*Cholophidia;* 122. *Coniophis* sp.; 123. *Nubianophis afaahus;* 124. *Coniophis dabiebus;* 125. *Indophis fanambinana;* 126. *Krebsophis thobanus;* 127. *Indophis sahnii;* 128. *Coniophis precedens;* 129. *Nidophis insularis;* 130. Serpentes incertae sedis; 131. *Alamitophis argentines;* 132. Lapparentophiid-grade snake A; 133. *Patagoniophis parvus;* 134. *Autralophis anilioides;* 135. *Alamitophis elongatus;* 136. *Palaeophis* sp.; 137. *Adinophis fisaka;* 138. *Menara nosymena;* 139. *Madtsoia madagascariensis*

Campanian: 140. *Coniophis dabiebus*
141. *Nubianophis afaahus;* 142. *Krebsophis thobanus;* 143. *Coniophis* sp.; 144. Serpentes incertae sedis; 145. *Coniophis* sp.; 146. *Alamitophis elongatus;* 147. *Alamitophis argentines;* 148. Lapparentophiid-grade snake A; 149. *Patagoniophis parvus*; 150. *Herensugea caristiorum*; 151. *Australophis anilioides*; 152. *Coniophis cosgriffi;* 153. *Dinilysia patagonica;* 154. *Menara laurasiae*
early Late Cretaceous: 155. *Coniophis* sp.; 156. *Coniophis* sp.; 157. *Lunaophis aquaticus*; 158. *Najash rionegrina;* 159. *Dinilysia patagonica;* 160. *Podophis descouensis*; 61. cf. *Seismophis septentrionalis;* 162. *Norisophis begaa;* 163. *Norisophis* sp.; 164. *Pouitella pervetus;* 165. *Simoliophis* cf. *S. libycus;* 166. *Madtsoia* aff. *Madagascariensis;* 167. *Simoliophis rochebrunei;* 168. *Lapparentophis defrennei*

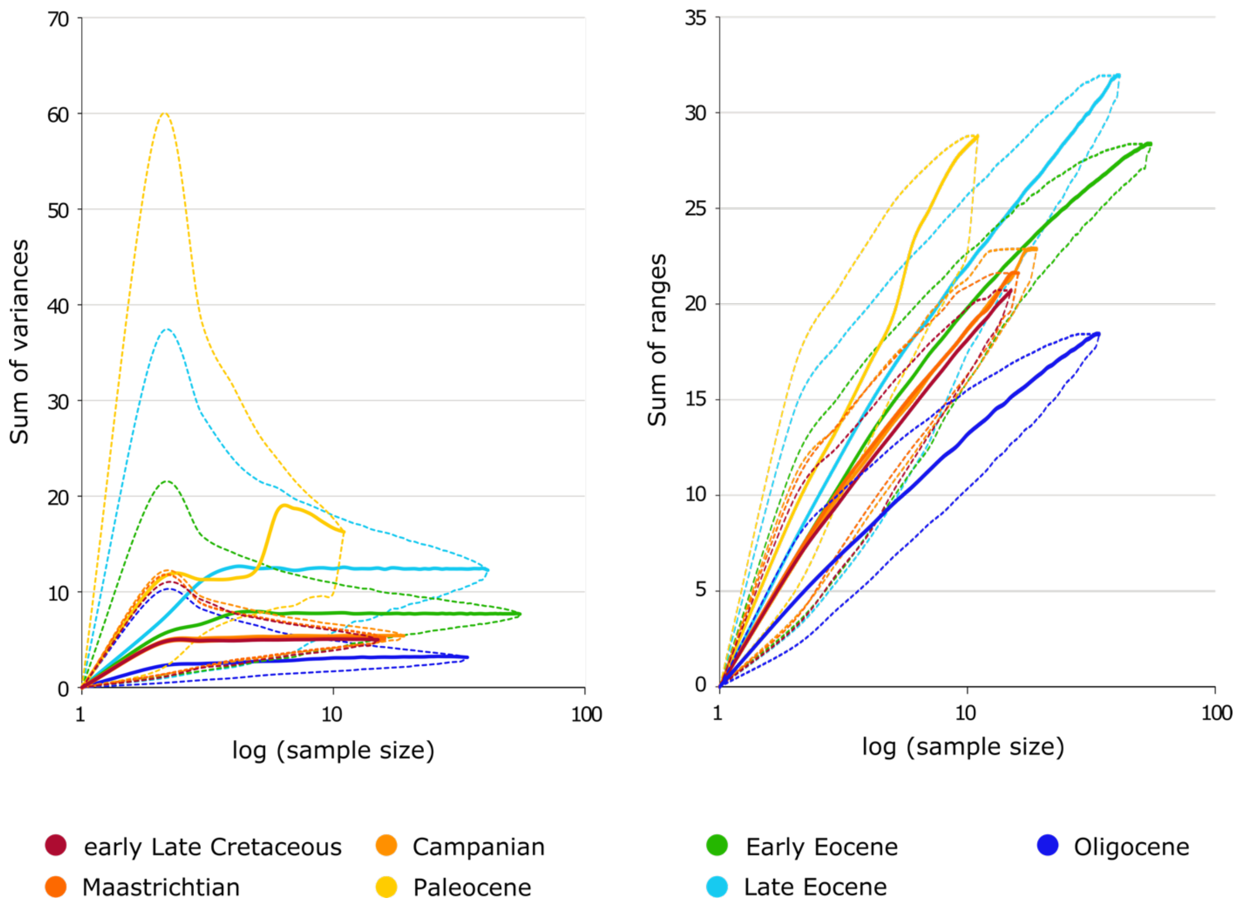

Supplementary Figure 26. Sums of ranges and variances for principal component analyses. Curves show median (solid lines), with upper and lower 95th centiles (dotted lines) from 10,000 jackknife resamplings of vertebrae.

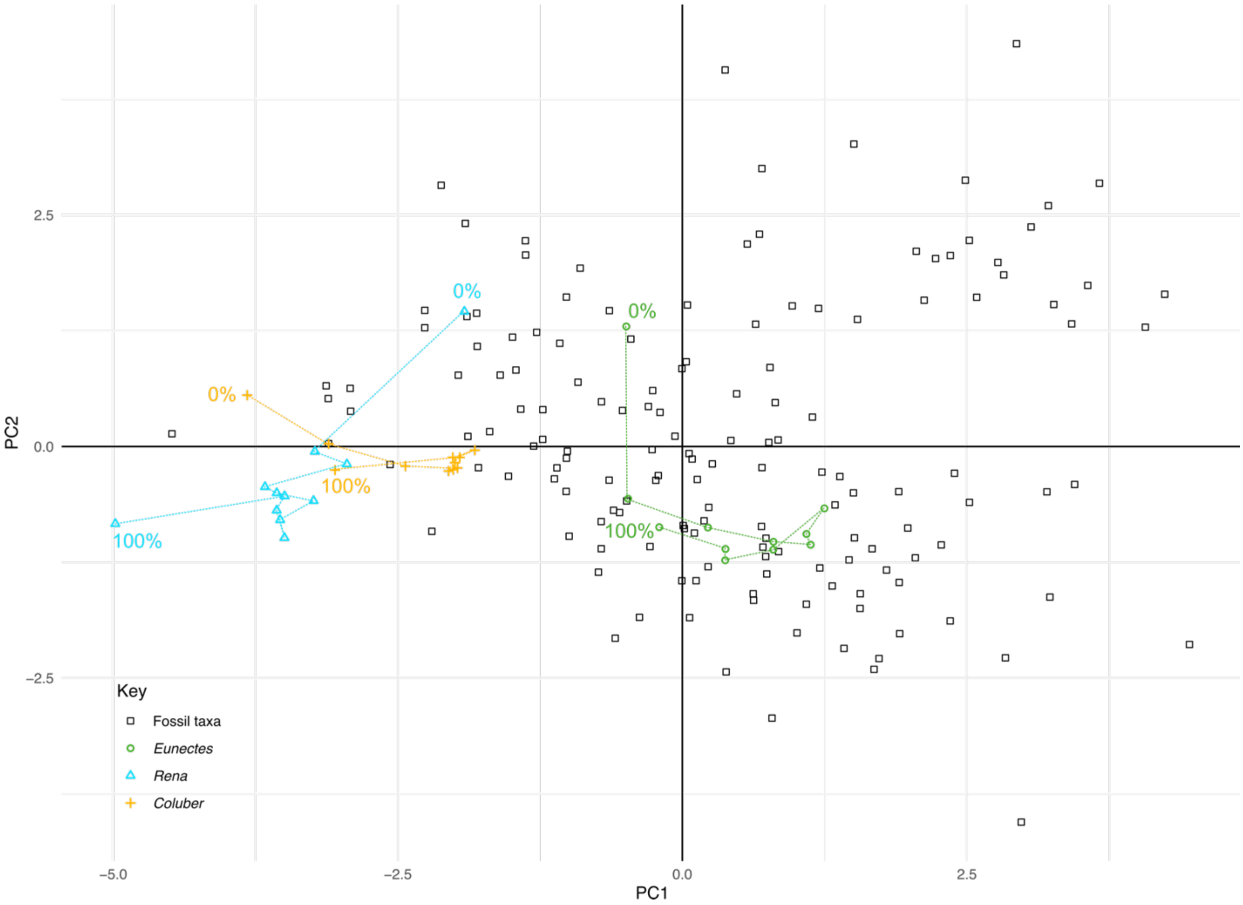

Supplementary Figure 27. Morphospace from the PCA including 3 extant taxa, *Eunectes, Coluber,* and *Rena*. Data points for extant taxa represent morphometric measurements taken every 10% along the precloacal vertebral column as per Head & Polly[162,163]. Lines follow along length of vertebral column from 0% to 100% for extant taxa.

The aim for this study was to include only mid-trunk vertebrae in the analysis. As many of the taxa included are represented by just a small number of specimens, however, the true position along the column becomes impossible to ascertain with complete confidence. Fig. S19 shows that as long as vertebrae which represent the first or last 10% of the column were avoided, any data points representing non-mid-trunk vertebrae are likely to lie very close to the morphology represented by mid-trunk vertebrae, and the overall macroevolutionary trends seen should not be affected by such errors.

3.2 Size through time analysis

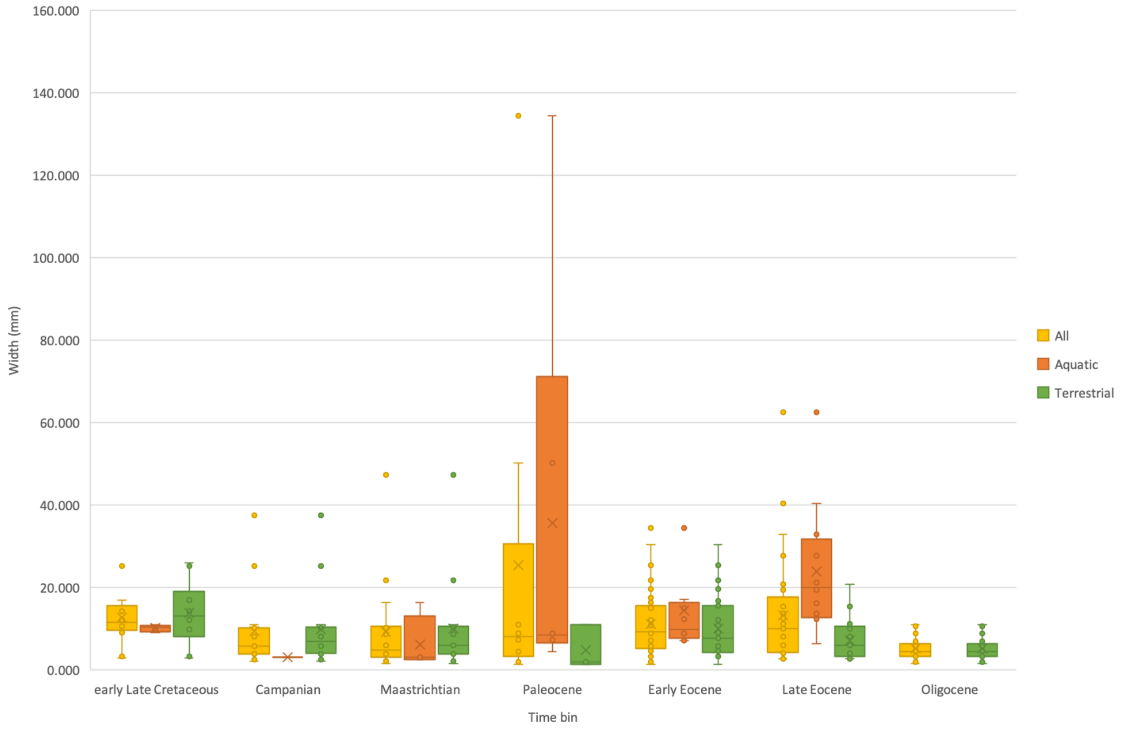

Supplementary Figure 28. Bar plots showing prezygapophyseal widths of mid-trunk snake vertebrae from the early Late Cretaceous to the Oligocene, time binned as in PCA analyses. *n = 155* taxa across all time bins. Middle lines represent median sizes, 'x' represents mean sizes, boxes cover interquartile range, whiskers extend to all datapoints within 1.5x interquartile range, outliers are shown as individual dots.

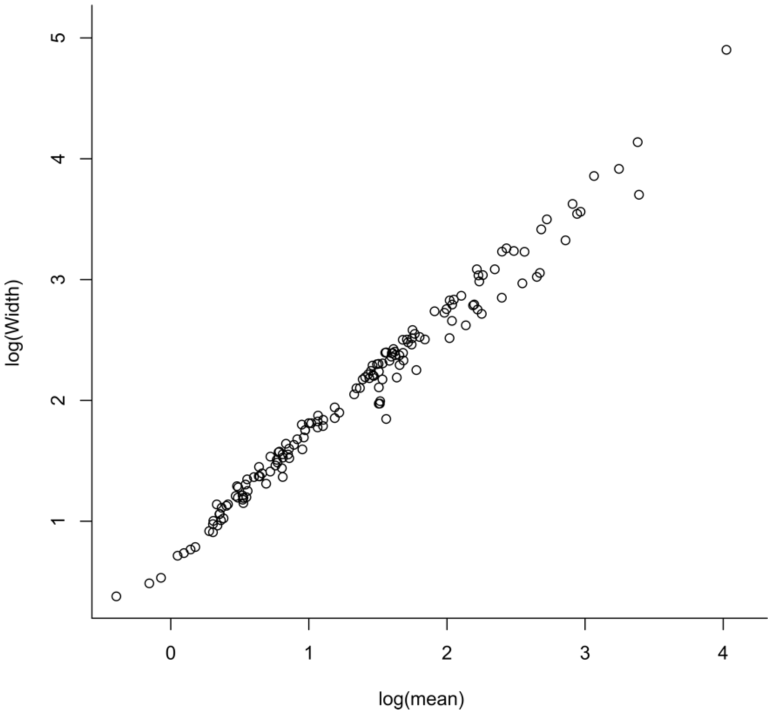

Supplementary Figure 29. Scatter plot showing correlation between the log of widths of vertebrae, and the log of the mean size of all measured elements of vertebrae.

Supplementary Table 4. Chi-squared and *p* values from the Kruskal-Wallis tests comparing differences in size between time bins, for all snake taxa, only aquatic taxa, and only terrestrial taxa, as well as comparing sizes for different inferred ecologies within each time bin. N/A refers to cases where one time bin has n≤1.

**All taxa**	**Chi-squared**	***p***
early Late Cretaceous to Campanian	4.0305	**0.04469**
Campanian to Maastrichtian	0.097578	0.7548
Maastrichtian to Paleocene	0.32002	0.5716
Paleocene to Early Eocene	0.11508	0.7344
Early Eocene to Late Eocene	0.018108	0.893
Late Eocene to Oligocene	11.276	**0.0007853**
**Aquatic taxa**		
early Late Cretaceous to Campanian	N/A	N/A
Campanian to Maastrichtian	N/A	N/A
Maastrichtian to Paleocene	2.9091	0.08808
Paleocene to Early Eocene	0.048804	0.8262
Early Eocene to Late Eocene	3.8343	0.05021
Late Eocene to Oligocene	N/A	N/A

**Terrestrial taxa**		
early Late Cretaceous to Campanian	2.688	0.1011
Campanian to Maastrichtian	0.017177	0.8957
Maastrichtian to Paleocene	1.014	0.3139
Paleocene to Early Eocene	2.2168	0.1365
Early Eocene to Late Eocene	1.8104	0.1785
Late Eocene to Oligocene	1.8465	0.1742
**Terrestrial vs aquatic**		
early Late Cretaceous	1.62	0.2031
Campanian	N/A	N/A
Maastrichtian	0.81	0.3681
Paleocene	1.6667	0.1967
Early Eocene	3.1501	0.07592
Late Eocene	16.035	**$6.218e^{-5}$**
Oligocene	N/A	N/A

Supplementary Information 4: Biogeography

4.1 Dispersal multipliers

Supplementary Table 5. Manual dispersal multipliers tables for all 3 time bins used in BioGeoBEARs analyses; (A) root to 66 Ma, (B) 66 to 20 Ma, and (C) 20 Ma to Recent.

A. Time slice 1: Root to 66 Ma

	Africa	Madagascar	Europe	Asia	India	Oceania	North America	South America	Caribbean
Africa	1	0.8	0.9	0.9	0.2	0.1	0.1	0.1	0.0001
Madagascar	0.8	1	0.1	0.1	0.1	0.1	0.1	0.1	0.0001
Europe	0.9	0.1	1	0.9	0.5	0.1	0.1	0.1	0.0001
Asia	0.9	0.1	0.9	1	0.9	0.8	0.9	0.1	0.0001
India	0.2	0.1	0.5	0.9	1	0.2	0.1	0.1	0.0001
Oceania	0.1	0.1	0.1	0.8	0.2	1	0.1	0.1	0.0001
North America	0.1	0.1	0.1	0.9	0.1	0.1	1	0.8	0.0001
South America	0.1	0.1	0.1	0.1	0.1	0.1	0.8	1	0.0001
Caribbean	0.0001	0.0001	0.0001	0.0001	0.0001	0.0001	0.0001	0.0001	0.0001

B. Time slice 2: 66 Ma to 20 Ma

	Africa	Madagascar	Europe	Asia	India	Oceania	North America	South America	Caribbean
Africa	1	0.8	8	0.7	0.6	0.1	0.2	0.5	0.2
Madagascar	0.8	1	0.1	0.1	0.2	0.1	0.1	0.1	0.1
Europe	0.8	0.1	1	0.8	0.1	0.1	0.8	0.1	0.1
Asia	0.7	0.1	0.8	1	0.8	0.2	0.9	0.1	0.1
India	0.6	0.2	0.1	0.8	1	0.1	0.1	0.1	0.1
Oceania	0.1	0.1	0.1	0.2	0.1	1	0.1	0.1	0.1
North America	0.2	0.1	0.8	0.9	0.1	0.1	1	0.8	0.9
South America	0.5	0.1	0.1	0.1	0.1	0.1	0.8	1	0.8
Caribbean	0.2	0.1	0.1	0.1	0.1	0.1	0.9	0.8	1

C. Time slice 3: 20 Ma to Recent

	Africa	Madagascar	Europe	Asia	India	Oceania	North America	South America	Caribbean
Africa	1	0.8	0.8	0.5	0.7	0.1	0.2	0.8	0.2
Madagascar	0.8	1	0.1	0.1	0.9	0.1	0.1	0.1	0.1
Europe	0.8	0.1	1	0.8	0.1	0.1	0.9	0.1	0.2
Asia	0.5	0.1	0.8	1	0.1	0.1	0.1	0.1	0.1
India	0.7	0.9	0.1	0.1	1	0.5	0.1	0.1	0.1
Oceania	0.1	0.1	0.1	0.1	0.5	1	0.1	0.2	0.1
North America	0.2	0.1	0.9	0.1	0.1	0.1	1	0.8	0.9
South America	0.8	0.1	0.1	0.1	0.1	0.2	0.8	1	0.7
Caribbean	0.2	0.1	0.2	0.1	0.1	0.1	0.9	0.7	1

Areas allowed - as the Caribbean only emerged from below the sea level in the Cenozoic, the model was adjusted such that the Caribbean was not a possible geographical location for any taxa in time bin 1.

4.2 Statistical results

Supplementary Table 6. Likelihood, parameter and AICc values for all models run in BioGeoBEARs.

**Model**	**LnL**	**Number of parameters**	**d**	**e**	**j**	**AICc**	**AICc_wt**
**DEC**	-322.6	2	0.005	3.38E-3	N/A	649.2	2.6E-16
**DEC + J**	-287.8	3	0.003	1.00E-12	0.078	581.9	1.1E-1
**DIVALIKE**	-323.4	2	0.006	2.69E-5	N/A	650.9	1.1E-16
**DIVALIKE + J**	-286.2	3	0.003	1.00E-12	0.067	578.7	5.6E-1
**BAYAREALIKE**	-353.9	2	0.006	2.77E-2	N/A	711.8	6.8E-30
**BAYAREALIKE + J**	-286.8	3	0.002	1.00E-7	0.089	579.7	3.3E-1

Supplementary references

1. Zheng, Y. & Wiens, J. J. Combining phylogenomic and supermatrix approaches, and a time-calibrated phylogeny for squamate reptiles (lizards and snakes) based on 52 genes and 4162 species. *Mol. Phylogenet. Evol.* **94**, 537–547 (2016).
2. Zheng, Y. & Wiens, J. J. Data from: Combining phylogenomic and supermatrix approaches, and a time-calibrated phylogeny for squamate reptiles (lizards and snakes) based on 52 genes and 4162 species. *DataDryad* (2016). doi:https://doi.org/10.5061/dryad.tv055
3. Lartillot, N., Lepage, T. & Blanquart, S. PhyloBayes 3: a Bayesian software package for phylogenetic reconstruction and molecular dating. **25**, 2286–2288 (2009).
4. Maddison, W. P. & Maddison, D. R. Mesquite: a modular system for evolutionary analysis. (2016).
5. Head, J. J. Fossil calibration dates for molecular phylogenetic analysis of snakes 1: Serpentes, Alethinophidia, Boidae, Pythonidae. *Palaeontol. Electron.* **18**, 1–17 (2015).
6. Head, J. J., Mahlow, K. & Müller, J. Fossil calibration dates for molecular phylogenetic analysis of snakes 2: Caenophidia, Colubroidea, Elapoidea, Colubridae. *Palaeontol. Electron.* **19**, 1–21 (2016).
7. Yang, Z. PAML 4 : Phylogenetic Analysis by Maximum Likelihood. *Mol. Biol. Evol.* **24**, 1586–1591 (2007).
8. dos Reis, M., Donoghue, P. C. J. & Yang, Z. Bayesian molecular clock dating of species divergences in the genomics era. *Nat. Rev.* **17**, 71–80 (2015).
9. Morris, J. L. *et al.* The timescale of early land plant evolution. *Proc. Natl. Acad. Sci.* **115**, E2274–E2283 (2018).
10. Lawing, A. M., Head, J. J. & Polly, P. D. The ecology of morphology: the ecometrics of locomotion and macroenvironment in North American snakes. in *Paleontology in Ecology and Conservation* (ed. Louys, J.) 117–146 (Springer Earth System Sciences, 2012). doi:10.1007/978-3-642-25038-5
11. Rage, J.-C., Vullo, R. & Néraudeau, D. The mid-Cretaceous snake Simoliophis rochebrunei Sauvage, 1880 (Squamata: Ophidia) from its type area (Charentes, southwestern France): Redescription, distribution, and palaeoecology. *Cretac. Res.* **58**, 234–253 (2016).
12. Houssaye, A. *et al.* New highlights about the enigmatic marine snake *Palaeophis maghrebianus* (Palaeophiidae; Palaeophiinae) from the Ypresian (Lower Eocene) phosphates of Morocco. *Palaeontology* **56**, 647–661 (2013).
13. Klein, C. G., Longrich, N. R., Ibrahim, N., Zouhri, S. & Martill, D. M. A new basal snake from the mid-Cretaceous of Morocco. *Cretac. Res.* **72**, 134–141 (2017).
14. Head, J. J. *et al.* Giant boid snake from the Palaeocene neotropics reveals hotter past equatorial temperatures. *Nature* **457**, 715–717 (2009).
15. Mayer, M. missRanger: fast imputation of missing values. R package version 2.1.0 (2019).
16. Hammer, Ø., Harper, D. A. T. & Ryan, P. D. PAST: Paleontological Statistics Software Package for Education and Data Analysis. *Palaeontol. Electron.* **4**, 9pp (2001).
17. Wills, M. A. Crustacean disparity through the Phanerozoic: comparing morphological and stratigraphic data. *Biol. J. Linn. Soc.* **65**, 455–500 (1998).
18. Matzke, N. Founder-event speciation in BioGeoBEARS package dramatically improves likelihoods and alters parameter inference in Dispersal-Extinction-Cladogenesis (DEC) analyses. *Front. Biogeogr.* **4**, 210 (2012).
19. Matzke, N. Probabilistic historical biogeography: new models for founder-event speciation, imperfect detection, and fossils allow improved accuracy and model-testing. *Front. Biogeogr.* **5**, 242–248 (2013).

20. Uetz, P., Freed, P. & Hosek, J. The Reptile Database. (2020). Available at: http://www.reptile-database.org/.
21. Scotese, C. R. Paleomap Project. www.scotese.com (2002).
22. Parham, J. F. *et al.* Best practices for justifying fossil calibrations. *Syst. Biol.* **61**, 346–359 (2012).
23. dos Reis, M. *et al.* Phylogenomic datasets provide both precision and accuracy in estimating the timescale of placental mammal phylogeny. *Proc. R. Soc. B Biol. Sci.* **279**, 3491–3500 (2012).
24. Wright, T. F. *et al.* A multilocus molecular phylogeny of the parrots (Psittaciformes): support for a Gondwanan origin during the Cretaceous. *Mol. Biol. Evol.* **25**, 2141–2156 (2008).
25. Vidal, N. *et al.* Blindsnake evolutionary tree reveals long history on Gondwana. *Biol. Lett.* **6**, 558–561 (2010).
26. Benton, M. J. *et al.* Constraints on the timescale of animal evolutionary history. *Palaeontol. Electron.* **18**, 1–106 (2015).
27. Budd, G. E. & Mann, R. P. The dynamics of stem and crown groups. *Sci. Adv.* **6**, eaaz1626 (2020).
28. Raup, D. M. & Sepkoski, J. J. Mass extinctions in the marine fossil record. *Science (80-.).* **215**, 1501–1503 (1982).
29. Bambach, R. K. Phanerozoic Biodiversity Mass Extinctions. *Annu. Rev. Earth Planet. Sci.* **34**, 127–155 (2006).
30. Reeder, T. W. *et al.* Integrated analyses resolve conflicts over squamate reptile phylogeny and reveal unexpected placements for fossil taxa. *PLoS One* **10**, 1–22 (2015).
31. Vidal, N. & Hedges, S. B. The molecular evolutionary tree of lizards, snakes, and amphisbaenians. *Comptes Rendus - Biol.* **332**, 129–139 (2009).
32. Pyron, R. A., Burbrink, F. T. & Wiens, J. J. A phylogeny and revised classification of Squamata, including 4161 species of lizards and snakes. *BMC Evol. Biol.* **13**, 93 (2013).
33. Harrington, S. M. & Reeder, T. W. Phylogenetic inference and divergence dating of snakes using molecules, morphology and fossils: new insights into convergent evolution of feeding morphology and limb reduction. *Biol. J. Linn. Soc.* **121**, 379–394 (2017).
34. Hsiang, A. Y. *et al.* The origin of snakes: revealing the ecology, behavior, and evolutionary history of early snakes using genomics, phenomics, and the fossil record. *BMC Evol. Biol.* **15**, 87 (2015).
35. Gauthier, J. A., Kearney, M., Maisano, J. A., Rieppel, O. & Behlke, A. D. B. Assembling the Squamate Tree of Life: Perspectives from the phenotype and the fossil record. *Bull. Peabody Museum Nat. Hist.* **53**, 3–308 (2012).
36. Conrad, J. L. Phylogeny and systematics of Squamata (Reptilia) based on morphology. *Bull. Am. Museum Nat. Hist.* **310**, 1–182 (2008).
37. Martill, D. M., Tischlinger, H. & Longrich, N. R. A four-legged snake from the Early Cretaceous of Gondwana. *Science (80-.).* **349**, 416–419 (2015).
38. Longrich, N. R., Bhullar, B.-A. S. & Gauthier, J. A. A transitional snake from the Late Cretaceous period of North America. *Nature* **488**, 205–208 (2012).
39. Flynn, J. J., Parrish, J. M., Rakotosamimanana, B., Simpson, W. F. & Wyss, A. R. A Middle Jurassic mammal from Madagascar. *Nature* **401**, 57–60 (1999).
40. Cohen, K., Finney, S., Gibbard, P. & Fan, J.-X. The ICS international chronostratigraphic chart. *Episodes* **36**, 199–204 (2013).
41. Close, R. A., Friedman, M., Lloyd, G. T. & Benson, R. B. J. Evidence for a Mid-Jurassic adaptive radiation in mammals. *Curr. Biol.* **25**, 2137–2142 (2015).
42. Luo, Z.-X., Yuan, C.-X., Meng, Q.-J. & Ji, Q. A Jurassic eutherian mammal and divergence of marsupials and placentals. *Nature* **476**, 442–445 (2011).

43. Fox, R. C. & Scott, C. S. A new, early Puercan (earliest Paleocene) species of *Purgatorius* (Plesiadapiformes, Primates) from Saskatchewan, Canada. *J. Paleontol.* **85**, 537–548 (2011).
44. Clemens, W. A. *Purgatorius* (Plesiadapiformes, Primates?, Mammalia), a Paleocene immigrant into northeastern Montana: stratigraphic occurrences and incisor proportions. *Bull. Carnegie Museum Nat. Hist.* 3–13 (2004).
45. Chester, S. G. B., Bloch, J. I., Boyer, D. M. & Clemens, W. A. Oldest known euarchontan tarsals and affinities of Paleocene Purgatorius to Primates. *Proc. Natl. Acad. Sci.* **112**, 1487–1492 (2015).
46. Sprain, C. J., Renne, P. R., Wilson, G. P. & Clemens, W. A. High-resolution chronostratigraphy of the terrestrial Cretaceous-Paleogene transition and recovery interval in the Hell Creek region, Montana. *Bull. Geol. Soc. Am.* **127**, 393–409 (2015).
47. Dinarès-Turell, J. *et al.* Untangling the Palaeocene climatic rhythm: An astronomically calibrated Early Palaeocene magnetostratigraphy and biostratigraphy at Zumaia (Basque basin, northern Spain). *Earth Planet. Sci. Lett.* **216**, 483–500 (2003).
48. Archibald, J. D., Zhang, Y., Harper, T. & Cifelli, R. L. *Protungulatum*, Confirmed Cretaceous Occurrence of an Otherwise Paleocene Eutherian (Placental?) Mammal. *J. Mamm. Evol.* **18**, 153–161 (2011).
49. Joyce, W. G., Parham, J. F., Lyson, T. R., Warnock, R. C. M. & Donoghue, P. C. J. A divergence dating analysis of turtles using fossil calibrations: an example of best practices. *J. Paleontol.* **87**, 612–634 (2013).
50. Nesbitt, S. J. The early evolution of archosaurs: relationships and the origin of major clades. *Bull. Am. Museum Nat. Hist.* 1–292 (2011).
51. Wu, X.-C. Crocodylians. in *Dinosaur Provincial Park: A spectacular ancient ecosystem revealed* (eds. Currie, P. J. & Koppelhus, E. B.) 277–290 (Indiana University Press, 2005).
52. Wu, X.-C., Brinkman, D. B. & Russell, A. P. A new alligator from the Upper Cretaceous of Canada and the relationships of early eusuchians. *Palaeontology* **39**, 351–375 (1996).
53. Gilmore, C. W. A new fossil alligator from the Hell Creek beds of Montana. *Proc. United States Natl. Museum* **41**, 297–302 (1911).
54. Roberts, E. M., Sampson, S. D., Deino, A. L., Bowring, S. A. & Buchwaldt, R. *The Kaiparowits Formation: a remarkable record of Late Cretaceous terrestrial environments, ecosystems, and evolution in western North America*. *Indiana University Press* (2013).
55. Narváez, I., Brochu, C. A., Escaso, F., Pérez-García, A. & Ortega, F. New crocodyliforms from southwestern Europe and definition of a diverse clade of European Late Cretaceous basal eusuchians. *PLoS One* **10**, e0140679 (2015).
56. Clarke, J. A., Tambussi, C. P., Noriega, J. I., Erickson, G. M. & Ketcham, R. A. Definitive fossil evidence for the extant avian radiation in the Cretaceous. *Nature* **433**, 305–308 (2005).
57. Agnolín, F. L., Egli, F. B., Chatterjee, S., Marsà, J. A. G. & Novas, F. E. Vegaviidae, a new clade of southern diving birds that survived the K/T boundary. *Naturwissenschaften* **104**, 87 (2017).
58. Clarke, J. A. *et al.* Fossil evidence of the avian vocal organ from the Mesozoic. *Nature* **538**, 502–505 (2016).
59. Prum, R. O. *et al.* A comprehensive phylogeny of birds (Aves) using targeted next-generation DNA sequencing. *Nature* **526**, 569–573 (2015).
60. Clarke, J. A. Morphology, phylogenetic taxonomy, and systematics of *Ichthyornis* and *Apatornis* (Avialae, Ornithurae). *Bull. Am. Museum Nat. Hist.* **286**, 1–179 (2004).
61. Longrich, N. R. An ornithurine-dominated avifauna from the Belly River Group (Campanian, Upper Cretaceous) of Alberta, Canada. *Cretac. Res.* **30**, 161–177 (2009).
62. Longrich, N. R., Tokaryk, T. & Field, D. J. Mass extinction of birds at the Cretaceous – Paleogene (K–Pg) boundary. *Proc. Natl. Acad. Sci.* **108**, 15253–15257 (2011).

63. Jones, M. E. H. *et al.* Integration of molecules and new fossils supports a Triassic origin for Lepidosauria (lizards, snakes, and tuatara). *BMC Evol. Biol.* **13**, 208 (2013).
64. Evans, S. E. Crown group lizards (Reptilia, Squamata) from the Middle Jurassic of the British Isles. *Palaeontogr. Abteilung A-Palaozoologie-Stratigraphie* **250**, 123–154 (1998).
65. Evans, S. E. A new anguimorph lizard from the Jurassic and Lower Cretaceous of England. *Palaeontology* **37**, 33–49 (1994).
66. Simões, T. R. *et al.* The origin of squamates revealed by a Middle Triassic lizard from the Italian Alps. *Nature* **557**, 706–709 (2018).
67. Hedges, S. B. & Vidal, N. Lizards, snakes, and amphisbaenians (Squamata). *Time tree life* 383–389 (2009). doi:10.1016/j.crvi.2008.07.010
68. Bauer, A. M., Böhme, W. & Weitschat, W. An Early Eocene gecko from Baltic amber and its implications for the evolution of gecko adhesion. *J. Zool.* **265**, 327–332 (2005).
69. Ritzkowski, S. K-Ar-Altersbestimmungen der bernsteinführenden Sedimente des Samlandes (Paläogen, Bezirk Kaliningrad). *Metalla* **66**, 19–23 (1997).
70. Hutchinson, M. The first fossil pygopod (Squamata, Gekkota), and a review of mandibular variation in living species. *Memoris-queensl. Museum* **41**, 355–366 (1998).
71. Lee, M. S. Y., Oliver, P. M. & Hutchinson, M. N. Phylogenetic uncertainty and molecular clock calibrations: A case study of legless lizards (Pygopodidae, Gekkota). *Mol. Phylogenet. Evol.* **50**, 661–666 (2009).
72. Evans, S. E. & Chure, D. C. Paramacellodid lizard skulls from the Jurassic Morrison Formation at Dinosaur National Monument, Utah. *J. Vertebr. Paleontol.* **18**, 99–114 (1998).
73. Kowallis, B. J., Christiansen, E. H. & Deino, A. L. Age of the Brushy Basin Member of the Morrison Formation, Colorado Plateau, western USA. *Cretac. Res.* **12**, 483–493 (1991).
74. Kowallis, B. J., Britt, B. B., Greenhalgh, B. W. & Sprinkel, D. A. New U-Pb zircon ages from an ash bed in the Brushy Basin Member of the Morrison Formation near Hanksville, Utah. *Utah Geol. Assoc.* **36**, 75–80 (2007).
75. Gradstein, F. M. *The Geologic Timescale*. (2012).
76. Evans, S. E., Raia, P. & Barbera, C. New lizards and rhynchocephalians from the Lower Cretaceous of southern Italy. *Acta Palaeontol. Pol.* **49**, 393–408 (2004).
77. Schmid, D., Leinfelder, R. R. & Schweigert, G. Stratigraphy and palaeoenvironments of the Upper Jurassic of Southern Germany. *Zittelina* **26**, 31–41 (2005).
78. Caldwell, M. W., Nydam, R. L., Palci, A. & Apesteguía, S. The oldest known snakes from the Middle Jurassic-Lower Cretaceous provide insights on snake evolution. *Nat. Commun.* **6**, 5996 (2015).
79. Nydam, R. L. & Fitzpatrick, B. M. The occurrence of *Contogenys* -like lizards in the Late Cretaceous and Early Tertiary of the western interior of the U.S.A. *J. Vertebr. Paleontol.* **29**, 677–701 (2009).
80. Smith, K. T. A new lizard assemblage from the earliest Eocene (zone Wa0) of the Bighorn Basin, Wyoming, USA: Biogeography during the warmest interval of the Cenozoic. *J. Syst. Palaeontol.* **7**, 299–358 (2009).
81. Secord, R. *et al.* Evolution of the earliest horses driven by climate change in the Paleocene-Eocene Thermal Maximum. *Science (80-.).* **335**, 959–962 (2012).
82. Baczynski, A. A. *et al.* Constraining paleohydrologic change during the Paleocene-Eocene Thermal Maximum in the continental interior of North America. *Palaeogeogr. Palaeoclimatol. Palaeoecol.* **465**, 237–246 (2017).
83. Martin, J. E., Hutchinson, M. N., Meredith, R., Case, J. A. & Pledge, N. S. The oldest genus of scincid lizard (Squamata) from the Tertiary Etadunna Formation of South Australia. *J. Herpetol.* **38**, 180–187 (2004).
84. Woodburne, M. O. *et al.* Land Mammal Biostratigraphy and Magnetostratigraphy of the

Etadunna Formation (Late Oligocene) of South Australia. *J. Vertebr. Paleontol.* **13**, 483–515 (1994).
85. Van Couvering, J. A. H. More fossil gerrhosaurs from the Miocene of Kenya (Reptilia: Cordylidae). *Copeia* **1979**, 172–174 (1979).
86. Peppe, D. J. *et al.* Stratigraphic interpretation of the Kulu Formation (Early Miocene, Rusinga Island, Kenya) and its implications for primate evolution. *J. Hum. Evol.* **56**, 447–461 (2009).
87. Brizuela, S. & Albino, A. M. The earliest Tupinambis teiid from South America and its palaeoenvironmental significance. *J. Herpetol.* **38**, 113–119 (2004).
88. Ré, G. H. *et al.* A geochronology for the Sarmiento Formation at Gran Barranca. in *The Paleontology of Gran Barranca: Evolution and Environmental Change through the Middle Cenozoic of Patagonia* (eds. Madden, R. H., Carlini, A. A., Vucetich, M. G. & Kay, R. F.) 46–58 (Cambridge University Press, 2010).
89. Longrich, N. R., Vinther, J., Pyron, R. A., Pisani, D. & Gauthier, J. A. Biogeography of worm lizards (Amphisbaenia) driven by end-Cretaceous mass extinction. *Proc. R. Soc. London B Biol. Sci.* **282**, 20143034 (2015).
90. Sullivan, R. M. A new middle Paleocene (Torrejonian) rhineurid amphisbaenian, Plesiorhineura tsentasi new genus, new species, from the San Juan Basin, New Mexico. *J. Paleontol.* **59**, 1481–1485 (1985).
91. Lofgren, D. L., Lillegraven, J. A., Clemens, W. A., Gingerich, P. D. & Williamson, T. E. Paleocene Biochronology: The Puercan Through Clarkforkian Land Mammal Ages. in *Late Cretaceous and Cenozoic Mammals of North America: Biostratigraphy and Geochronology* (ed. Woodburne, M. O.) 43–105 (Columbia University Press, 2004).
92. Charig, A. J. & Gans, C. Two new amphisbaenians from the Lower Miocene of Kenya. *Bull. Br. Museum, Nat. Hist. Geol.* **46**, 19–36 (1990).
93. Gao, K.-Q. & Hou, L.-H. Systematics and taxonomic diversity of squamates from the Upper Cretaceous Djadochta Formation, Bayan Mandahu, Gobi Desert, People's Republic of China. *Can. J. Earth Sci.* **33**, 578–598 (1996).
94. Dashzeveg, D. *et al.* New stratigraphic subdivision, depositional environment, and age estimate for the Upper Cretaceous Djadokhta Formation, Southern Ulan Nur Basin, Mongolia. *Am. Museum Novit.* **3498**, 1–31 (2005).
95. Averianov, A. O. & Danilov, I. G. Agamid lizards (Reptilia, Sauria, Agamidae) from the Early Eocene of Kyrgyzstan. *Neues Jahrb. fur Geol. und Palaeontol. Monatshefte* **12**, 739–750 (1996).
96. Alifanov. Revision of *Tinosaurus asiaticus* Gilmore (Agamidae. *Paleontol. J.* **27**, 148–154 (1993).
97. Smith, K. T. Eocene lizards of the clade *Geiseltaliellus* from Messel and Geiseltal, Germany, and the early radiation of Iguanidae (Reptilia: Squamata). *Bull. Peabody Museum Nat. Hist.* **50**, 219–306 (2009).
98. Nydam, R. L. A new taxon of helodermetid-like lizard from the Albian-Cenomanian of Utah. *J. Vertebr. Paleontol.* **20**, 285–294 (2000).
99. Longrich, N. R., Bhullar, B.-A. S. & Gauthier, J. A. Mass extinction of lizards and snakes at the Cretaceous-Paleogene boundary. *Proc. Natl. Acad. Sci.* **109**, 21396–21401 (2012).
100. Hoffstetter, R. Un saurien helodermatidé (Eurheloderma gallicum nov. gen. et sp.) dans la faune fossile des Phosphorites du Quercy. *Bull. la Société Géologique Fr.* **7**, 775–786 (1957).
101. Rieppel, O. & Grande, L. The anatomy of the fossil varanid lizard *Saniwa ensidens* Leidy, 1870, based on a newly discovered complete skeleton. *J. Paleontol.* **81**, 643–665 (2007).
102. Smith, M. E., Singer, B. & Carroll, A. 40Ar/39Ar geochronology of the Eocene Green River Formation, Wyoming. *Bull. Geol. Soc. Am.* **115**, 549–565 (2003).
103. Estes, R. Fish, amphibians and reptiles from the Etadunna Formation, Miocene of South

Australia. *Aust. Zool.* **21**, 335–343 (1984).
104. Pianka, E. R. & King, D. R. *Varanoid lizards of the world*. (Indiana University Press, 2004).
105. Estes, R. Fossil vertebrates from the Late Cretaceous Lance Formation, Eastern Wyoming. *Univ. Calif. Publ. Dep. Geol. Sci.* **49**, 1–180 (1964).
106. Sahni, A. The vertebrate fauna of the Judith River Formation, Montana. *Bull. Am. Museum Nat. Hist.* **147**, 321–412 (1972).
107. Goodwin, M. B. & Deino, A. L. The first radiometric ages from the Judith River Formation (Upper Cretaceous), Hill County, Montana. *Can. J. Earth Sci.* **26**, 1384–1391 (1989).
108. Scanferla, A., Zaher, H., Novas, F. E., de Muizon, C. & Céspedes, R. A new snake skull from the Paleocene of Bolivia sheds light on the evolution of macrostomatans. *PLoS One* **8**, e57583 (2013).
109. Smith, K. T. New constraints on the evolution of the snake clades Ungaliophiinae, Loxocemidae and Colubridae (Serpentes), with comments on the fossil history of erycine boids in North America. *Zool. Anz.* **252**, 157–182 (2013).
110. Smith, K. T. The evolution of mid-latitude faunas during the Eocene: Late Eocene lizards of the Medicine Pole Hills reconsidered. *Bull. Peabody Museum Nat. Hist.* **52**, 3–105 (2011).
111. Scanlon, J. D. *Montypythonoides*: the Miocene snake *Morelia riversleighensis* (Smith & Plane, 1985) and the geographical origin of pythons. *Mem. Assoc. Australas. Palaeontol.* **25**, 1–35 (2001).
112. Rage, J.-C. *et al.* A diverse snake fauna from the early Eocene of Vastan Lignite Mine, Gujarat, India. *Acta Palaeontol. Pol.* **53**, 391–403 (2008).
113. Rage, J.-C. Les serpents des Phosphorites du Quercy. *Palaeovertebrata* **6**, 274–303 (1974).
114. Holman, J. A. *Texasophis galbreathi*, new species, the earliest New World colubrid snake. *J. Vertebr. Paleontol.* **3**, 223–225 (1984).
115. Kuch, U., Müller, J., Mödden, C. & Mebs, D. Snake fangs from the Lower Miocene of Germany: evolutionary stability of perfect weapons. *Naturwissenschaften* **93**, 84–87 (2006).
116. Scanlon, J. D., Lee, M. S. Y. & Archer, M. Serpents élapidés (Squamata, Colubroidea) du Tertiaire moyen de Riversleigh, Nord de l'Australie: Étapes précoces d'une radiation adaptive répandue sur un continent entier. *Geobios* **36**, 573–601 (2003).
117. Ivanov, M. The first European pit viper from the Miocene of Ukraine. *Acta Palaeontol. Pol.* **44**, 327–334 (1999).
118. Agustí, J. *et al.* A calibrated mammal scale for the Neogene of Western Europe. State of the art. *Earth Sci. Rev.* **52**, 247–260 (2001).
119. de Zoeten, R. & Mann, P. Structural geology and Cenozoic tectonic history of the southeastern termination of the Cordillera Central, Dominican Republic. *Geol. Soc. Am. Spec. Pap.* **262**, 315–336 (1991).
120. Cox, A. Ages of the Galápagos islands. *Patterns Evol. Galapagos Org.* **1**, 1–23 (1983).
121. Christie, D. M. *et al.* Drowned islands downstream from the Galapagos hotspot imply extended speciation times. *Nature* **355**, 246–248 (1992).
122. Rassmann, K. Evolutionary age of the Galápagos iguanas predates the age of the present Galápagos Islands. *Mol. Phylogenet. Evol.* **7**, 158–172 (1997).
123. MacLeod, A. *et al.* Hybridization masks speciation in the evolutionary history of the Galápagos marine iguana. *Proc. R. Soc. B* **282**, 20150425 (2015).
124. Benavides, E., Baum, R., Snell, H. M., Snell, H. L. & Sites, J. W. Island biogeography of Galápagos lava lizards (Tropiduridae: Microlophus): Species diversity and colonization of the archipelago. *Evolution (N. Y).* **63**, 1606–1626 (2009).
125. Poulakakis, N., Russello, M., Geist, D. & Caccone, A. Unravelling the peculiarities of island life: Vicariance, dispersal and the diversification of the extinct and extant giant Galápagos tortoises. *Mol. Ecol.* **21**, 160–173 (2012).

126. Sato, A. *et al.* On the origin of Darwin's Finches. *Mol. Biol. Evol.* **18**, 299–311 (2001).
127. Paparella, I., Palci, A., Nicosia, U. & Caldwell, M. W. A new fossil marine lizard with soft tissues from the Late Cretaceous of southern Italy. *R. Soc. open Sci.* **5**, 172411 (2018).
128. Evans, S. E. & Wang, Y. The Early Cretaceous lizard *Dalinghosaurus* from China. *Acta Palaeontol. Pol.* **50**, 725–742 (2005).
129. Garberoglio, F. F. *et al.* New skulls and skeletons of the Cretaceous legged snake Najash , and the evolution of the modern snake body plan. *Sci. Adv.* **5**, eaax5833 (2019).
130. Caldwell, M. W. & Lee, M. S. Y. A snake with legs from the marine Cretaceous of the Middle East. *Nature* **386**, 705–709 (1997).
131. Rieppel, O., Zaher, H., Tchernov, E. & Polcyn, M. J. The anatomy and relationships of Haasiophis terrasanctus , a fossil snake with well-developed hind limbs from the mid-Cretaceous of the Middle East. *J. Paleontol.* **77**, 536–558 (2003).
132. Apesteguía, S. & Zaher, H. A Cretaceous terrestrial snake with robust hindlimbs and a sacrum. *Nature* **440**, 1037–1040 (2006).
133. Zaher, H. & Scanferla, C. A. The skull of the Upper Cretaceous snake *Dinilysia patagonica* Smith-Woodward, 1901, and its phylogenetic position revisited. *Zool. J. Linn. Soc.* **164**, 194–238 (2012).
134. Palci, A., Caldwell, M. W. & Nydam, R. L. Reevaluation of the anatomy of the Cenomanian (Upper Cretaceous) hind-limbed marine fossil snakes *Pachyrhachis*, *Haasiophis*, and *Eupodophis*. *J. Vertebr. Paleontol.* **33**, 1328–1342 (2013).
135. Mulcahy, D. G. *et al.* Estimating divergence dates and evaluating dating methods using phylogenomic and mitochondrial data in squamate reptiles. *Mol. Phylogenet. Evol.* **65**, 974–991 (2012).
136. Hipsley, C. A. & Müller, J. Beyond fossil calibrations: Realities of molecular clock practices in evolutionary biology. *Front. Genet.* **5**, 1–11 (2014).
137. Sues, H.-D. & Olsen, P. E. Triassic vertebrates of Gondwanan aspect from the Richmond basin of Virginia. *Science (80-.).* **249**, 1020–1023 (1990).
138. Fraser, N. C. & Benton, M. J. The Triassic reptiles *Brachyrhinodon* and *Polysphenodon* and the relationships of the sphenodontids. *Zool. J. Linn. Soc.* **96**, 413–445 (1989).
139. Ho, S. Y. W. & Duchêne, S. Molecular-clock methods for estimating evolutionary rates and timescales. *Mol. Ecol.* **23**, 5947–5965 (2014).
140. Brown, R. P. & Yang, Z. Rate variation and estimation of divergence times using strict and relaxed clocks. *BMC Evol. Biol.* **11**, 271 (2011).
141. Duchêne, S., Lanfear, R. & Ho, S. Y. W. The impact of calibration and clock-model choice on molecular estimates of divergence times. *Mol. Phylogenet. Evol.* **78**, 277–289 (2014).
142. Tao, Q., Tamura, K., Battistuzzi, F. U. & Kumar, S. A machine learning method for detecting autocorrelation of evolutionary rates in large phylogenies. *Mol. Biol. Evol.* **36**, 811–824 (2019).
143. Bromham, L. *et al.* Bayesian molecular dating: opening up the black box. *Biol. Rev.* **93**, 1165–1191 (2018).
144. Ho, S. Y. W. An examination of phylogenetic models of substitution rate variation among lineages. *Biol. Lett.* **5**, 421–424 (2009).
145. Li, W.-H., Ellsworth, D. L., Krushkal, J., Chang, B. H.-J. & Hewett-Emmett, D. Rates of nucleotide substitution in primates and rodents and the generation–time effect hypothesis. *Mol. Phylogenet. Evol.* **5**, 182–187 (1996).
146. Welch, J. J., Bininda-Edmonds, O. R. P. & Bromham, L. Correlates of substitution rate variation in mammalian protein-coding sequences. *BMC Evol. Biol.* **8**, 53 (2008).
147. Thomas, J. A., Welch, J. J., Lanfear, R. & Bromham, L. A Generation Time Effect on the rate of molecular evolution in invertebrates. *Mol. Biol. Evol.* **27**, 1173–1180 (2010).

148. Gillooly, J. F., Allen, A. P., West, G. B. & Brown, J. H. The rate of DNA evolution: Effects of body size and temperature on the molecular clock. *Proc. Natl. Acad. Sci.* **102**, 140–145 (2005).
149. Martin, A. P. & Palumbi, S. R. Body size, metabolic rate, generation time, and the molecular clock. *Proc. Natl. Acad. Sci.* **90**, 4087–4091 (1993).
150. Gillman, L. N., Keeling, D. J., Ross, H. A. & Wright, S. D. Latitude , elevation and the tempo of molecular evolution in mammals. *Proc. R. Soc. B* **276**, 3353–3359 (2009).
151. Gillman, L. N., McCowan, L. S. C. & Wright, S. D. The tempo of genetic evolution in birds: body mass and climate effects. *J. Biogeogr.* **39**, 1567–1572 (2012).
152. Wright, S. D., Gillman, L. N., Ross, H. A. & Keeling, D. J. Energy and the tempo of evolution in amphibians. *Glob. Ecol. Biogeogr.* **19**, 733–740 (2010).
153. Wright, S. D., Ross, H. A., Keeling, D. J., McBride, P. & Gillman, L. N. Thermal energy and the rate of genetic evolution in marine fishes. *Evol. Ecol.* **25**, 525–530 (2011).
154. Rolland, J., Loiseau, O., Romiguier, J. & Salamin, N. Molecular evolutionary rates are not correlated with temperature and latitude in Squamata: an exception to the metabolic theory of ecology? *BMC Evol. Biol.* **16**, 95 (2016).
155. Woolfit, M. & Bromham, L. Population size and molecular evolution on islands. *Proc. R. Soc. B* **272**, 2277–2282 (2005).
156. Woolfit, M. & Bromham, L. Increased rates of sequence evolution in endosymbiotic bacteria and fungi with small effective population sizes. *Mol. Biol. Evol.* **20**, 1545–1555 (2003).
157. Kliman, R. M. *et al.* The population genetics of the origin and divergence of the Drosophila simulans complex species. *Genetics* **156**, 1913–1931 (2000).
158. Zachos, J., Pagani, M., Sloan, L., Thomas, E. & Billups, K. Trends, rhythms, and aberrations in global climate 65 Ma to Present. *Science (80-.).* **292**, 686–694 (2001).
159. Drummond, A. J., Ho, S. Y. W., Phillips, M. J. & Rambaut, A. Relaxed phylogenetics and dating with confidence. *PLoS Biol.* **4**, 699–710 (2006).
160. Lepage, T., Bryant, D., Philippe, H. & Lartillot, N. A general comparison of relaxed molecular clock models. *Mol. Biol. Evol.* **24**, 2669–2680 (2007).
161. Ho, S. Y. W., Duchêne, S. & Duchêne, D. A. Simulating and detecting autocorrelation of molecular evolutionary rates among lineages. *Mol. Ecol.* **15**, 688–696 (2015).
162. Head, J. J. & Polly, P. D. Evolution of the snake body form reveals homoplasy in amniote Hox gene function. *Nature* **520**, 96–89 (2015).
163. Head, J. J. Data from: Evolution of the snake body form reveals homoplasy in amniote Hox gene function. https://doi.org/10.5061/dryad.jq285 (2015).
