# Supplementary material for: Evolution and dispersal of snakes across the Cretaceous-Paleogene mass extinction
Source: Nat Commun. 2021 Sep 14;12:5335. doi: 10.1038/s41467-021-25136-y (PMC8440539; doi:10.1038/s41467-021-25136-y)
Supplement: Supplementary file 2 — Reporting Summary [file 41467_2021_25136_MOESM2_ESM.pdf]

nature research

Corresponding author(s): Nicholas Longrich
Catherine G. Klein

Last updated by author(s): Jun 7, 2021

Reporting Summary

Nature Research wishes to improve the reproducibility of the work that we publish. This form provides structure for consistency and transparency in reporting. For further information on Nature Research policies, see our Editorial Policies and the Editorial Policy Checklist.

Please do not complete any field with "not applicable" or n/a. Refer to the help text for what text to use if an item is not relevant to your study.
For final submission: please carefully check your responses for accuracy; you will not be able to make changes later.

Statistics

For all statistical analyses, confirm that the following items are present in the figure legend, table legend, main text, or Methods section.

n/a	Confirmed	
☐	☒	The exact sample size (*n*) for each experimental group/condition, given as a discrete number and unit of measurement
☐	☒	A statement on whether measurements were taken from distinct samples or whether the same sample was measured repeatedly
☐	☐	The statistical test(s) used AND whether they are one- or two-sided<br>*Only common tests should be described solely by name; describe more complex techniques in the Methods section.*
☐	☒	A description of all covariates tested
☐	☒	A description of any assumptions or corrections, such as tests of normality and adjustment for multiple comparisons
☐	☒	A full description of the statistical parameters including central tendency (e.g. means) or other basic estimates (e.g. regression coefficient) AND variation (e.g. standard deviation) or associated estimates of uncertainty (e.g. confidence intervals)
☐	☒	For null hypothesis testing, the test statistic (e.g. *F*, *t*, *r*) with confidence intervals, effect sizes, degrees of freedom and *P* value noted<br>*Give P values as exact values whenever suitable.*
☐	☒	For Bayesian analysis, information on the choice of priors and Markov chain Monte Carlo settings
☒	☐	For hierarchical and complex designs, identification of the appropriate level for tests and full reporting of outcomes
☒	☐	Estimates of effect sizes (e.g. Cohen's *d*, Pearson's *r*), indicating how they were calculated

Our web collection on statistics for biologists contains articles on many of the points above.

Software and code

Policy information about availability of computer code

Data collection	Image J 1.80
Data analysis	PhyloBayes v.3.3<br>PAML v.4<br>Mesquite v.2.75<br>MCMCTreeR - in R v.1.0<br>BioGeoBEARS v.1.1.1<br>PAST3 v.10 (morphometric analyses)<br>RARE v.1<br>missRanger v.2.1.0

For manuscripts utilizing custom algorithms or software that are central to the research but not yet described in published literature, software must be made available to editors and reviewers. We strongly encourage code deposition in a community repository (e.g. GitHub). See the Nature Research guidelines for submitting code & software for further information.

Data

Policy information about availability of data

All manuscripts must include a data availability statement. This statement should provide the following information, where applicable:

- Accession codes, unique identifiers, or web links for publicly available datasets
- A list of figures that have associated raw data
- A description of any restrictions on data availability

Molecular sequences are available at DOI: https://doi.org/10.5061/dryad.tv055.

Information on extant taxon ranges was gathered from the Reptile Database https://reptile-database.reptarium.cz/.
All other data is from published literature (references included in SI)
All figures have associated raw data, freely available in the SI.

Field-specific reporting

Please select the one below that is the best fit for your research. If you are not sure, read the appropriate sections before making your selection.

☐ Life sciences ☐ Behavioural & social sciences ☒ Ecological, evolutionary & environmental sciences

For a reference copy of the document with all sections, see nature.com/documents/nr-reporting-summary-flat.pdf

Ecological, evolutionary & environmental sciences study design

All studies must disclose on these points even when the disclosure is negative.

Study description	This study consists of a number of different analyses:<br>Phylogenetic analyses: after original construction of a tree in PhyloBayes, different parameterisations were tested in PAML. Each time, at least two chains were run and checked for convergence, and a final sample size of over 2000 was used.<br>Disparity analyses: disparity was estimated based on vertebral measurements, in PAST.<br>Biogeographical analyses: geographical ranges of extant taxa were used to infer biogeographical patterns in the past, accounting for the delayed emergence of the Caribbean and the changing distances between continents, from the Cretaceous to the present.
Research sample	Phylogenetic analyses: an existing dataset of molecular sequences for all squamate species (Zheng & Wiens, 2016) with at least one gene sequenced at the time was used. This was subsampled with a focus on snakes, but maintaining the non-squamate outgroup taxa and other squamate clades, to allow for additional fossil calibrations.<br>Disparity analyses: images of published fossil snake vertebrae were collected, all references available in the supplementary files.<br>Biogeographical analyses: only the ingroup tree was used. All estimations run over 3 time slices and 9 continental areas. Information on extant taxon ranges was gathered from the Reptile Database.
Sampling strategy	Disparity analyses: all snake fossils represented by mid-trunk vertebrae, dating from the mid-Cretaceous to the Oligocene, and published in peer-reviewed literature, were included. Sample sizes in individual time bins, when data was separated by ecological preference in size through time analyses, reached a minimum of three.<br>Phylogenetic analyses: snake taxa were chosen so as to reflect the different families within snakes and all basal nodes, without compromising on the stability of the tree due to low gene sampling. Sampling was not automated, but conducted as objectively as possible
Data collection	Data was purely collected from the literature. All measurements taken for disparity analyses and geographical ranges were collected by a single person, CGK.
Timing and spatial scale	Data collection for disparity analyses occurred in November 2017 to May 2019. Geographical ranges were recorded on 27 April 2018
Data exclusions	Exclusion criteria were pre-established - only some snake vertebrae were excluded from disparity analyses, when the position along the vertebral column could not be established, or specimen was described as juvenile.
Reproducibility	All molecular clock analyses were run at least twice, successfully converging on similar dates. All other analyses can be reproduced successfully.
Randomization	Searches of tree space were started in random locations. For all other analyses, randomization tests were implemented, as we did not have control over the grouping of data - for disparity analyses species were allocated to groups in relation to their temporal range
Blinding	We deal with a single history of life. This is not a manipulative experimental set up or design, so blinding was not relevant.

Did the study involve field work? ☐ Yes ☒ No

Reporting for specific materials, systems and methods

We require information from authors about some types of materials, experimental systems and methods used in many studies. Here, indicate whether each material, system or method listed is relevant to your study. If you are not sure if a list item applies to your research, read the appropriate section before selecting a response.

Materials & experimental systems

n/a	Involved in the study	
☒	☐	Antibodies
☒	☐	Eukaryotic cell lines
☒	☐	Palaeontology and archaeology
☒	☐	Animals and other organisms
☒	☐	Human research participants
☒	☐	Clinical data
☒	☐	Dual use research of concern

Methods

n/a	Involved in the study	
☒	☐	ChIP-seq
☒	☐	Flow cytometry
☒	☐	MRI-based neuroimaging

This checklist template is licensed under a Creative Commons Attribution 4.0 International License, which permits use, sharing, adaptation, distribution and reproduction in any medium or format, as long as you give appropriate credit to the original author(s) and the source, provide a link to the Creative Commons license, and indicate if changes were made. The images or other third party material in this article are included in the article's Creative Commons license, unless indicated otherwise in a credit line to the material. If material is not included in the article's Creative Commons license and your intended use is not permitted by statutory regulation or exceeds the permitted use, you will need to obtain permission directly from the copyright holder. To view a copy of this license, visit http://creativecommons.org/licenses/by/4.0/
